# Supplementary material for: Proteome-wide association studies for blood lipids and comparison with transcriptome-wide association studies
Source: HGG Adv. 2024 Nov 14;6(1):100383. doi: 10.1016/j.xhgg.2024.100383 (PMC11650301; doi:10.1016/j.xhgg.2024.100383)
Supplement: Document S1. Figures S1–S46 and Tables S1 and S2 [file mmc1.pdf]

HGGA, Volume *6*

Supplemental information

Proteome-wide association studies for blood lipids and comparison with transcriptome-wide association studies

Daiwei Zhang, Boran Gao, Qidi Feng, Ani Manichaikul, Gina M. Peloso, Russell P. Tracy, Peter Durda, Kent D. Taylor, Yongmei Liu, W. Craig Johnson, Stacey Gabriel, Namrata Gupta, Joshua D. Smith, Francois Aguet, Kristin G. Ardlie, Thomas W. Blackwell, Robert E. Gerszten, Stephen S. Rich, Jerome I. Rotter, Laura J. Scott, Xiang Zhou, and Seunggeun Lee

Supplementary Materials

S1 Additional results for all lipids

Figure S1: MESA protein prediction model performance.

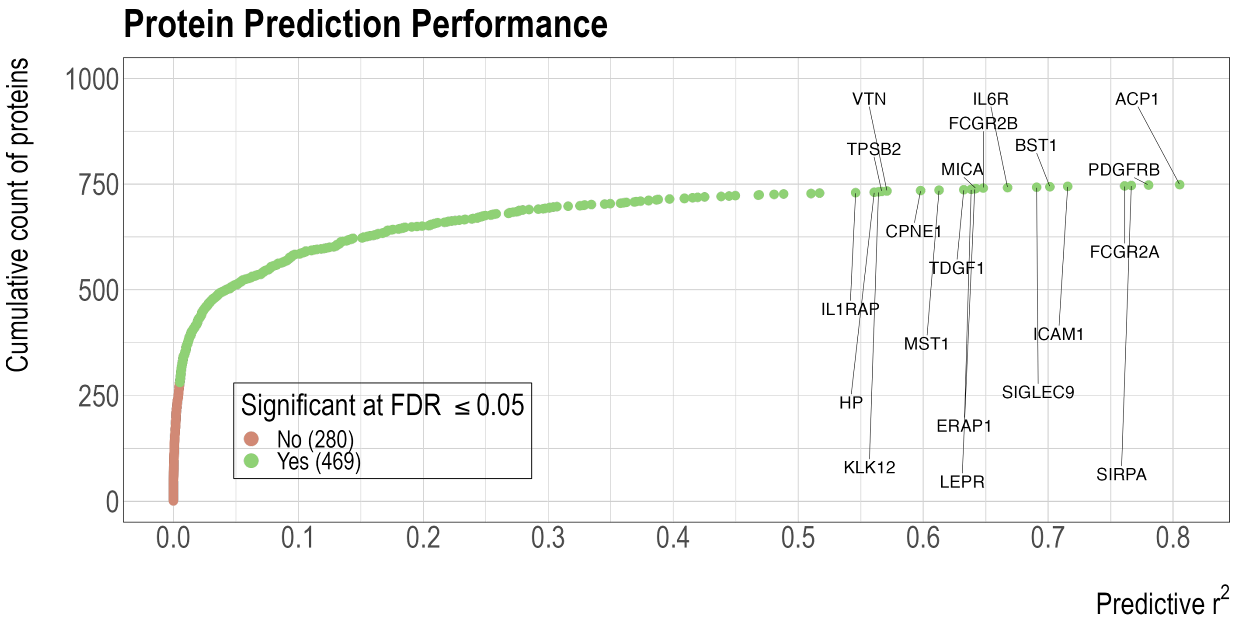

Figure S2: Comparison of APOE's protein and gene expression predictive model weights with the LDL GWAS z-scores of the SNPs. The reference and alternative alleles for GWAS and the predictive models have been aligned and reordered so that all the SNPs have positive GWAS effects. The z-scores are used to compute the weighted average of the model weights (dashed lines), which have the same signs as and are proportional to the predicted effects of protein and gene expression on the GWAS outcome.

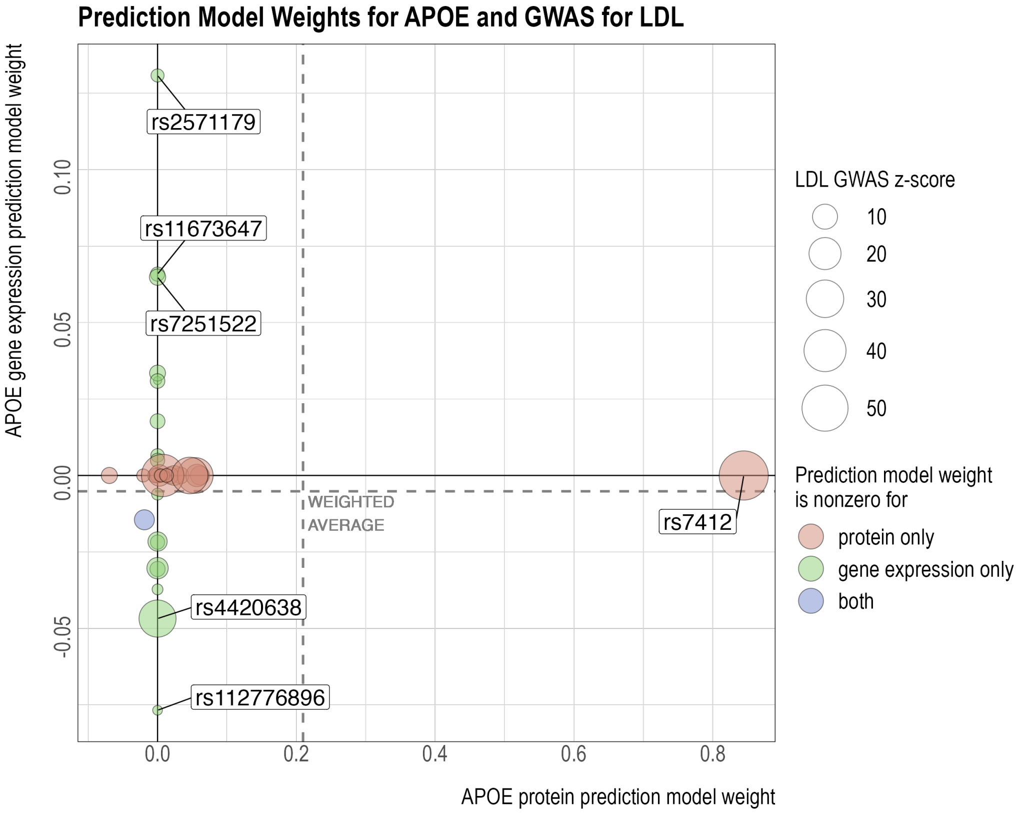

Figure S3: GWAS for LDL and prediction models for FCGR2B's protein and gene expression levels. The reference and alternative alleles for GWAS and the predictive models have been aligned and reordered so that all the SNPs have positive GWAS effects. In the center and bottom panels, the size of the circles indicates the SNP's GWAS z-score. The z-scores are used to compute the weighted average of the model weights (dashed line), which has the same sign as and is proportional to the predicted effect of protein or gene expression on the GWAS outcome.

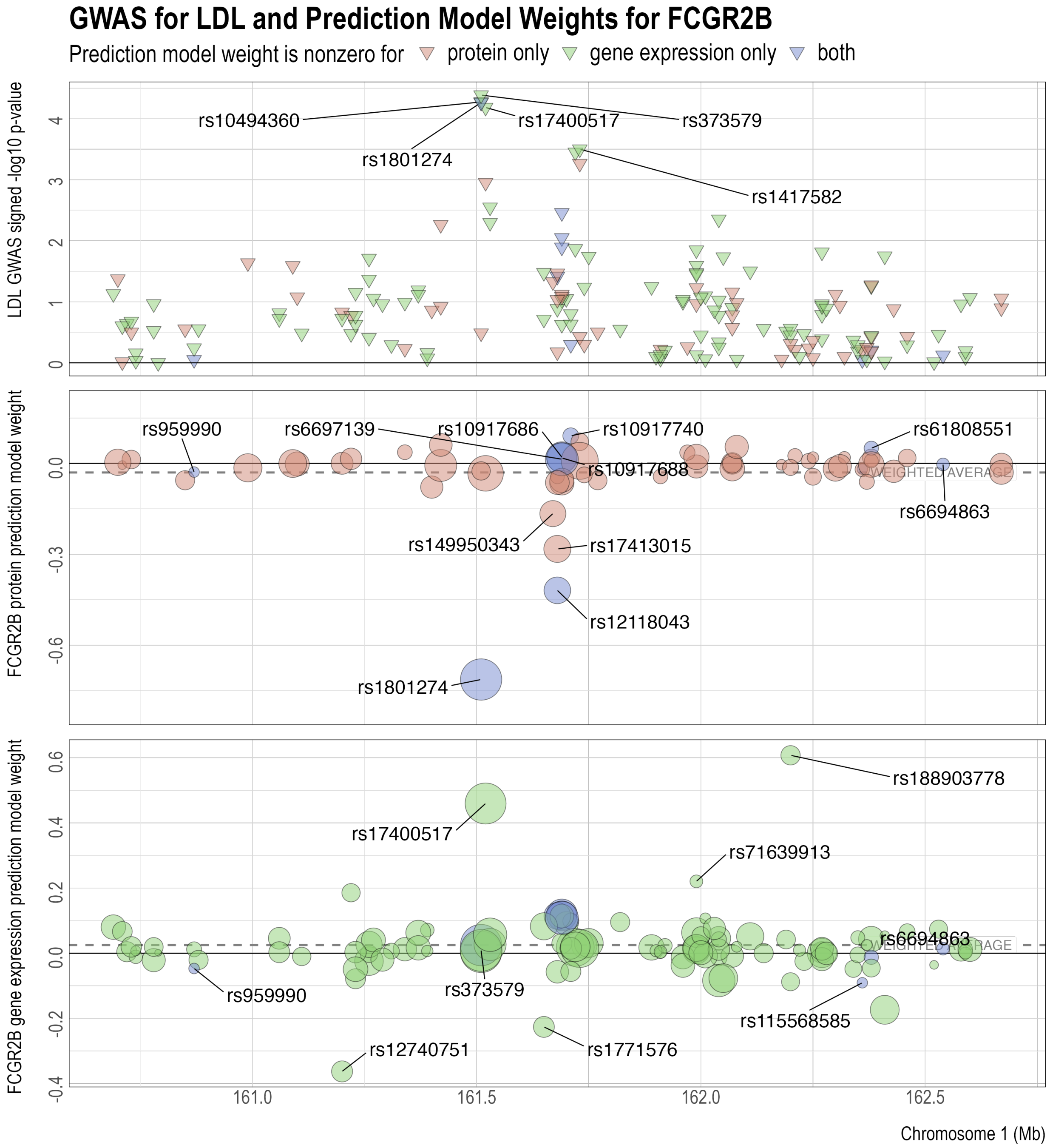

Figure S4: Comparison of FCGR2B's protein and gene expression predictive model weights with the LDL GWAS z-scores of the SNPs. The reference and alternative alleles for GWAS and the predictive models have been aligned and reordered so that all the SNPs have positive GWAS effects. The z-scores are used to compute the weighted average of the model weights (dashed lines), which have the same signs as and are proportional to the predicted effects of protein and gene expression on the GWAS outcome.

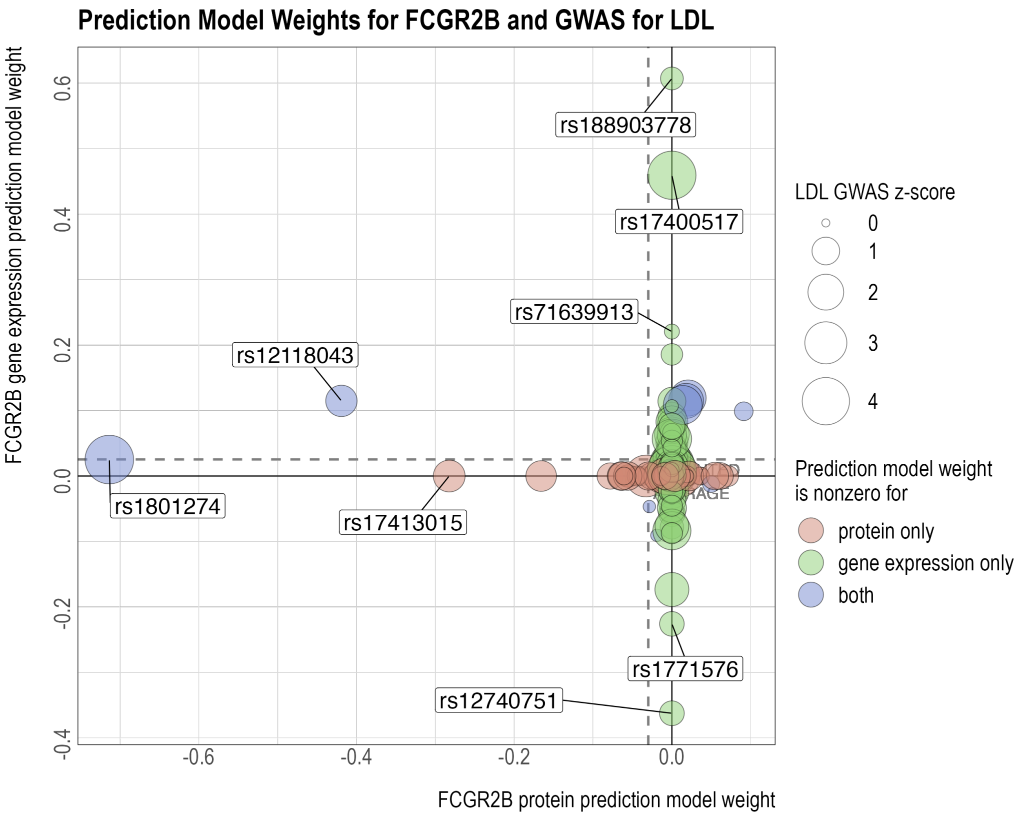

Figure S5: GWAS for LDL and prediction models for LILRB2's protein and gene expression levels. The reference and alternative alleles for GWAS and the predictive models have been aligned and reordered so that all the SNPs have positive GWAS effects. In the center and bottom panels, the size of the circles indicates the SNP's GWAS z-score. The z-scores are used to compute the weighted average of the model weights (dashed line), which has the same sign as and is proportional to the predicted effect of protein or gene expression on the GWAS outcome.

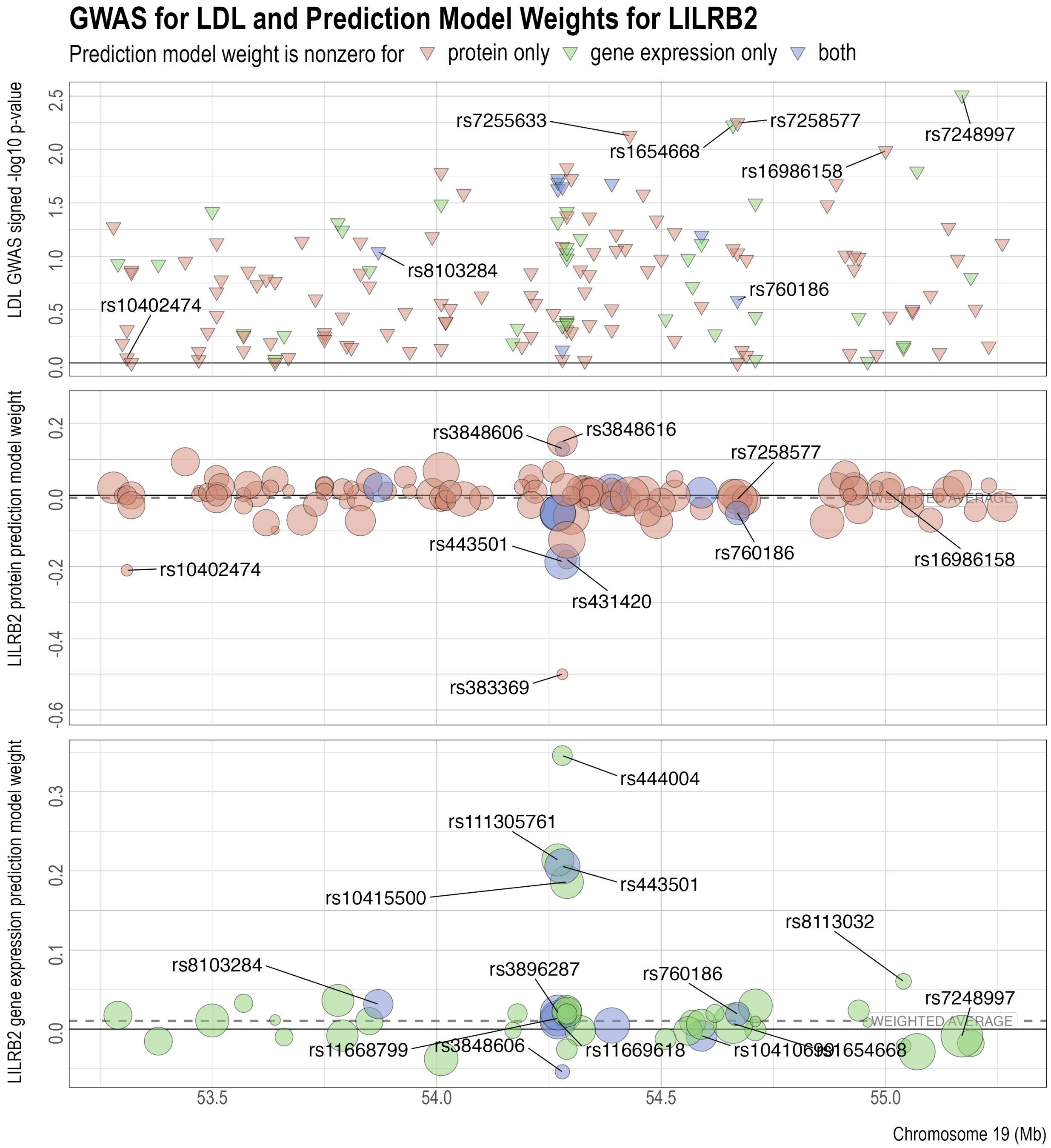

Figure S6: Comparison of LILRB2's protein and gene expression predictive model weights with the LDL GWAS z-scores of the SNPs. The reference and alternative alleles for GWAS and the predictive models have been aligned and reordered so that all the SNPs have positive GWAS effects. The z-scores are used to compute the weighted average of the model weights (dashed lines), which have the same signs as and are proportional to the predicted effects of protein and gene expression on the GWAS outcome.

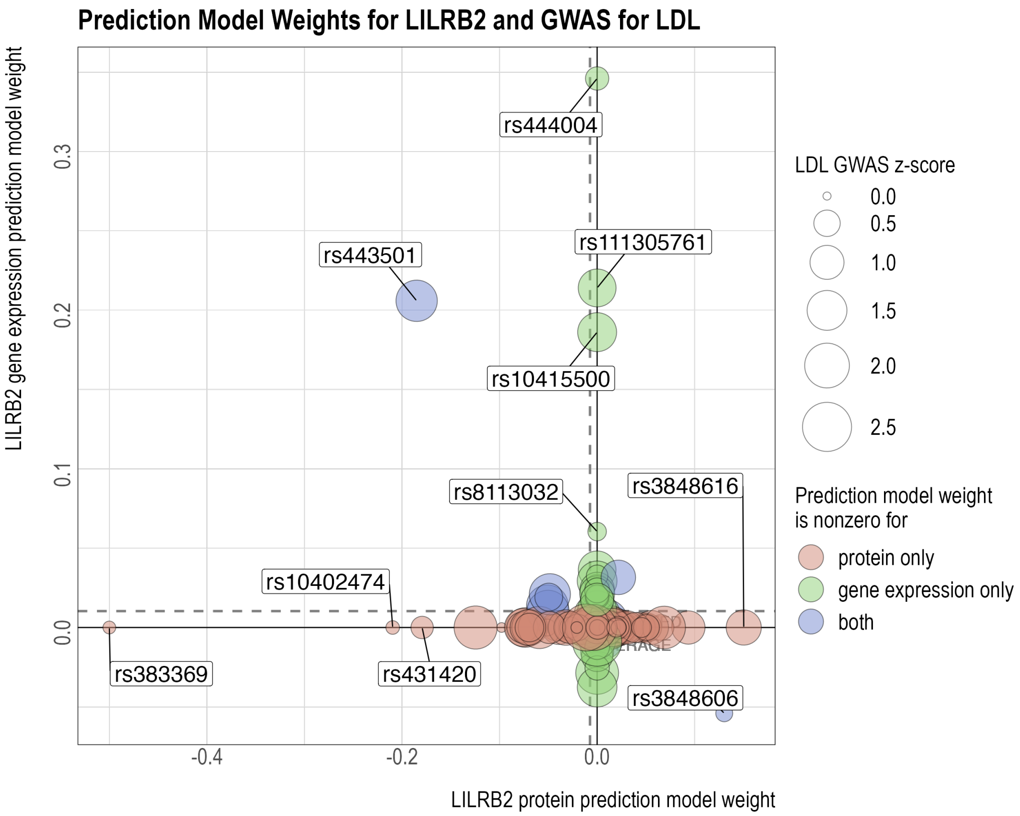

Figure S7: GWAS for LDL and prediction models for MICB's protein and gene expression levels. The reference and alternative alleles for GWAS and the predictive models have been aligned and reordered so that all the SNPs have positive GWAS effects. In the center and bottom panels, the size of the circles indicates the SNP's GWAS z-score. The z-scores are used to compute the weighted average of the model weights (dashed line), which has the same sign as and is proportional to the predicted effect of protein or gene expression on the GWAS outcome.

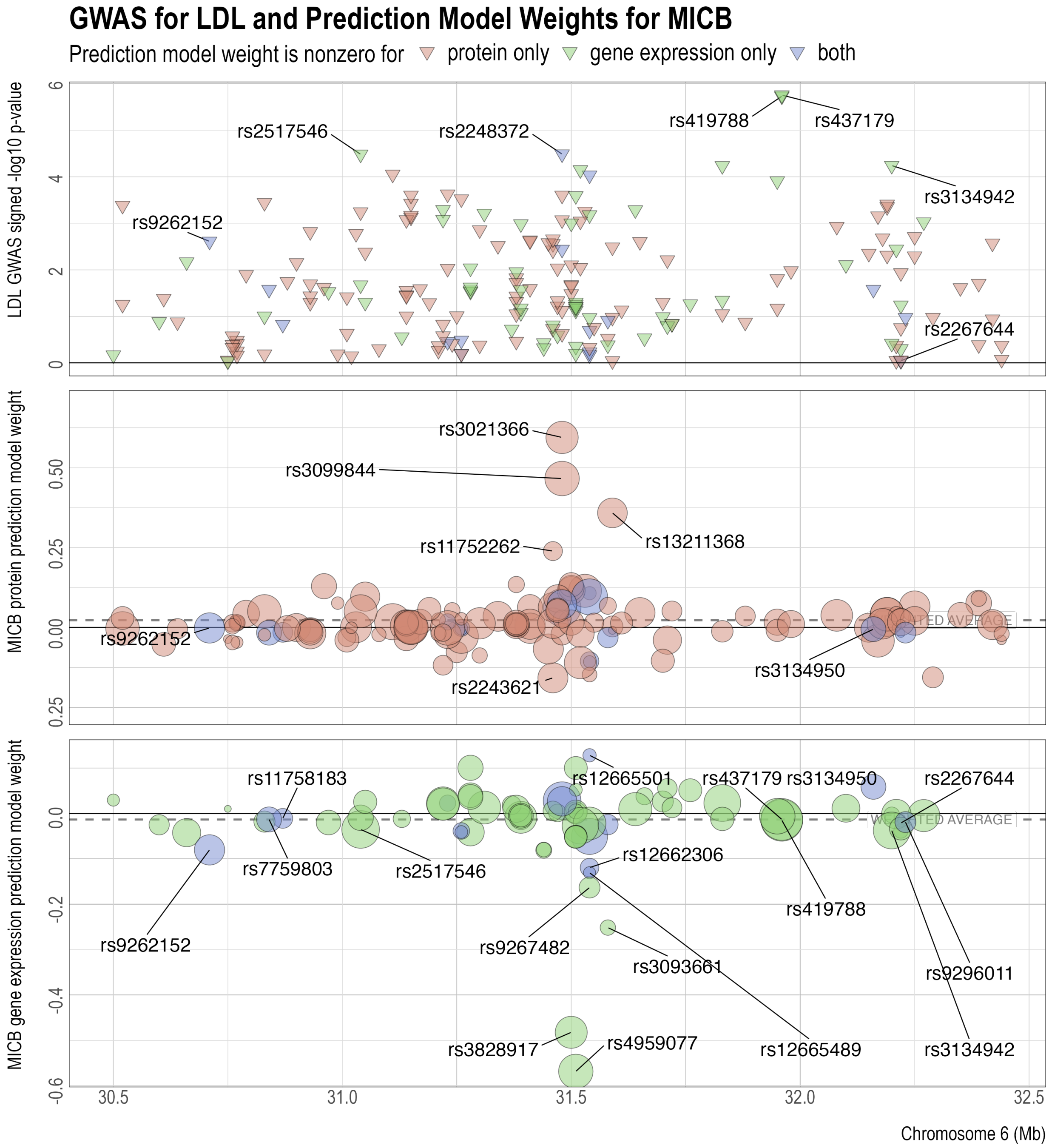

Figure S8: Comparison of MICB's protein and gene expression predictive model weights with the LDL GWAS z-scores of the SNPs. The reference and alternative alleles for GWAS and the predictive models have been aligned and reordered so that all the SNPs have positive GWAS effects. The z-scores are used to compute the weighted average of the model weights (dashed lines), which have the same signs as and are proportional to the predicted effects of protein and gene expression on the GWAS outcome.

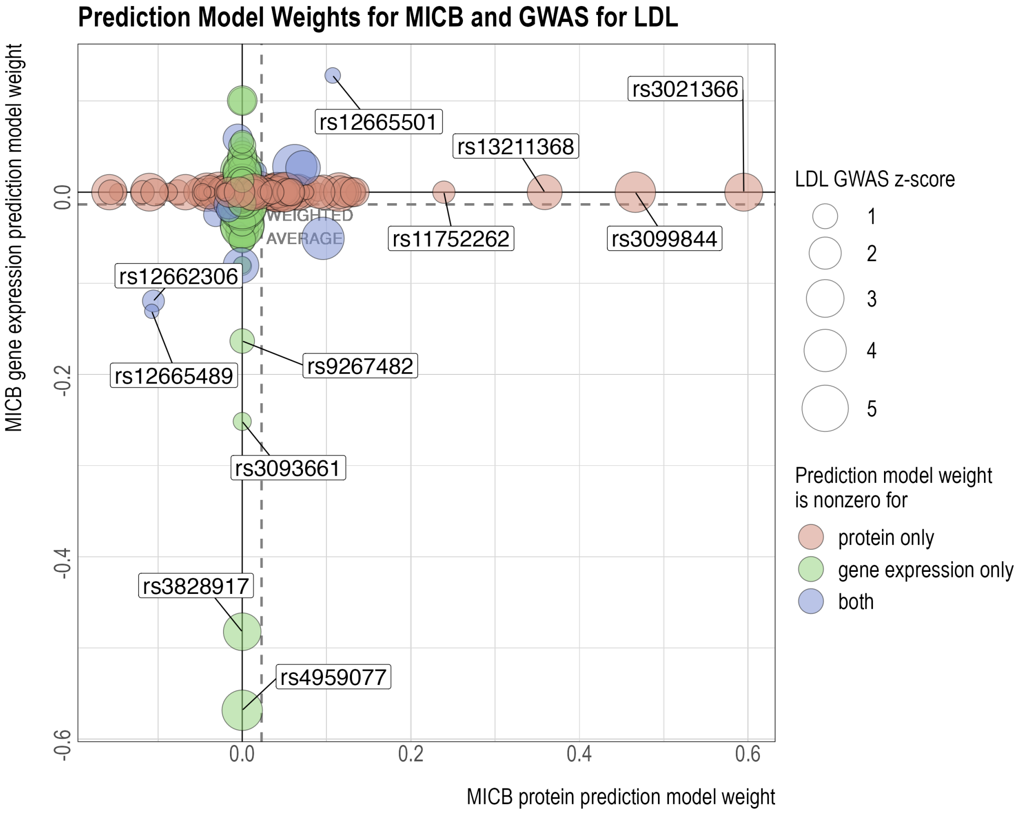

Figure S9: GWAS for TC and prediction models for APOE's protein and gene expression levels. The reference and alternative alleles for GWAS and the predictive models have been aligned and reordered so that all the SNPs have positive GWAS effects. In the center and bottom panels, the size of the circles indicates the SNP's GWAS z-score. The z-scores are used to compute the weighted average of the model weights (dashed line), which has the same sign as and is proportional to the predicted effect of protein or gene expression on the GWAS outcome.

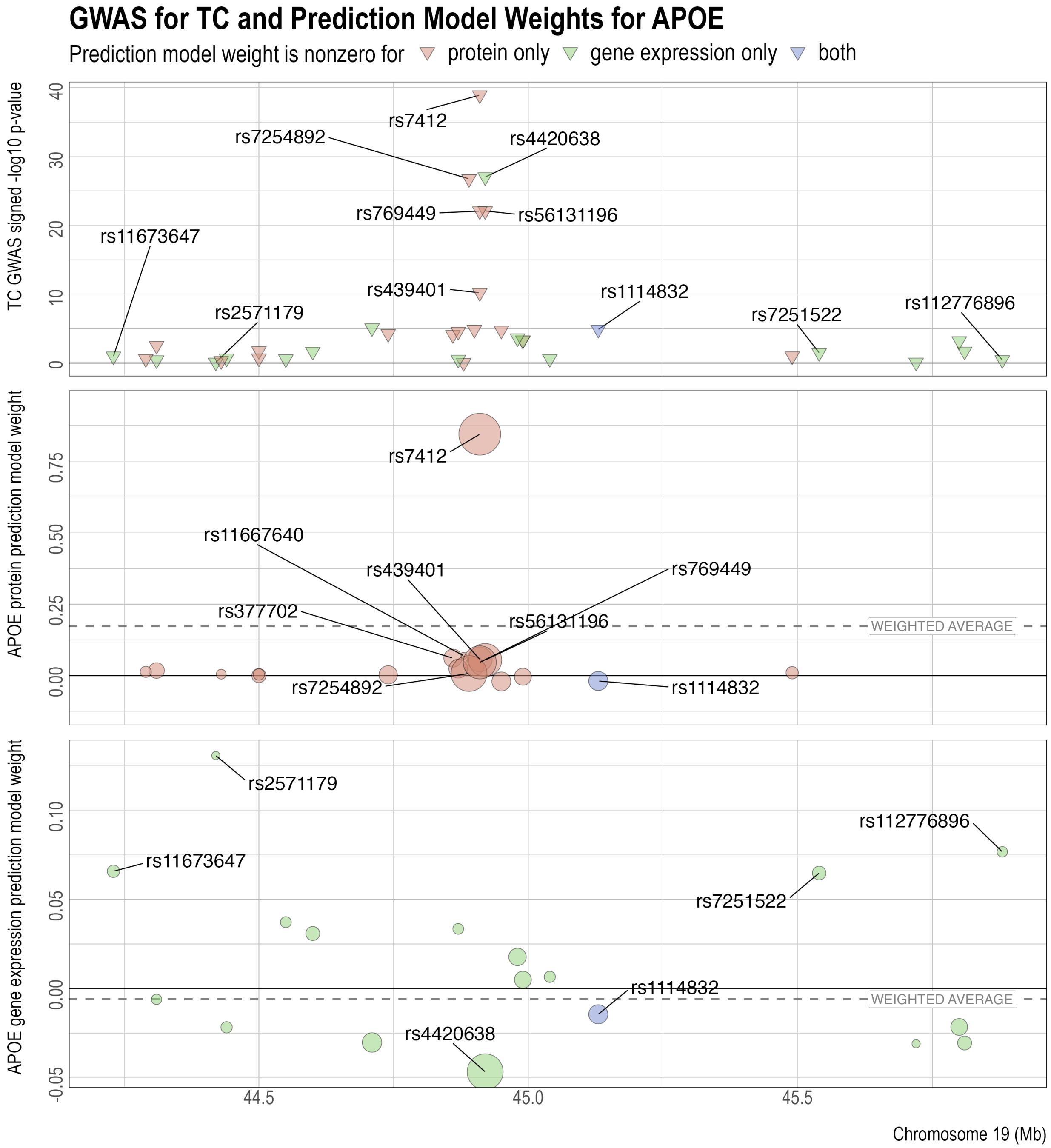

Figure S10: Comparison of APOE's protein and gene expression predictive model weights with the TC GWAS z-scores of the SNPs. The reference and alternative alleles for GWAS and the predictive models have been aligned and reordered so that all the SNPs have positive GWAS effects. The z-scores are used to compute the weighted average of the model weights (dashed lines), which have the same signs as and are proportional to the predicted effects of protein and gene expression on the GWAS outcome.

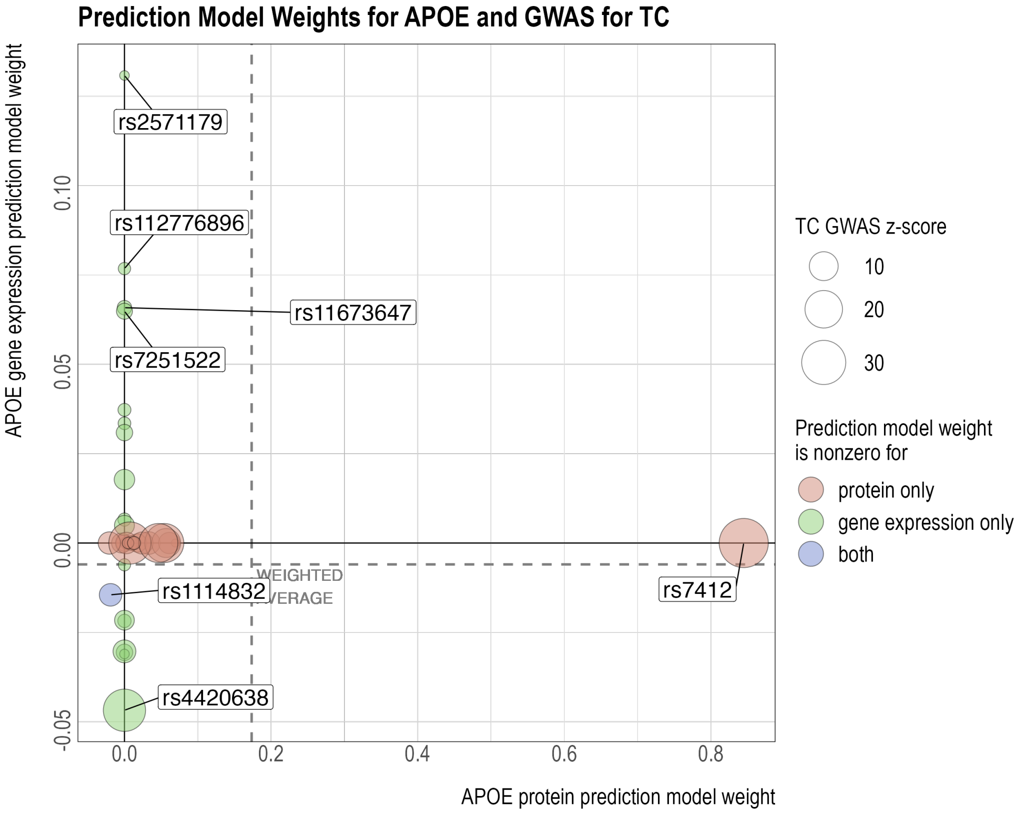

Figure S11: GWAS for TC and prediction models for FCGR2B's protein and gene expression levels. The reference and alternative alleles for GWAS and the predictive models have been aligned and reordered so that all the SNPs have positive GWAS effects. In the center and bottom panels, the size of the circles indicates the SNP's GWAS z-score. The z-scores are used to compute the weighted average of the model weights (dashed line), which has the same sign as and is proportional to the predicted effect of protein or gene expression on the GWAS outcome.

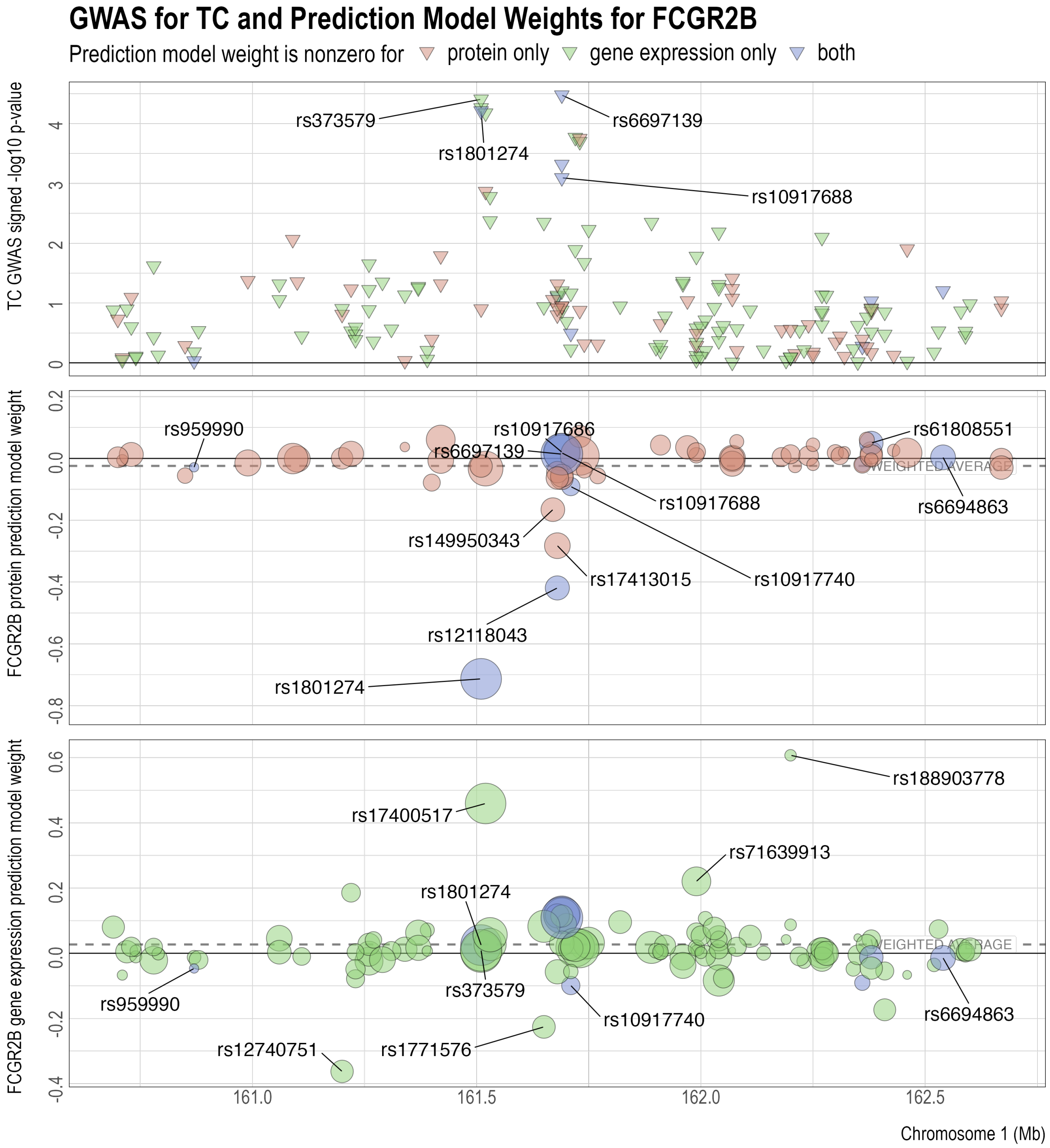

Figure S12: Comparison of FCGR2B's protein and gene expression predictive model weights with the TC GWAS z-scores of the SNPs. The reference and alternative alleles for GWAS and the predictive models have been aligned and reordered so that all the SNPs have positive GWAS effects. The z-scores are used to compute the weighted average of the model weights (dashed lines), which have the same signs as and are proportional to the predicted effects of protein and gene expression on the GWAS outcome.

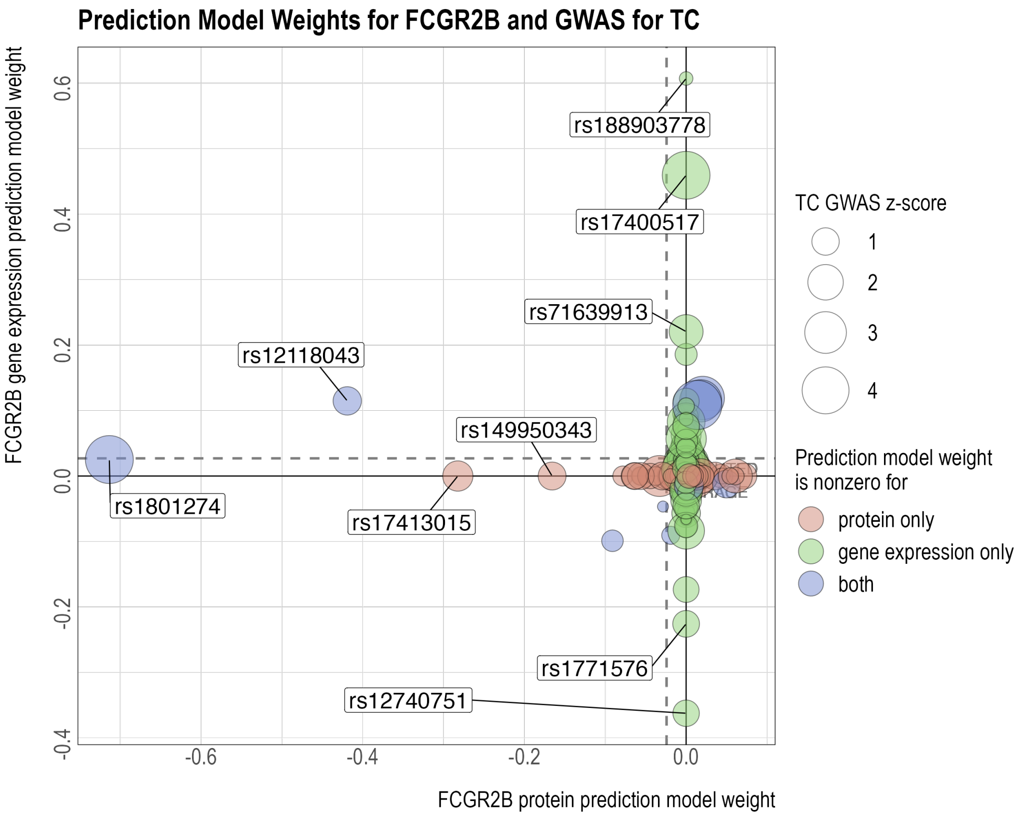

Figure S13: GWAS for TC and prediction models for LILRB2's protein and gene expression levels. The reference and alternative alleles for GWAS and the predictive models have been aligned and reordered so that all the SNPs have positive GWAS effects. In the center and bottom panels, the size of the circles indicates the SNP's GWAS z-score. The z-scores are used to compute the weighted average of the model weights (dashed line), which has the same sign as and is proportional to the predicted effect of protein or gene expression on the GWAS outcome.

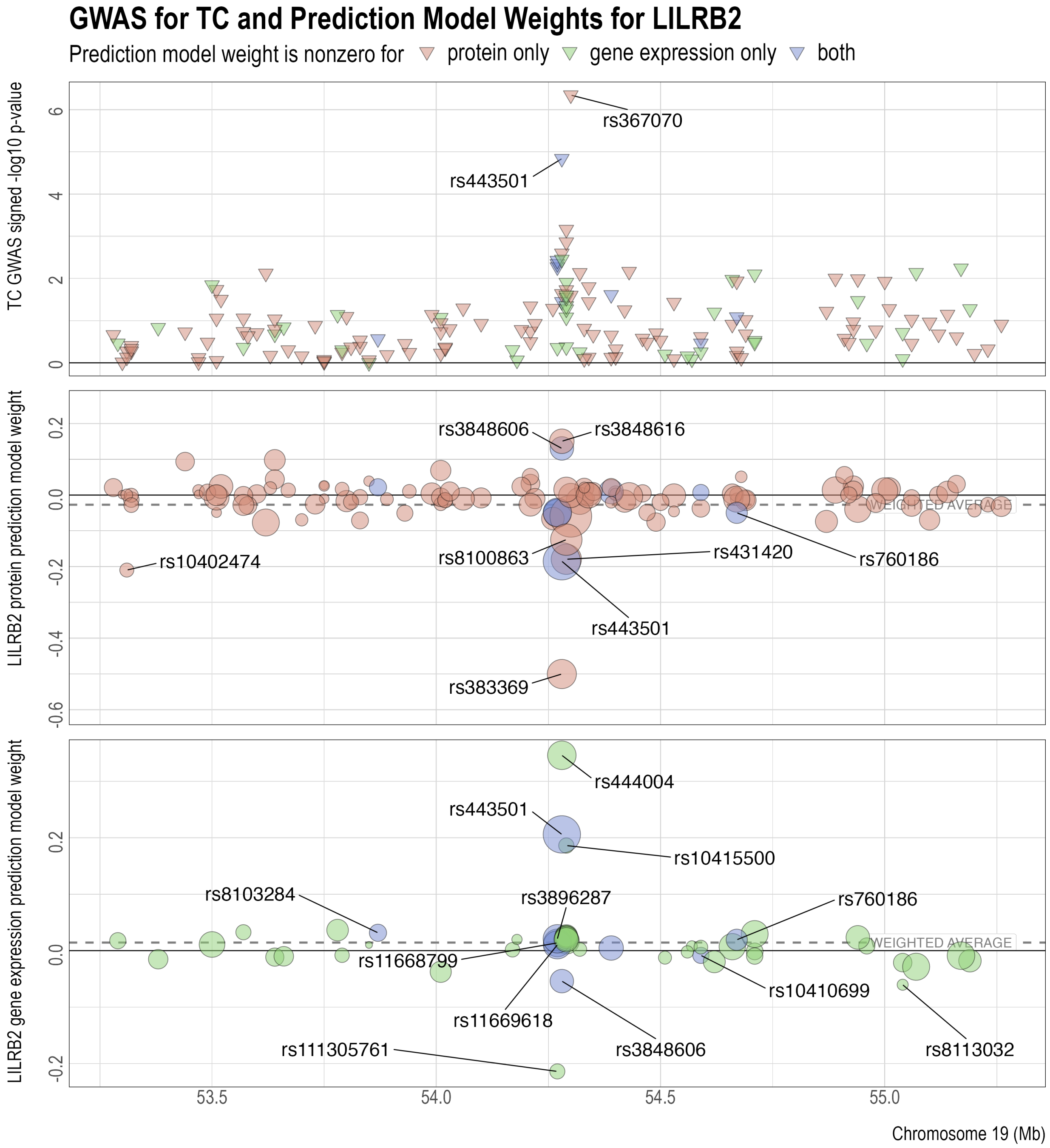

Figure S14: Comparison of LILRB2's protein and gene expression predictive model weights with the TC GWAS z-scores of the SNPs. The reference and alternative alleles for GWAS and the predictive models have been aligned and reordered so that all the SNPs have positive GWAS effects. The z-scores are used to compute the weighted average of the model weights (dashed lines), which have the same signs as and are proportional to the predicted effects of protein and gene expression on the GWAS outcome.

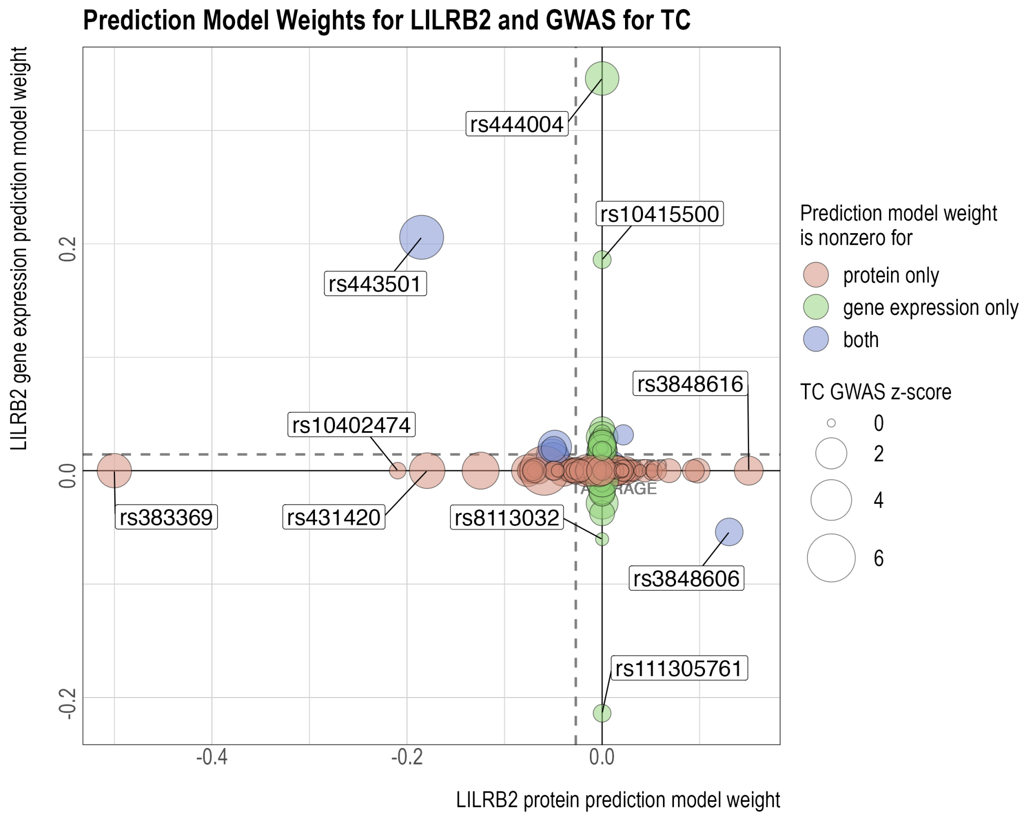

Figure S15: GWAS for TC and prediction models for MICB's protein and gene expression levels. The reference and alternative alleles for GWAS and the predictive models have been aligned and reordered so that all the SNPs have positive GWAS effects. In the center and bottom panels, the size of the circles indicates the SNP's GWAS z-score. The z-scores are used to compute the weighted average of the model weights (dashed line), which has the same sign as and is proportional to the predicted effect of protein or gene expression on the GWAS outcome.

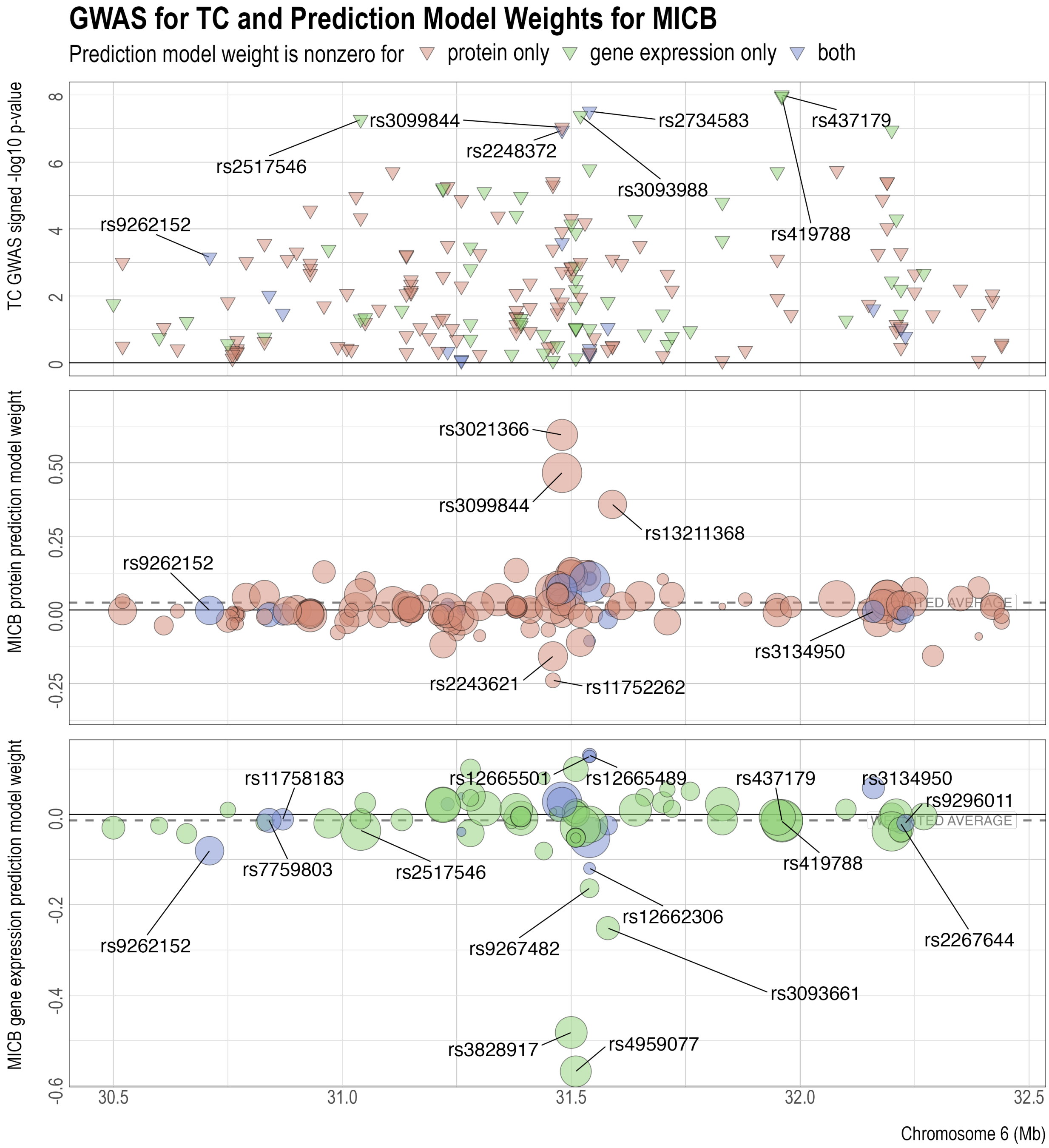

Figure S16: Comparison of MICB's protein and gene expression predictive model weights with the TC GWAS z-scores of the SNPs. The reference and alternative alleles for GWAS and the predictive models have been aligned and reordered so that all the SNPs have positive GWAS effects. The z-scores are used to compute the weighted average of the model weights (dashed lines), which have the same signs as and are proportional to the predicted effects of protein and gene expression on the GWAS outcome.

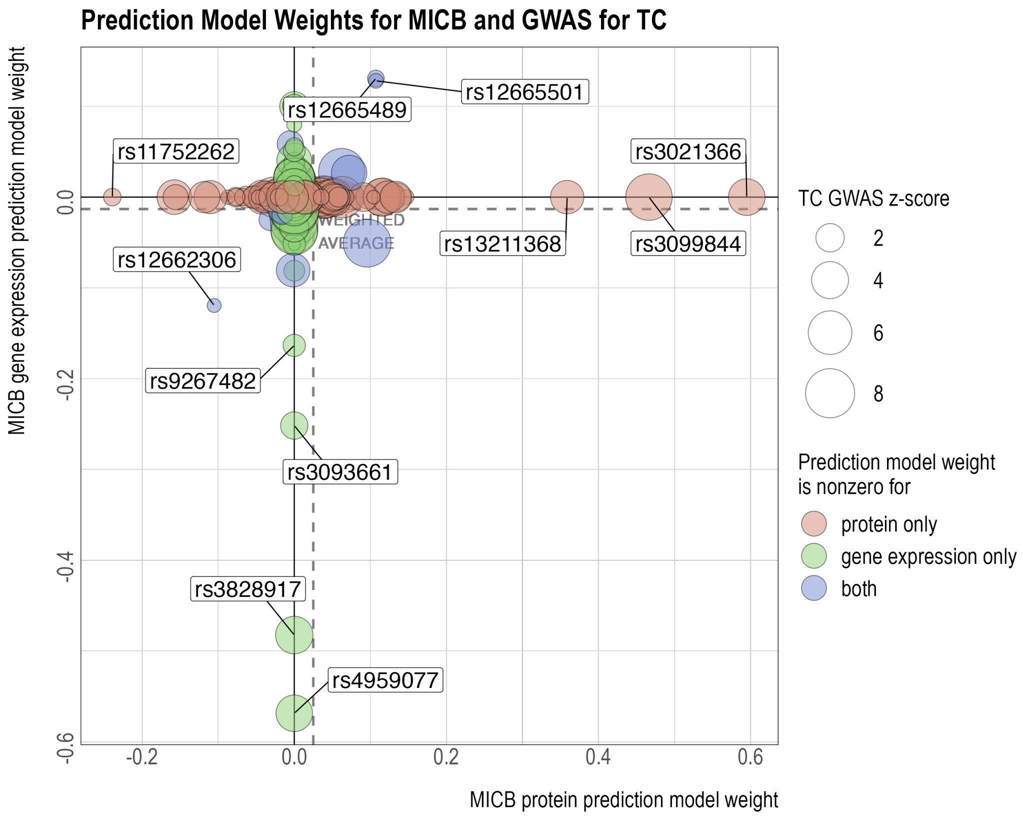

Figure S17: Comparison of MESA whole blood PWAS, MESA PBMC TWAS, and GTEx tissue-specific TWAS results for TC. Panel (a): signed log p-value and significance of association. Missing values are shown in white. Significance of association is deteremined by the false discovery rate (FDR) threshold of 0.05. Panel (b): correlation between signed log p-values of MESA whole blood PWAS and signed log p-values of each GTEx tissue-specific TWAS (i.e. the correlation between the bottom row and every other row of the grid in Panel (a)).

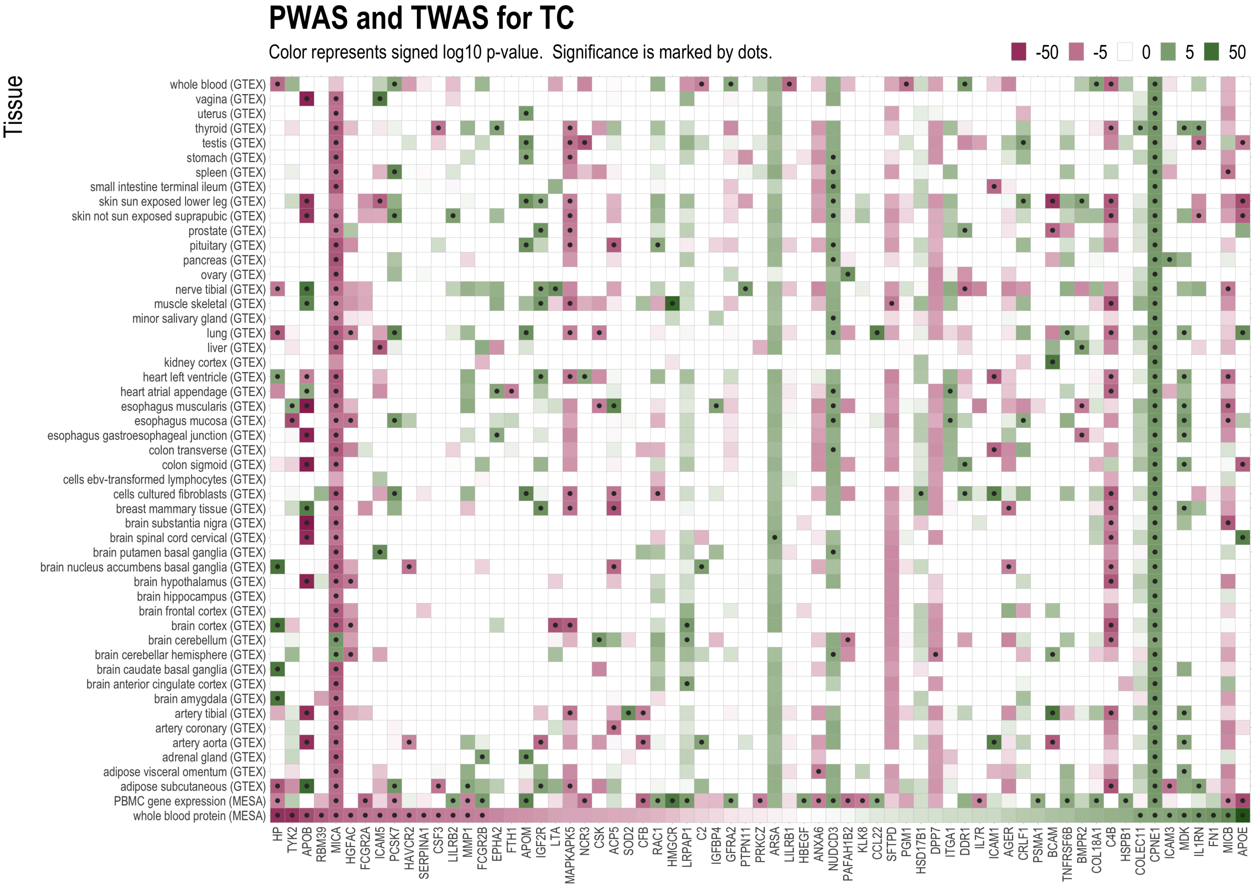

(a)

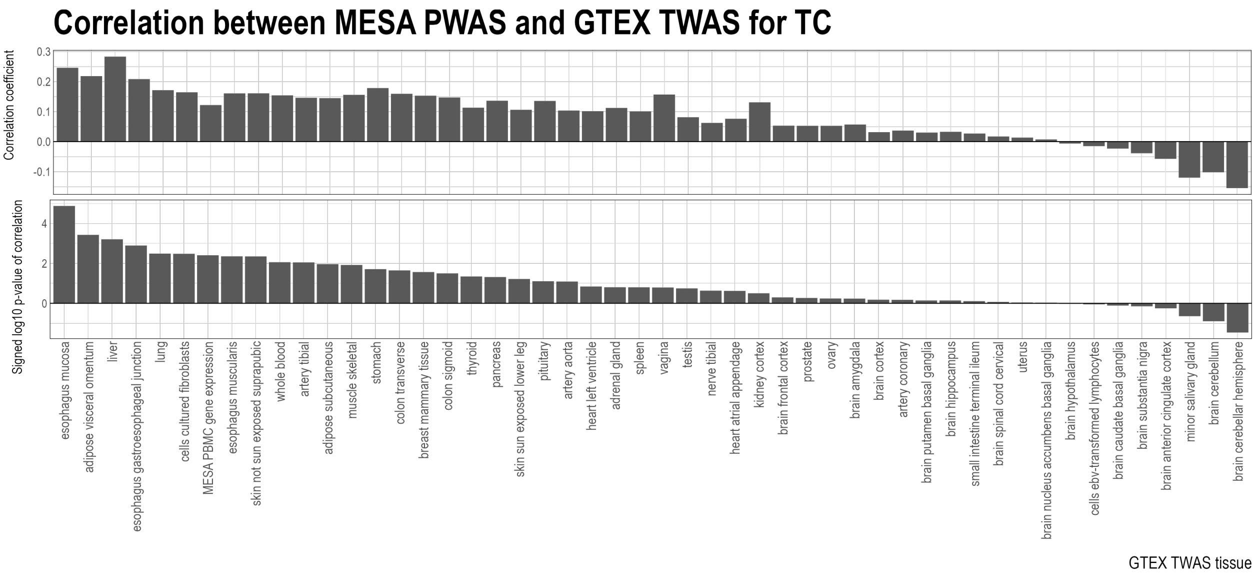

(b)

Figure S18: GWAS for TG and prediction models for APOE's protein and gene expression levels. The reference and alternative alleles for GWAS and the predictive models have been aligned and reordered so that all the SNPs have positive GWAS effects. In the center and bottom panels, the size of the circles indicates the SNP's GWAS z-score. The z-scores are used to compute the weighted average of the model weights (dashed line), which has the same sign as and is proportional to the predicted effect of protein or gene expression on the GWAS outcome.

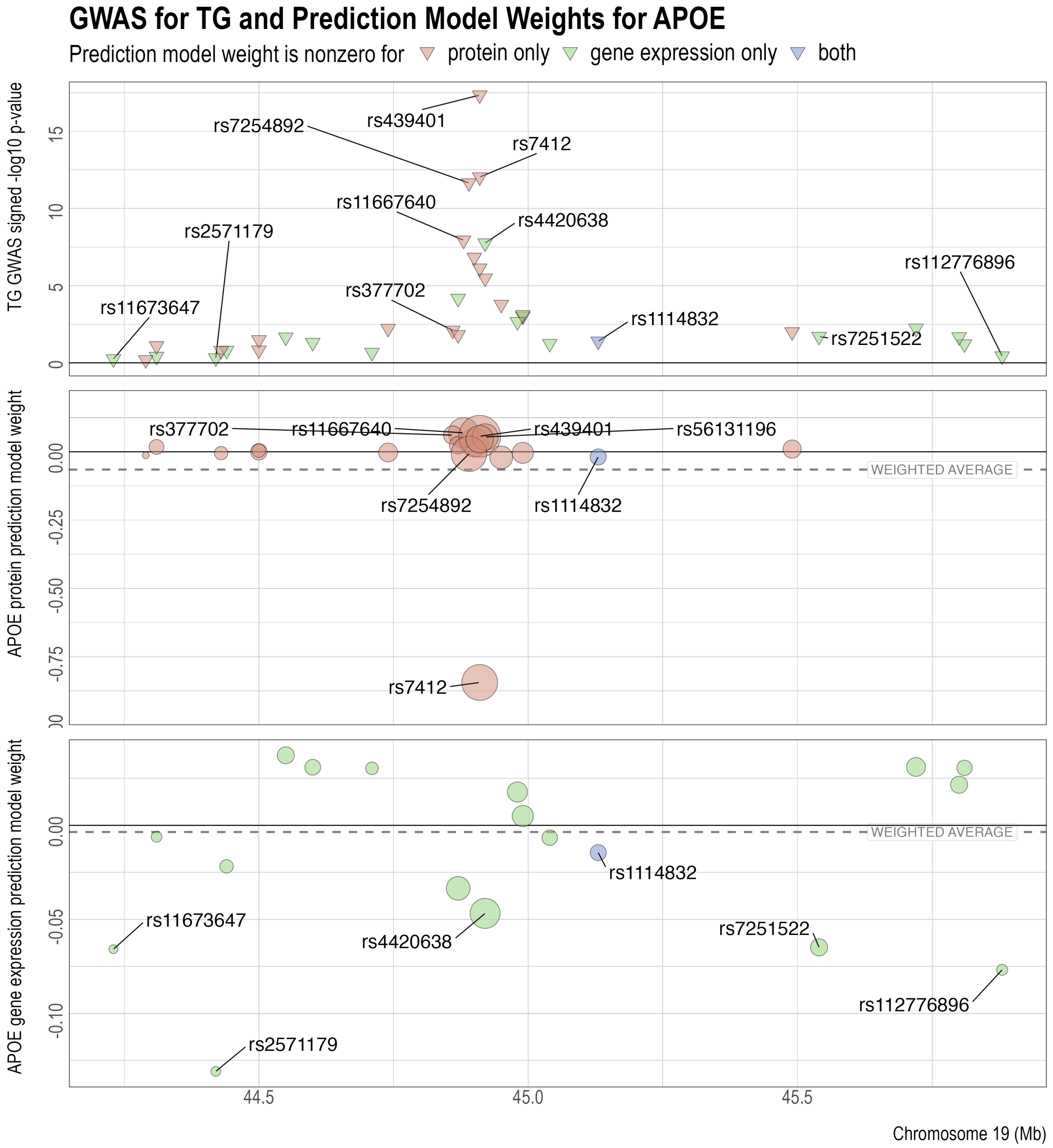

Figure S19: Comparison of APOE's protein and gene expression predictive model weights with the TG GWAS z-scores of the SNPs. The reference and alternative alleles for GWAS and the predictive models have been aligned and reordered so that all the SNPs have positive GWAS effects. The z-scores are used to compute the weighted average of the model weights (dashed lines), which have the same signs as and are proportional to the predicted effects of protein and gene expression on the GWAS outcome.

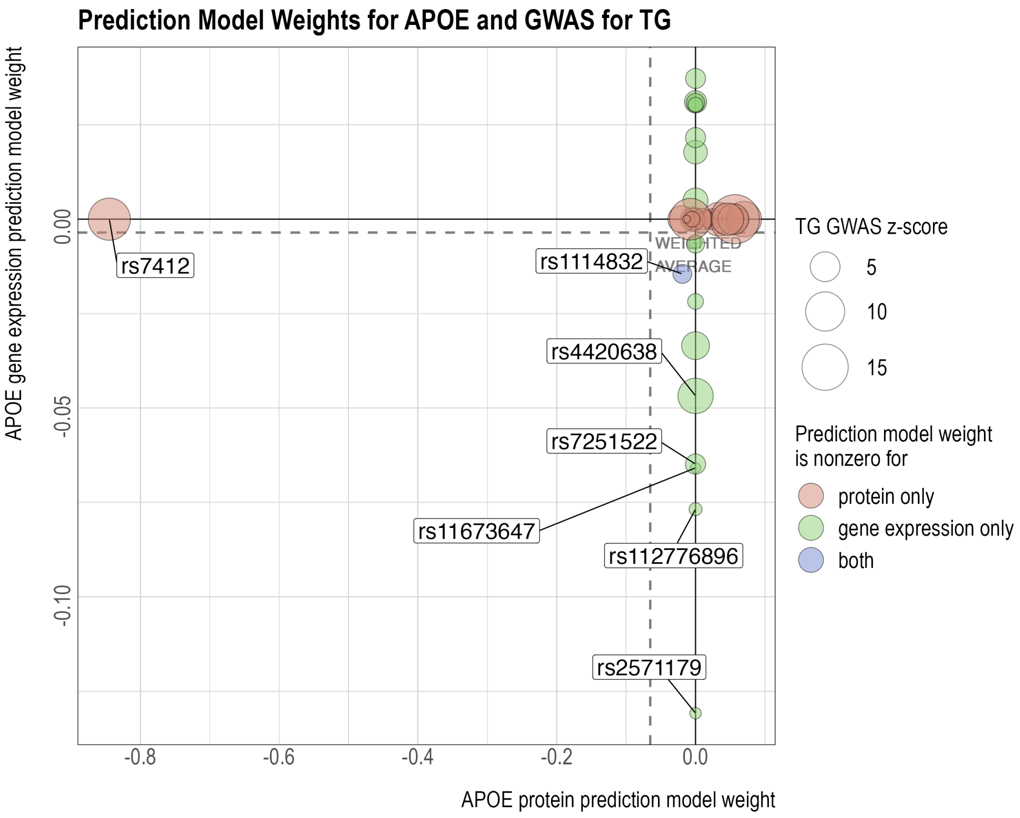

Figure S20: GWAS for TG and prediction models for FCGR2B's protein and gene expression levels. The reference and alternative alleles for GWAS and the predictive models have been aligned and reordered so that all the SNPs have positive GWAS effects. In the center and bottom panels, the size of the circles indicates the SNP's GWAS z-score. The z-scores are used to compute the weighted average of the model weights (dashed line), which has the same sign as and is proportional to the predicted effect of protein or gene expression on the GWAS outcome.

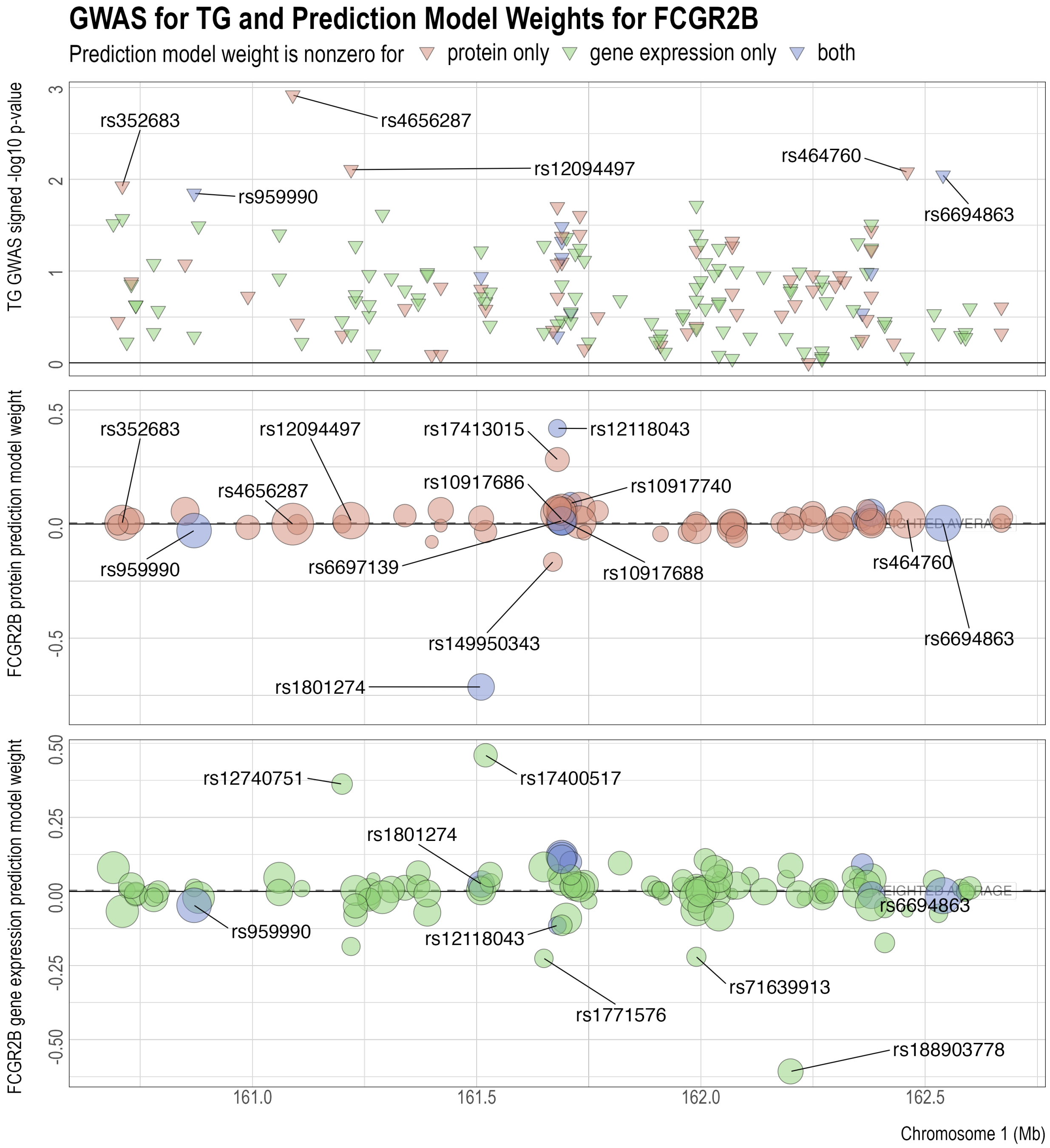

Figure S21: Comparison of FCGR2B's protein and gene expression predictive model weights with the TG GWAS z-scores of the SNPs. The reference and alternative alleles for GWAS and the predictive models have been aligned and reordered so that all the SNPs have positive GWAS effects. The z-scores are used to compute the weighted average of the model weights (dashed lines), which have the same signs as and are proportional to the predicted effects of protein and gene expression on the GWAS outcome.

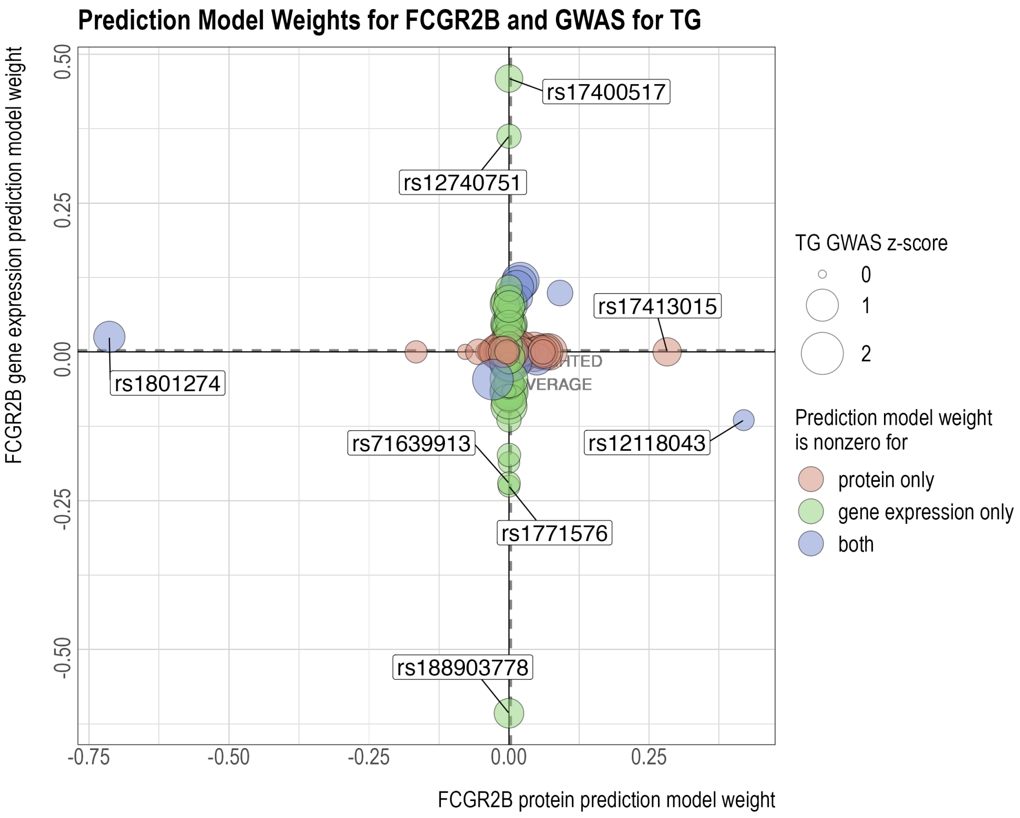

Figure S22: GWAS for TG and prediction models for LILRB2's protein and gene expression levels. The reference and alternative alleles for GWAS and the predictive models have been aligned and reordered so that all the SNPs have positive GWAS effects. In the center and bottom panels, the size of the circles indicates the SNP's GWAS z-score. The z-scores are used to compute the weighted average of the model weights (dashed line), which has the same sign as and is proportional to the predicted effect of protein or gene expression on the GWAS outcome.

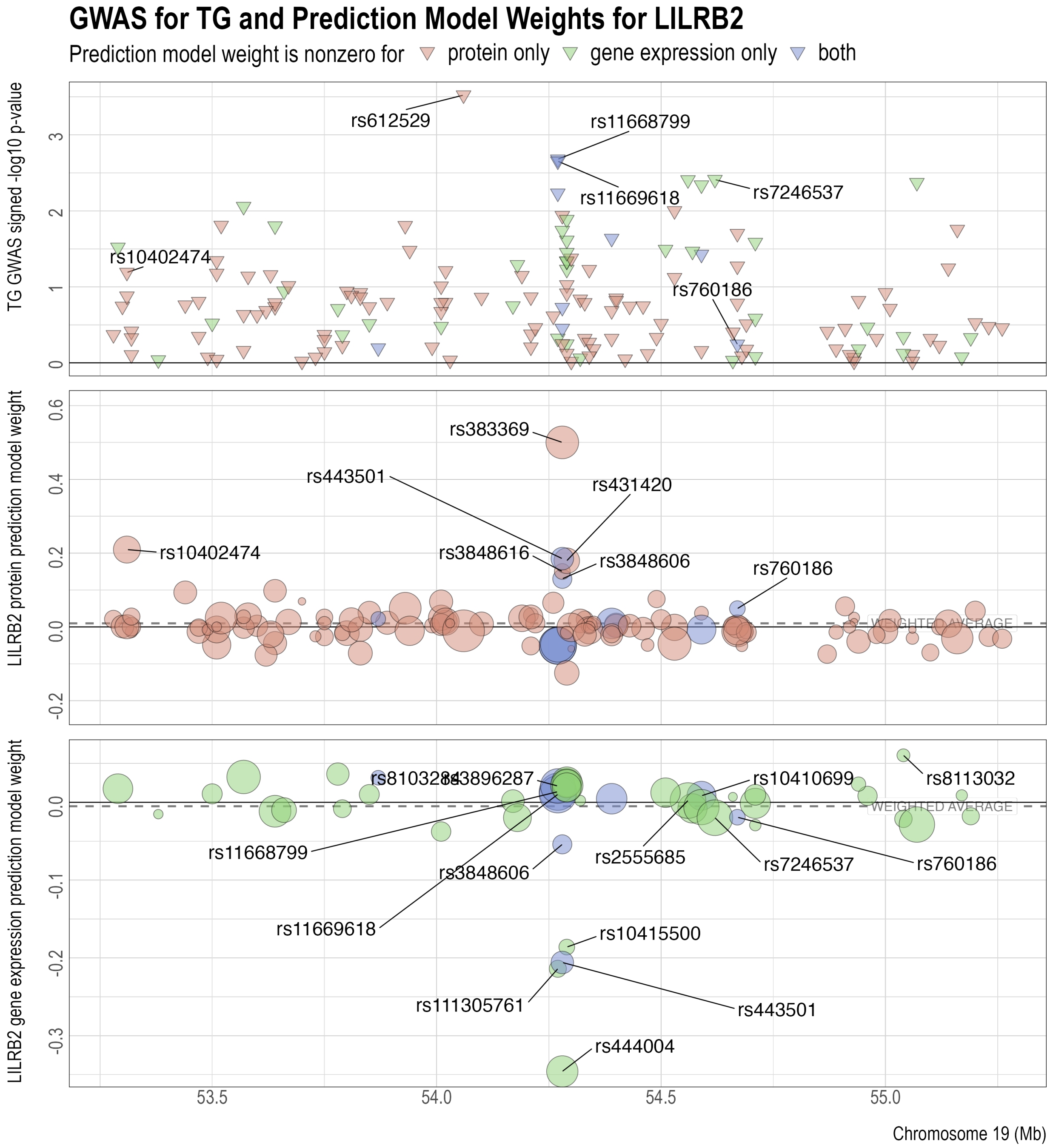

Figure S23: Comparison of LILRB2's protein and gene expression predictive model weights with the TG GWAS z-scores of the SNPs. The reference and alternative alleles for GWAS and the predictive models have been aligned and reordered so that all the SNPs have positive GWAS effects. The z-scores are used to compute the weighted average of the model weights (dashed lines), which have the same signs as and are proportional to the predicted effects of protein and gene expression on the GWAS outcome.

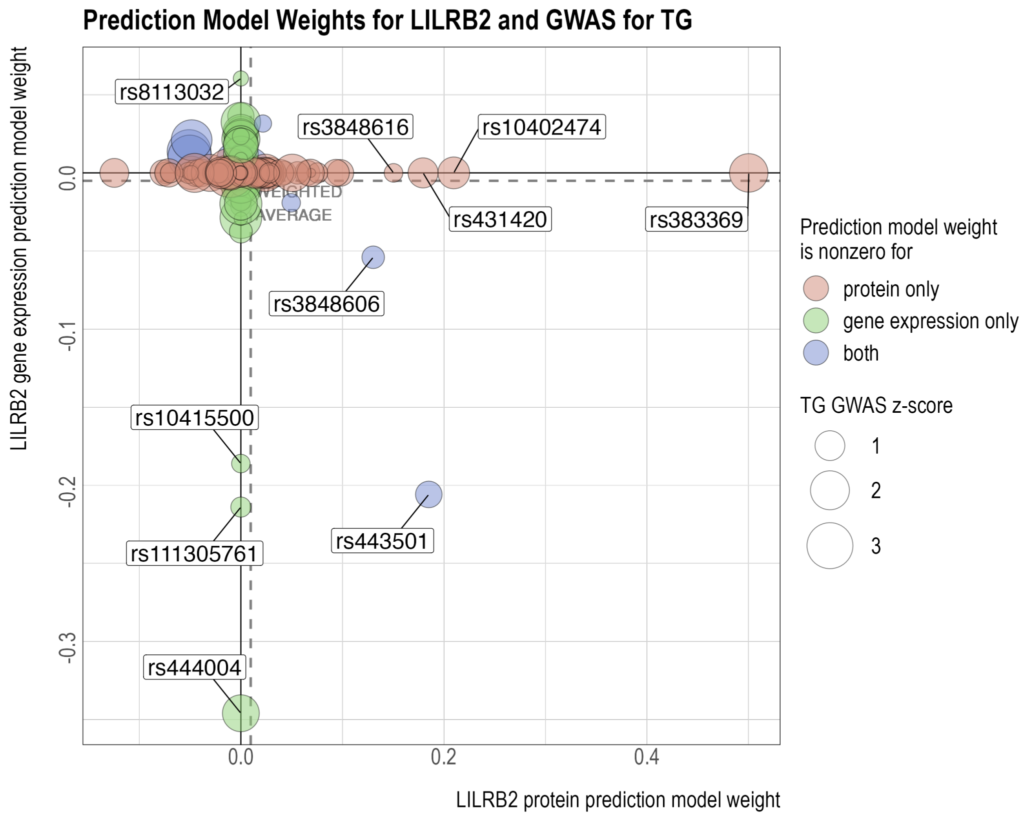

Figure S24: GWAS for TG and prediction models for MICB's protein and gene expression levels. The reference and alternative alleles for GWAS and the predictive models have been aligned and reordered so that all the SNPs have positive GWAS effects. In the center and bottom panels, the size of the circles indicates the SNP's GWAS z-score. The z-scores are used to compute the weighted average of the model weights (dashed line), which has the same sign as and is proportional to the predicted effect of protein or gene expression on the GWAS outcome.

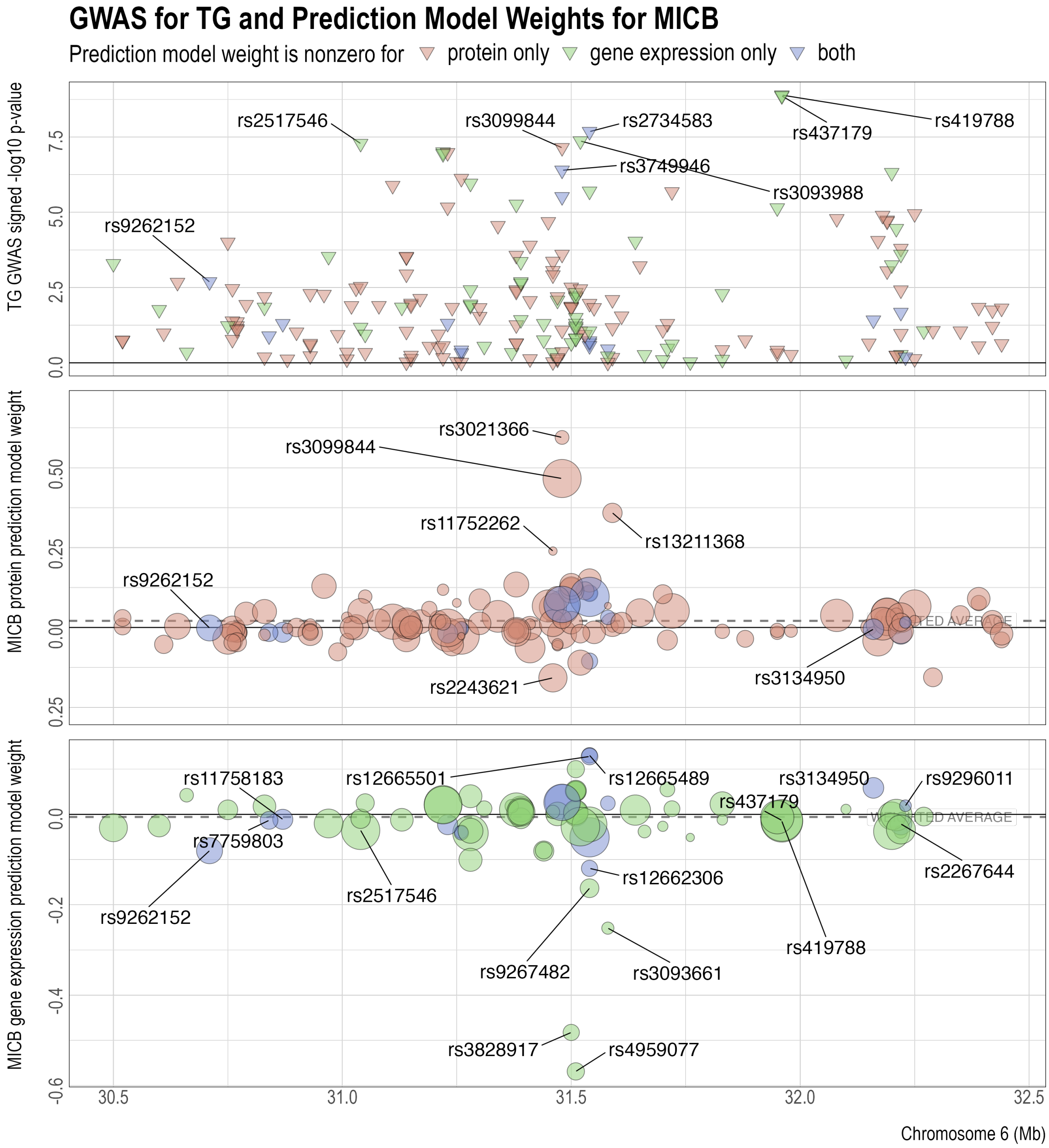

Figure S25: Comparison of MICB's protein and gene expression predictive model weights with the TG GWAS z-scores of the SNPs. The reference and alternative alleles for GWAS and the predictive models have been aligned and reordered so that all the SNPs have positive GWAS effects. The z-scores are used to compute the weighted average of the model weights (dashed lines), which have the same signs as and are proportional to the predicted effects of protein and gene expression on the GWAS outcome.

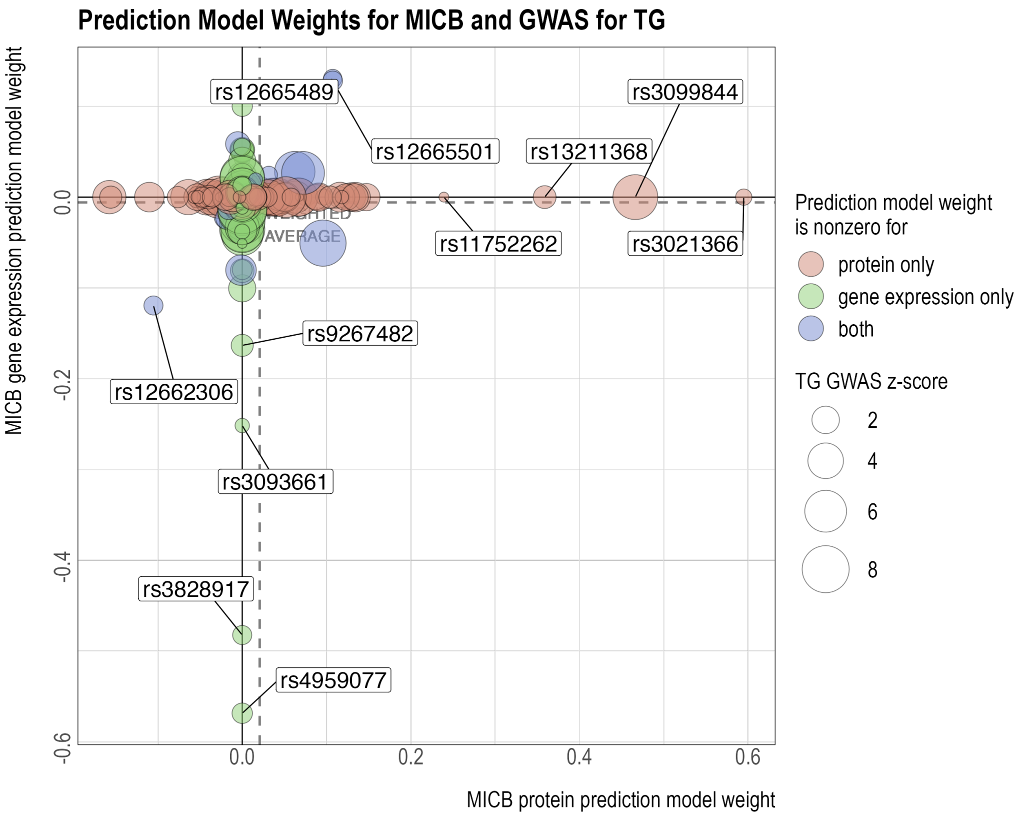

Figure S26: Comparison of MESA whole blood PWAS, MESA PBMC TWAS, and GTEx tissue-specific TWAS results for TG. Panel (a): signed log p-value and significance of association. Missing values are shown in white. Significance of association is deteremined by the false discovery rate (FDR) threshold of 0.05. Panel (b): correlation between signed log p-values of MESA whole blood PWAS and signed log p-values of each GTEx tissue-specific TWAS (i.e. the correlation between the bottom row and every other row of the grid in Panel (a)).

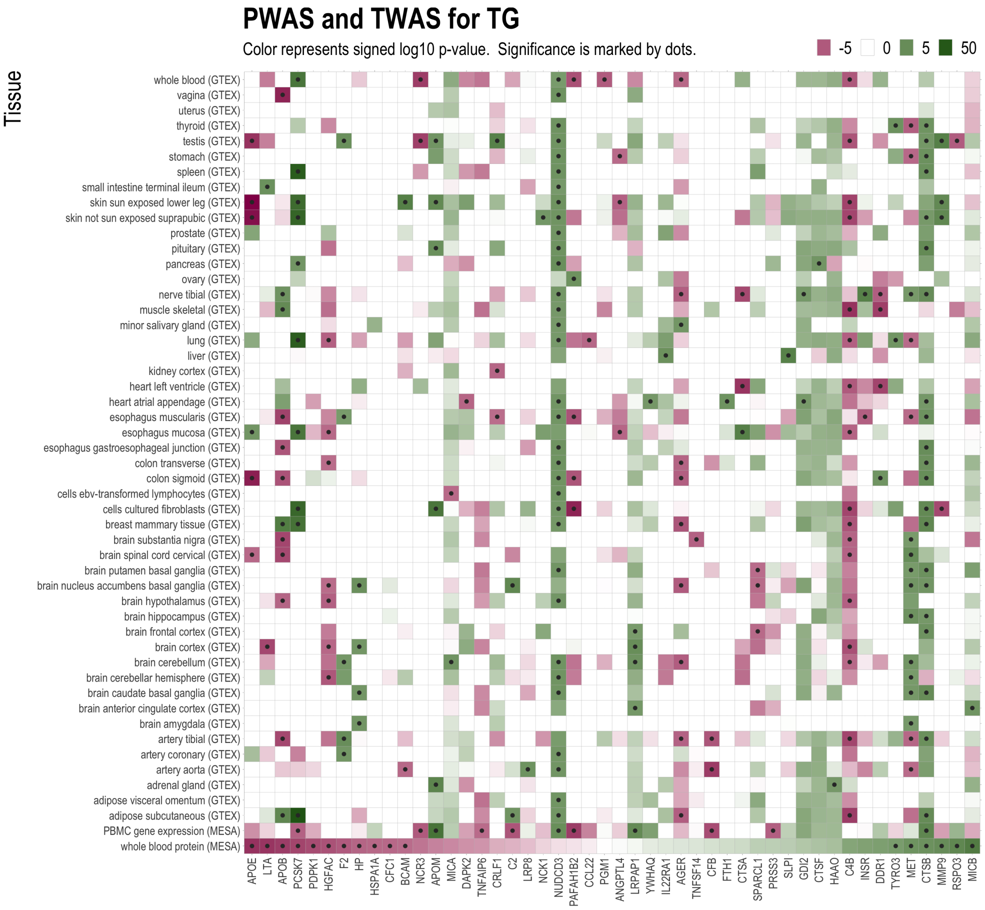

(a)

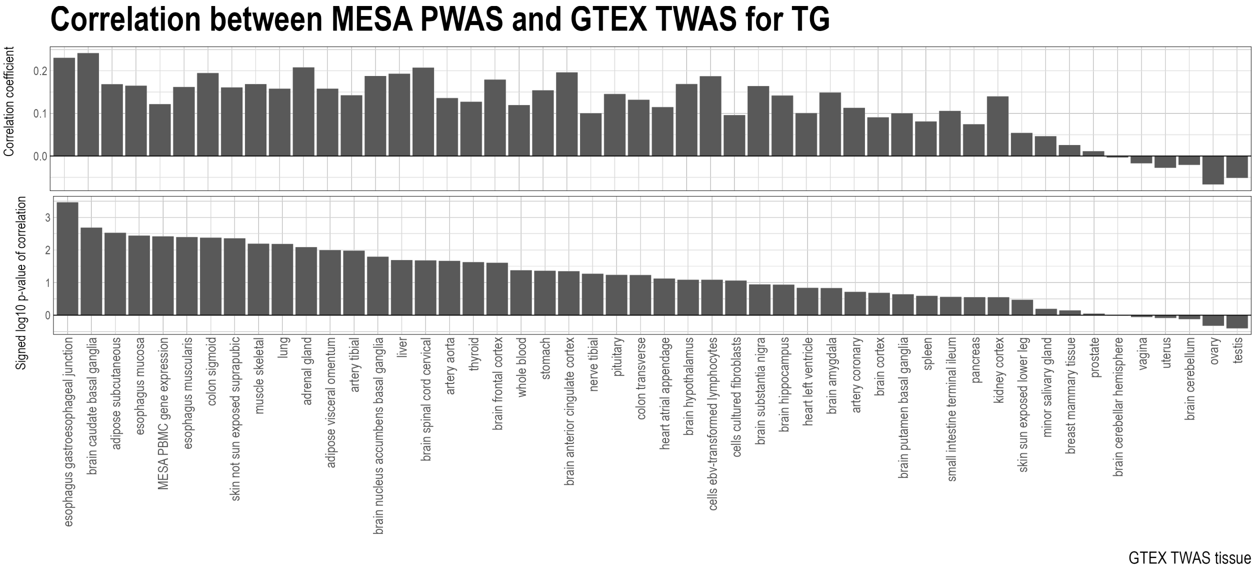

(b)

Figure S27: GWAS for HDL and prediction models for APOE's protein and gene expression levels. The reference and alternative alleles for GWAS and the predictive models have been aligned and reordered so that all the SNPs have positive GWAS effects. In the center and bottom panels, the size of the circles indicates the SNP's GWAS z-score. The z-scores are used to compute the weighted average of the model weights (dashed line), which has the same sign as and is proportional to the predicted effect of protein or gene expression on the GWAS outcome.

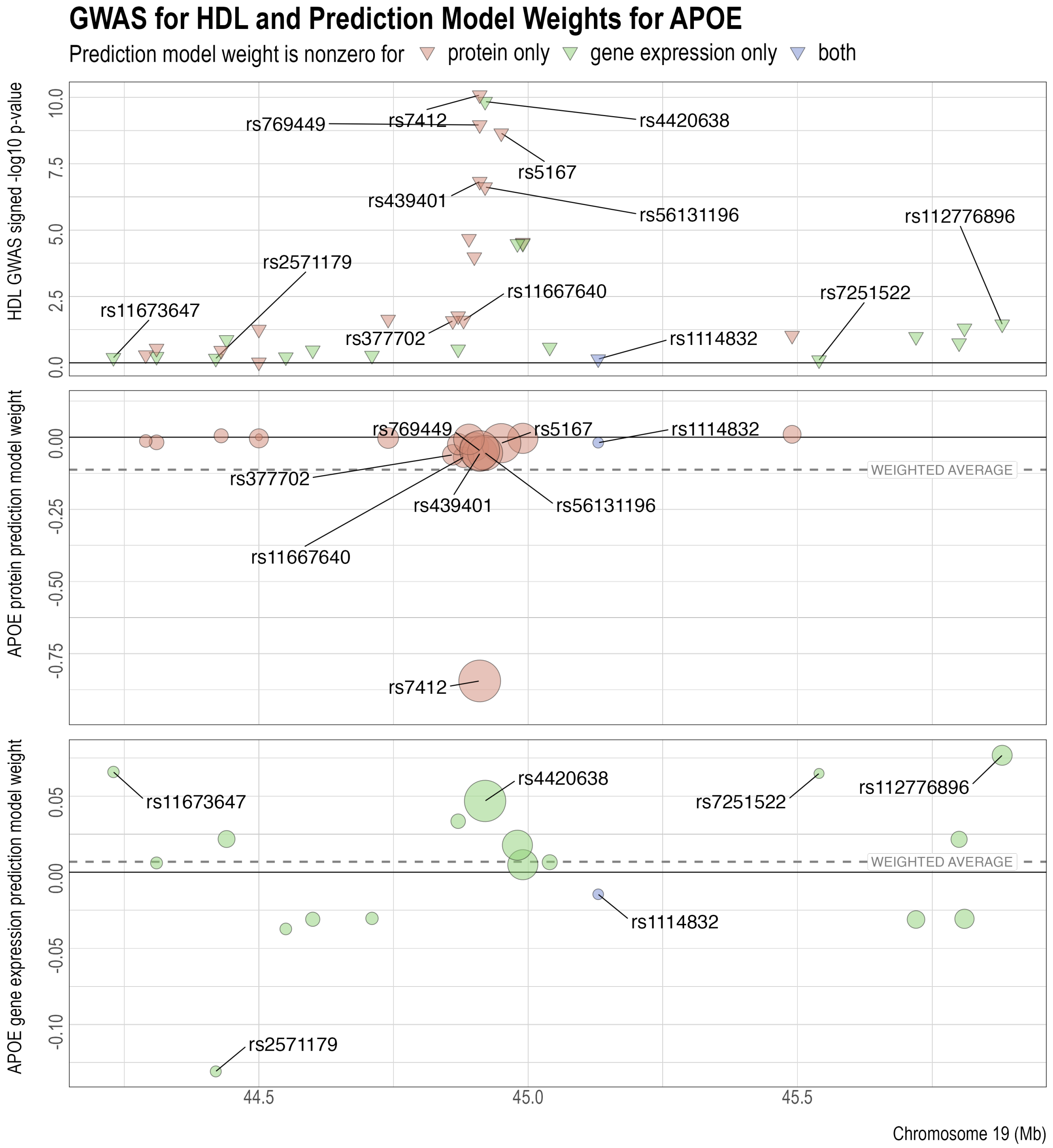

Figure S28: Comparison of APOE's protein and gene expression predictive model weights with the HDL GWAS z-scores of the SNPs. The reference and alternative alleles for GWAS and the predictive models have been aligned and reordered so that all the SNPs have positive GWAS effects. The z-scores are used to compute the weighted average of the model weights (dashed lines), which have the same signs as and are proportional to the predicted effects of protein and gene expression on the GWAS outcome.

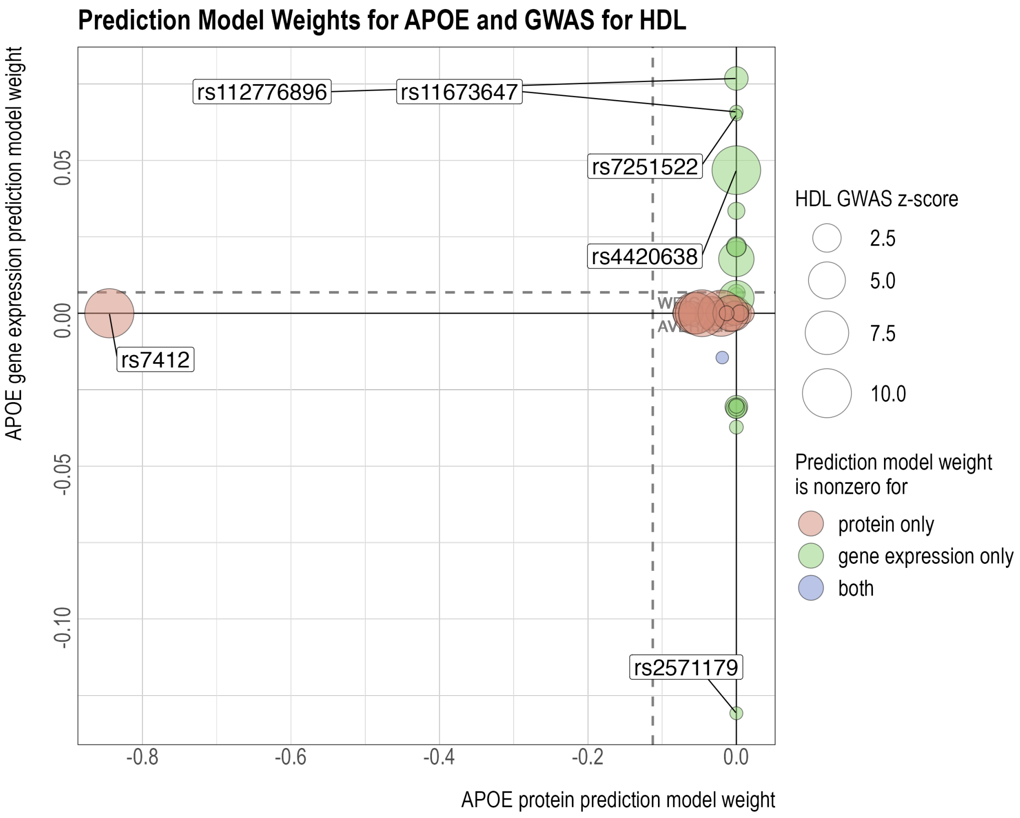

Figure S29: GWAS for HDL and prediction models for FCGR2B's protein and gene expression levels. The reference and alternative alleles for GWAS and the predictive models have been aligned and reordered so that all the SNPs have positive GWAS effects. In the center and bottom panels, the size of the circles indicates the SNP's GWAS z-score. The z-scores are used to compute the weighted average of the model weights (dashed line), which has the same sign as and is proportional to the predicted effect of protein or gene expression on the GWAS outcome.

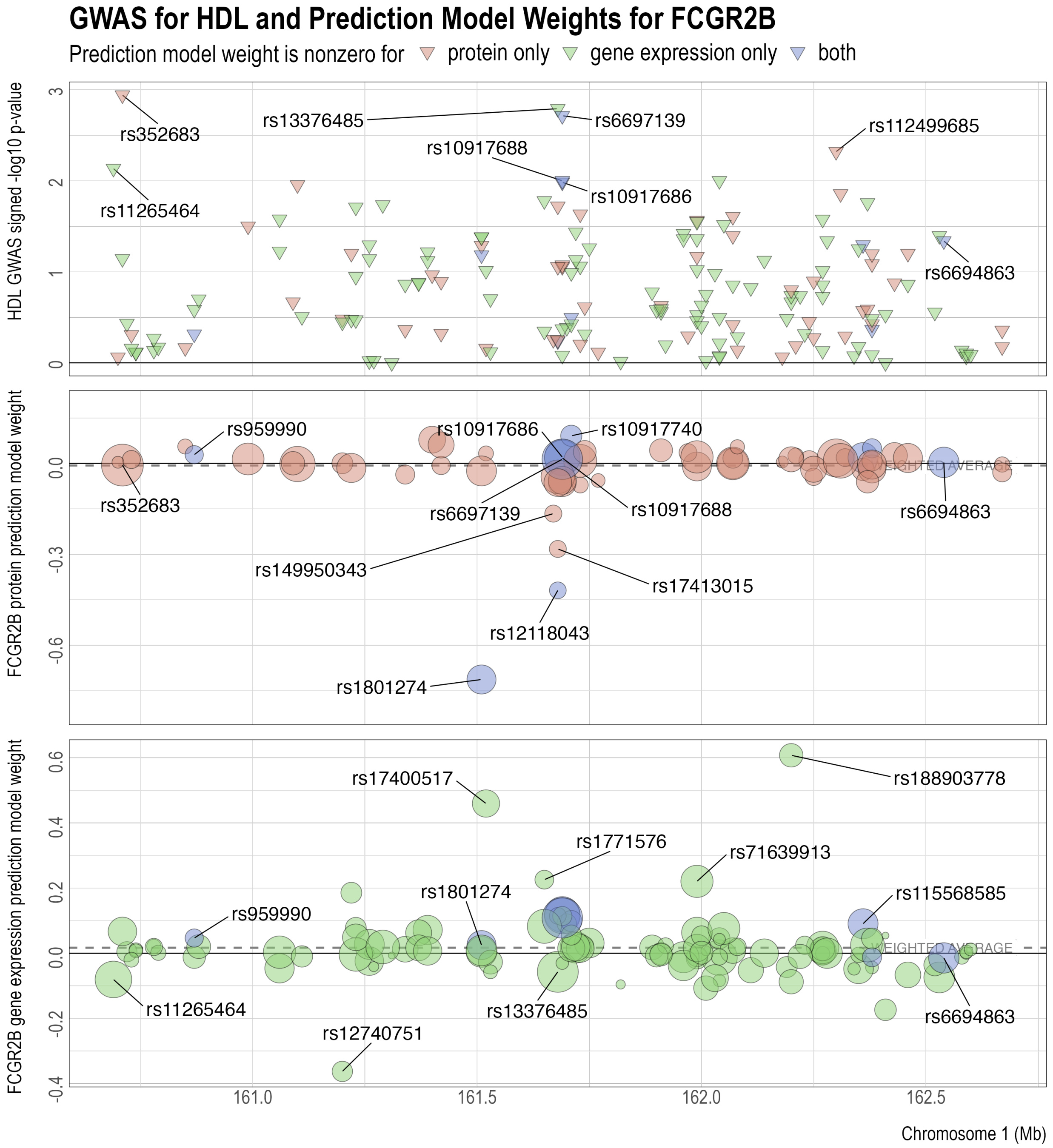

Figure S30: Comparison of FCGR2B's protein and gene expression predictive model weights with the HDL GWAS z-scores of the SNPs. The reference and alternative alleles for GWAS and the predictive models have been aligned and reordered so that all the SNPs have positive GWAS effects. The z-scores are used to compute the weighted average of the model weights (dashed lines), which have the same signs as and are proportional to the predicted effects of protein and gene expression on the GWAS outcome.

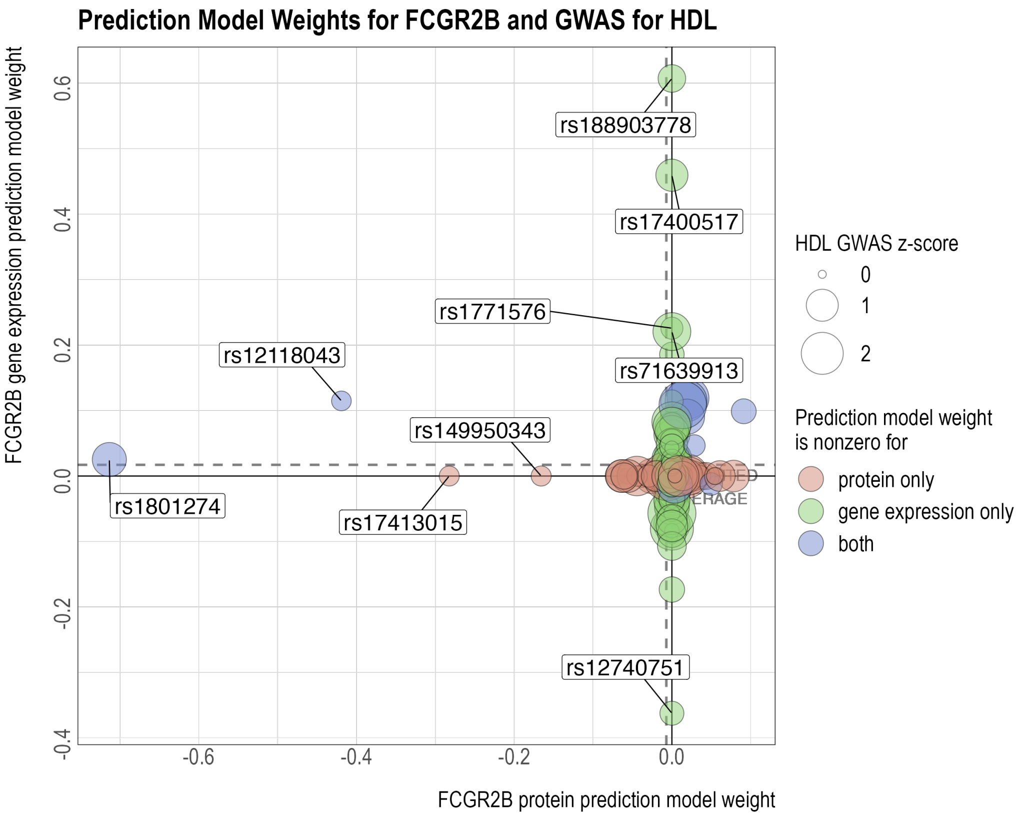

Figure S31: GWAS for HDL and prediction models for LILRB2's protein and gene expression levels. The reference and alternative alleles for GWAS and the predictive models have been aligned and reordered so that all the SNPs have positive GWAS effects. In the center and bottom panels, the size of the circles indicates the SNP's GWAS z-score. The z-scores are used to compute the weighted average of the model weights (dashed line), which has the same sign as and is proportional to the predicted effect of protein or gene expression on the GWAS outcome.

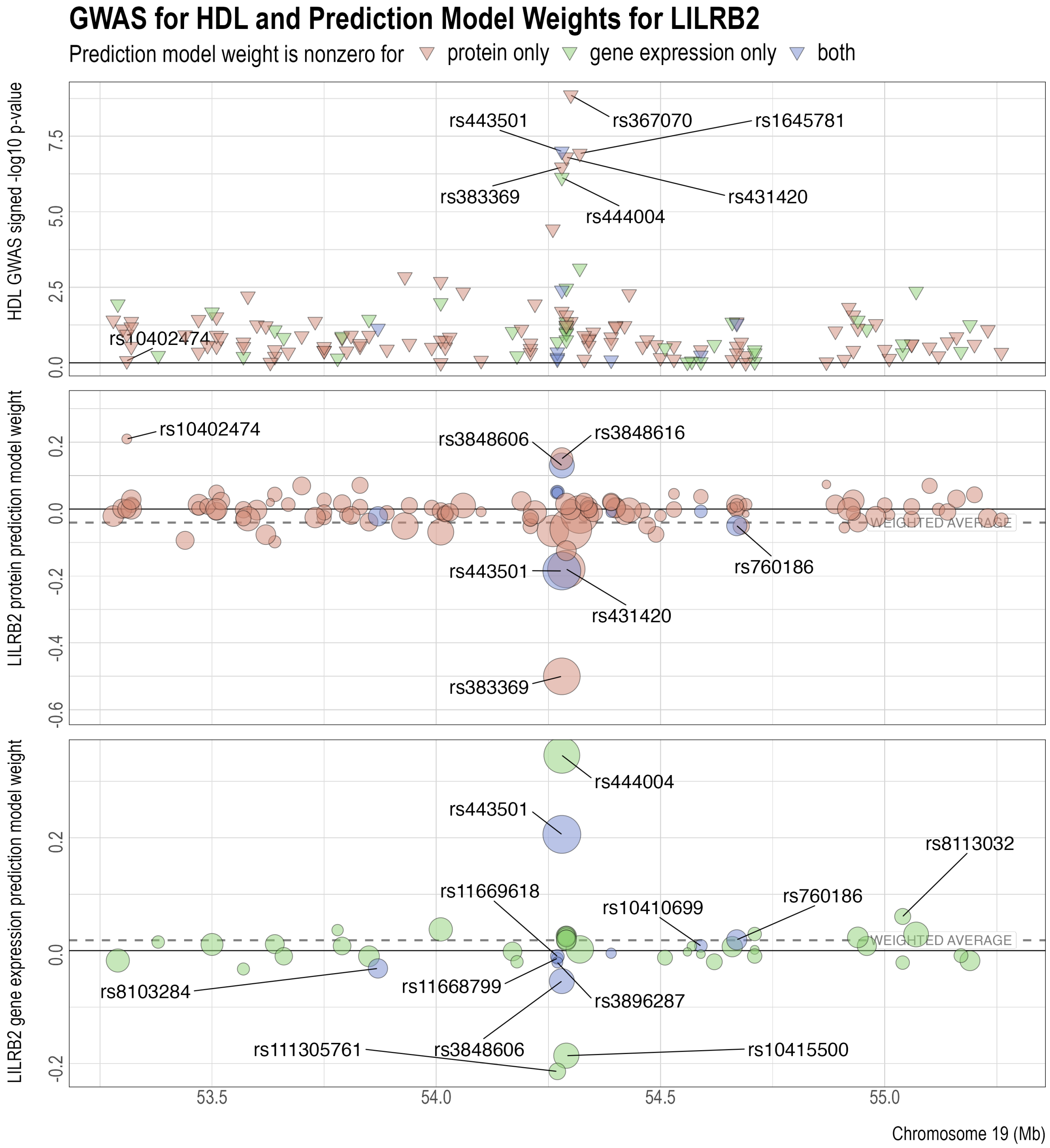

Figure S32: Comparison of LILRB2's protein and gene expression predictive model weights with the HDL GWAS z-scores of the SNPs. The reference and alternative alleles for GWAS and the predictive models have been aligned and reordered so that all the SNPs have positive GWAS effects. The z-scores are used to compute the weighted average of the model weights (dashed lines), which have the same signs as and are proportional to the predicted effects of protein and gene expression on the GWAS outcome.

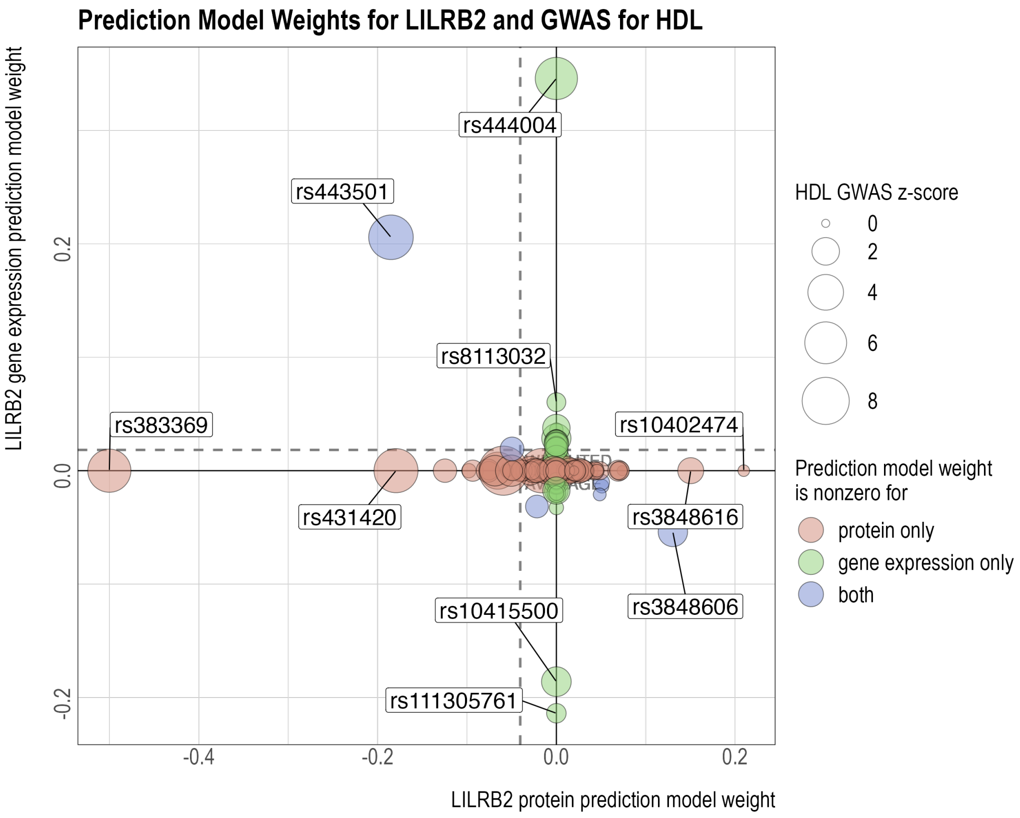

Figure S33: GWAS for HDL and prediction models for MICB's protein and gene expression levels. The reference and alternative alleles for GWAS and the predictive models have been aligned and reordered so that all the SNPs have positive GWAS effects. In the center and bottom panels, the size of the circles indicates the SNP's GWAS z-score. The z-scores are used to compute the weighted average of the model weights (dashed line), which has the same sign as and is proportional to the predicted effect of protein or gene expression on the GWAS outcome.

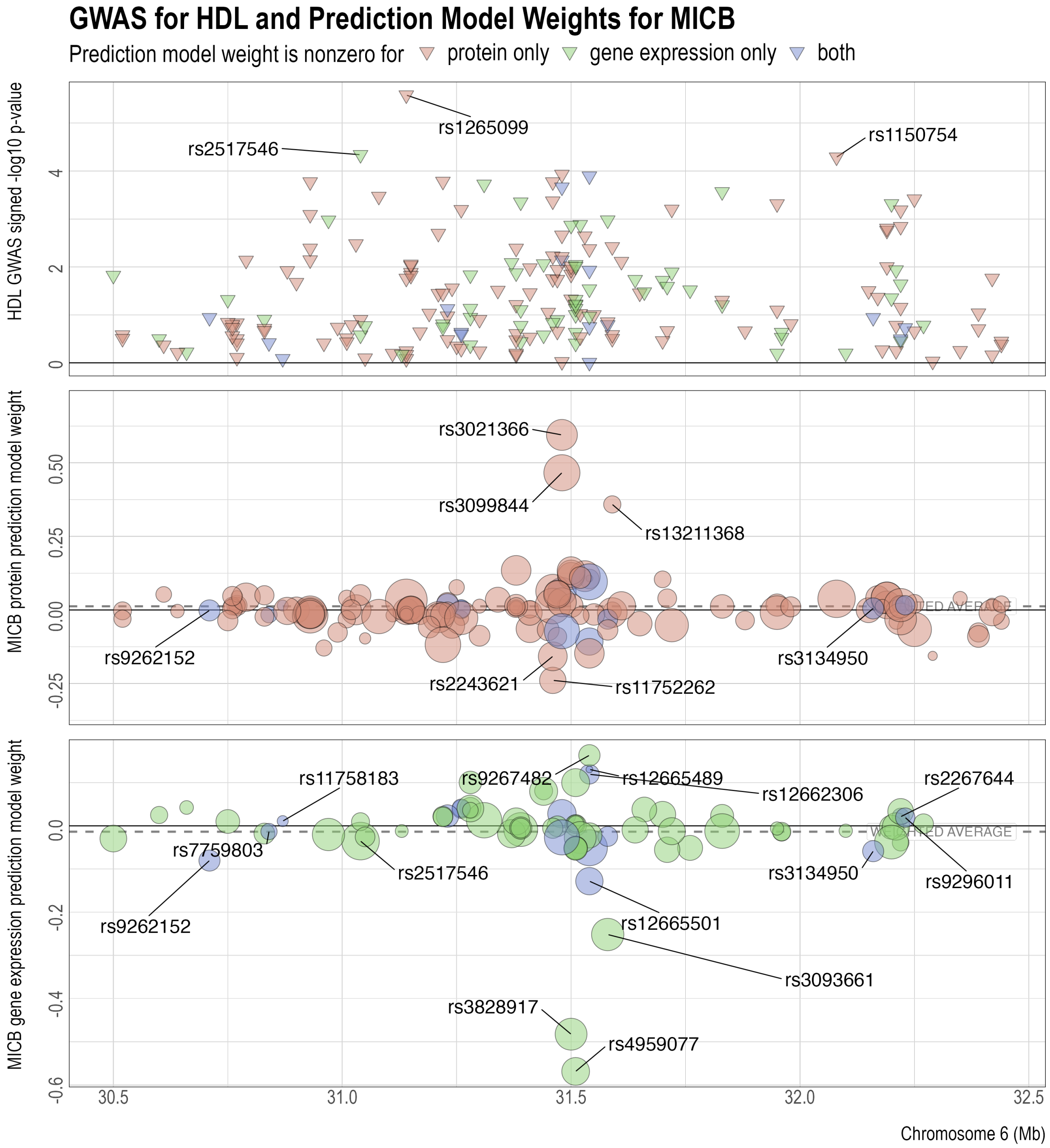

Figure S34: Comparison of MICB's protein and gene expression predictive model weights with the HDL GWAS z-scores of the SNPs. The reference and alternative alleles for GWAS and the predictive models have been aligned and reordered so that all the SNPs have positive GWAS effects. The z-scores are used to compute the weighted average of the model weights (dashed lines), which have the same signs as and are proportional to the predicted effects of protein and gene expression on the GWAS outcome.

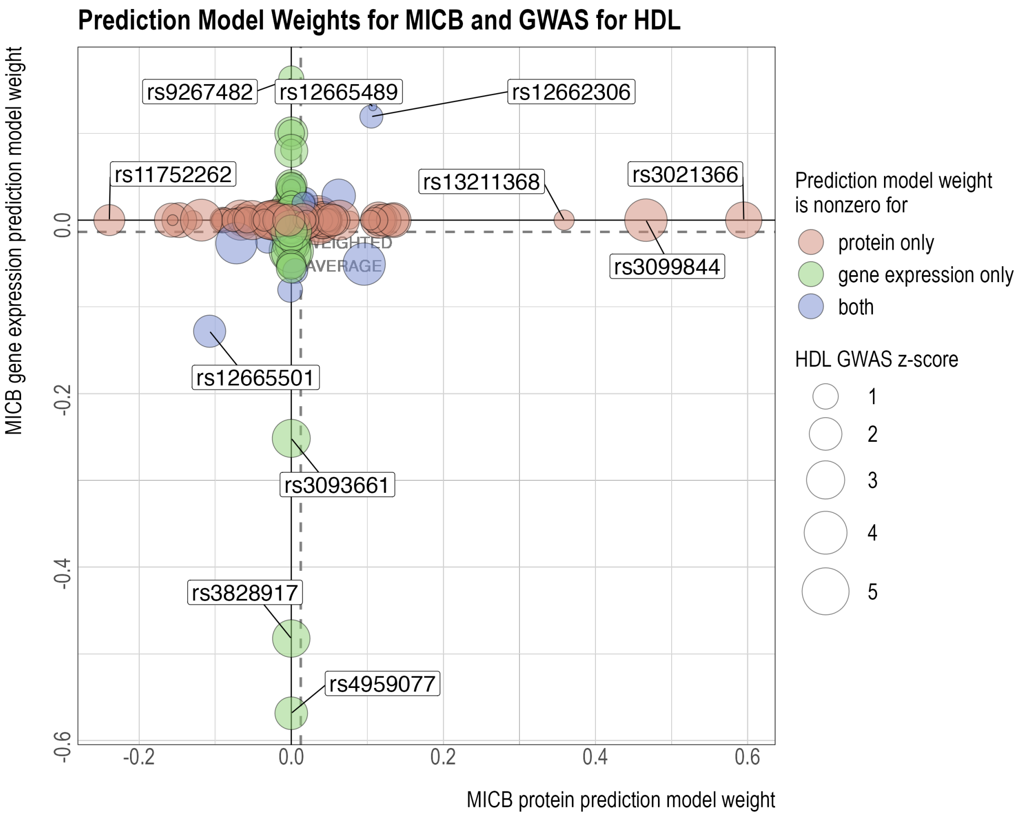

Figure S35: Comparison of MESA whole blood PWAS, MESA PBMC TWAS, and GTEx tissue-specific TWAS results for HDL. Panel (a): signed log p-value and significance of association. Missing values are shown in white. Significance of association is deteremined by the false discovery rate (FDR) threshold of 0.05. Panel (b): correlation between signed log p-values of MESA whole blood PWAS and signed log p-values of each GTEx tissue-specific TWAS (i.e. the correlation between the bottom row and every other row of the grid in Panel (a)).

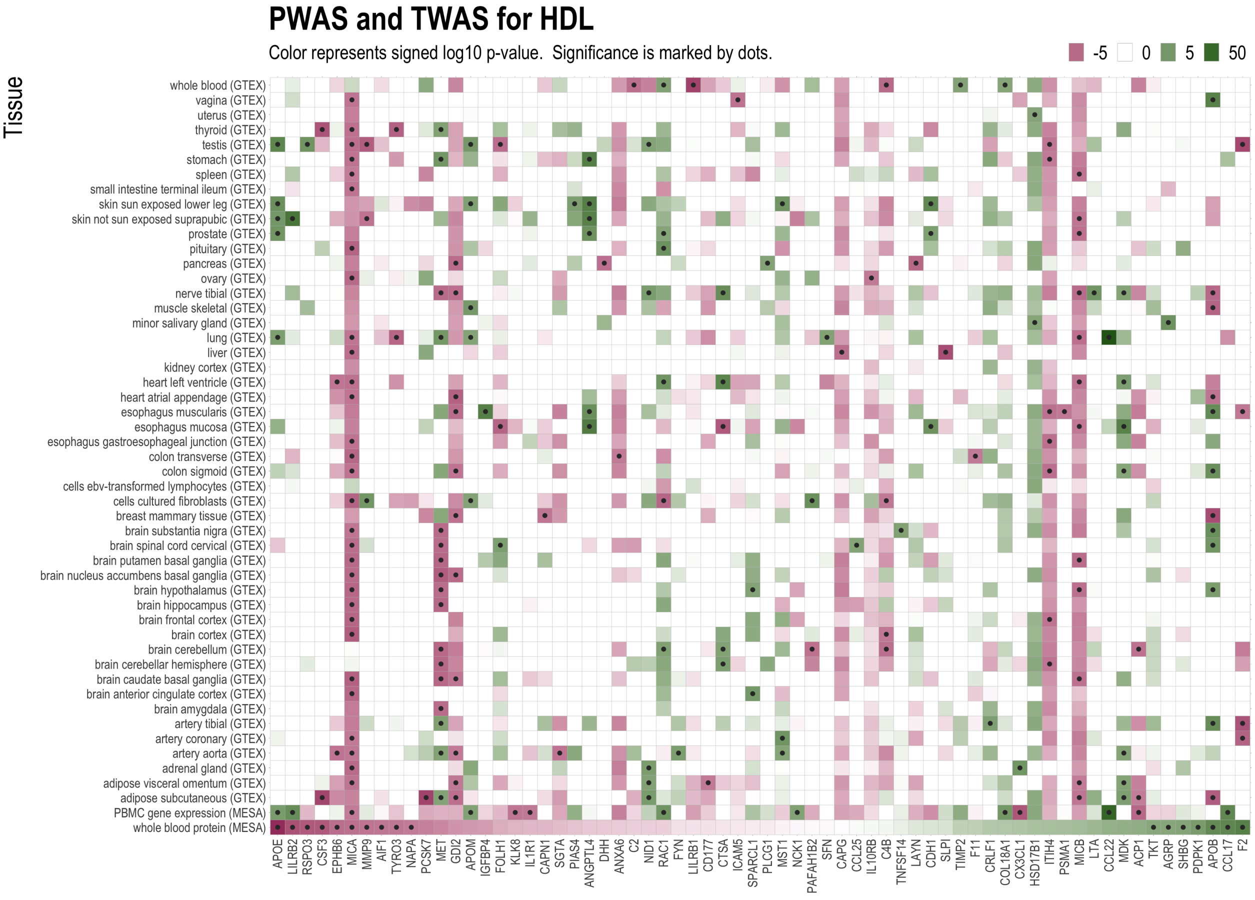

(a)

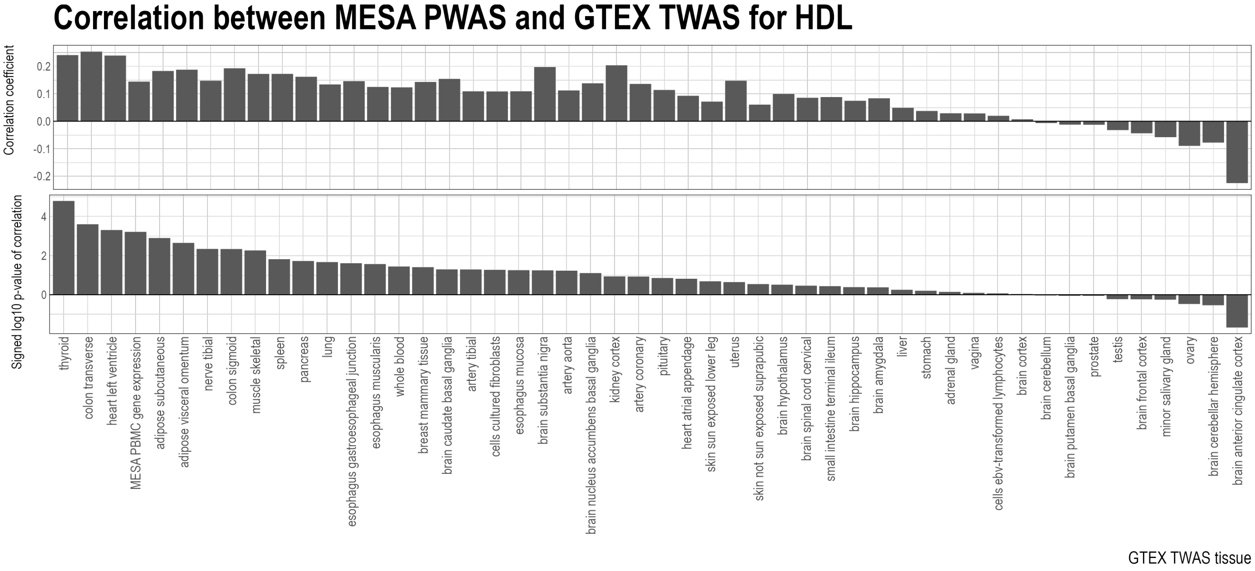

(b)

Figure S36: Correlation between the abundance levels of proteins and gene expressions in the MESA data. The histogram shows the distribution of this correlation for all the genes.

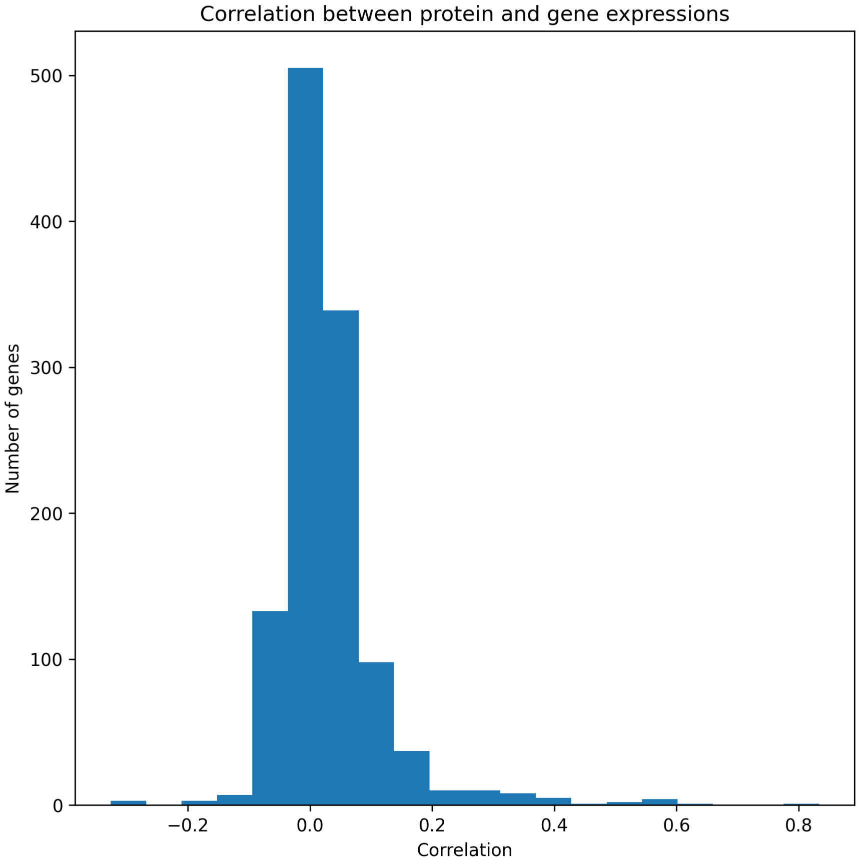

Figure S37: Comparison of the protein-gene expression correlation for the abundance levels directly measured in MESA and those predicted by the models trained using the MESA data. Each dot represents a gene.

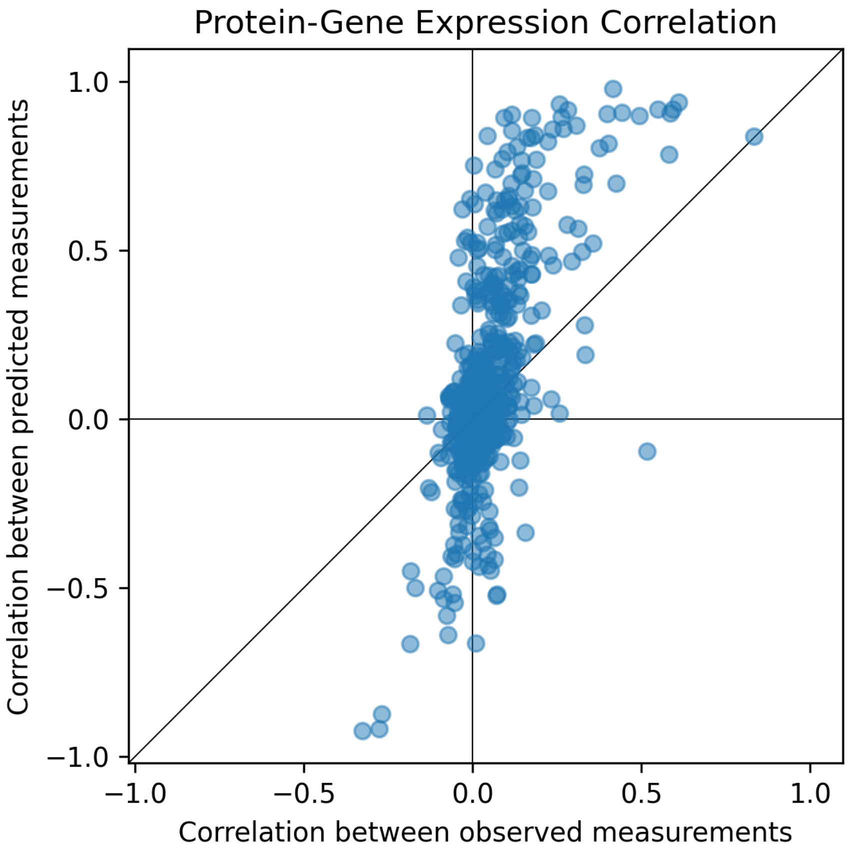

Figure S38: Linkage Disequilibrium of APOE cis SNPs. Only SNPs with large weights in the protein or gene expression predictive model (marked by a P or G before the SNP name, respectively) are displayed.

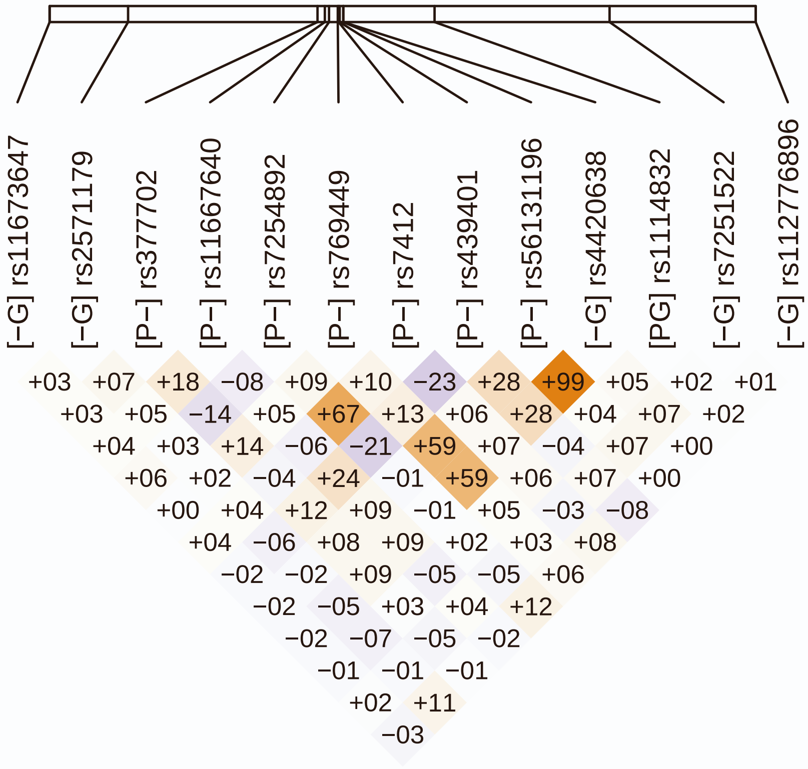

Figure S39: Linkage Disequilibrium of FCGR2B cis SNPs. Only SNPs with large weights in the protein or gene expression predictive model (marked by a P or G before the SNP name, respectively) are displayed.

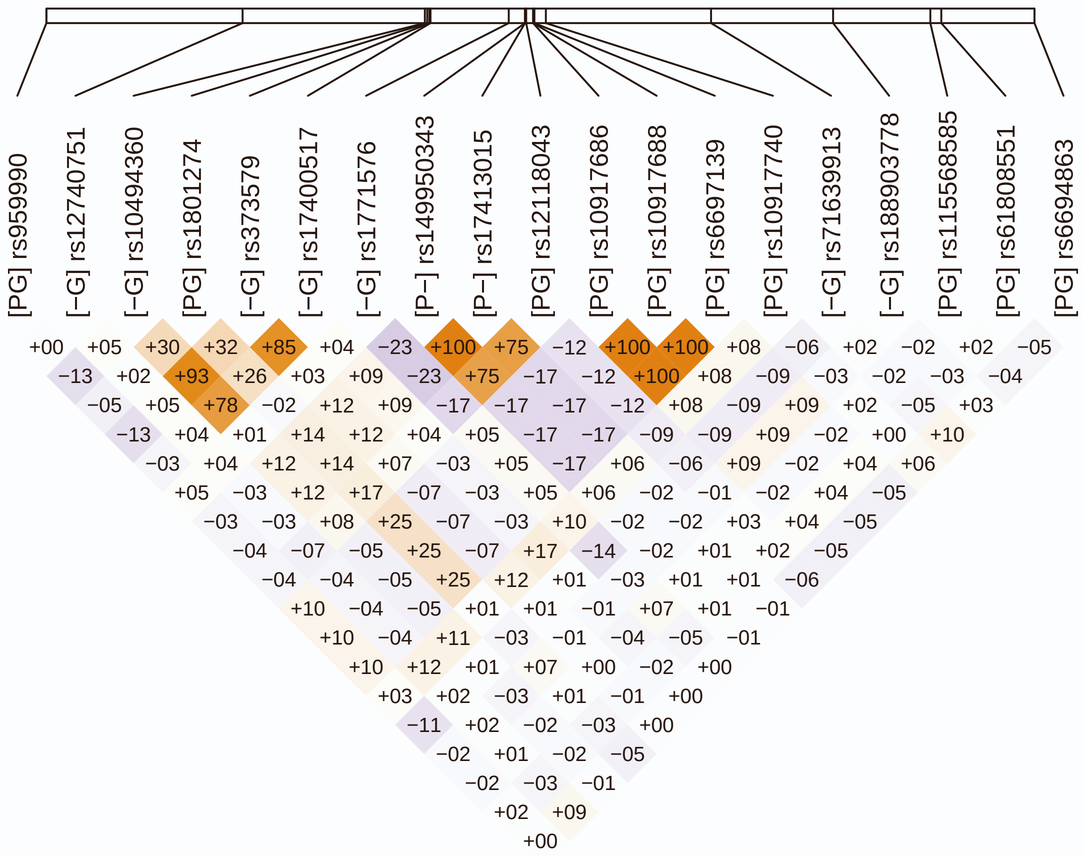

Figure S40: Linkage Disequilibrium of LILRB2 cis SNPs. Only SNPs with large weights in the protein or gene expression predictive model (marked by a P or G before the SNP name, respectively) are displayed.

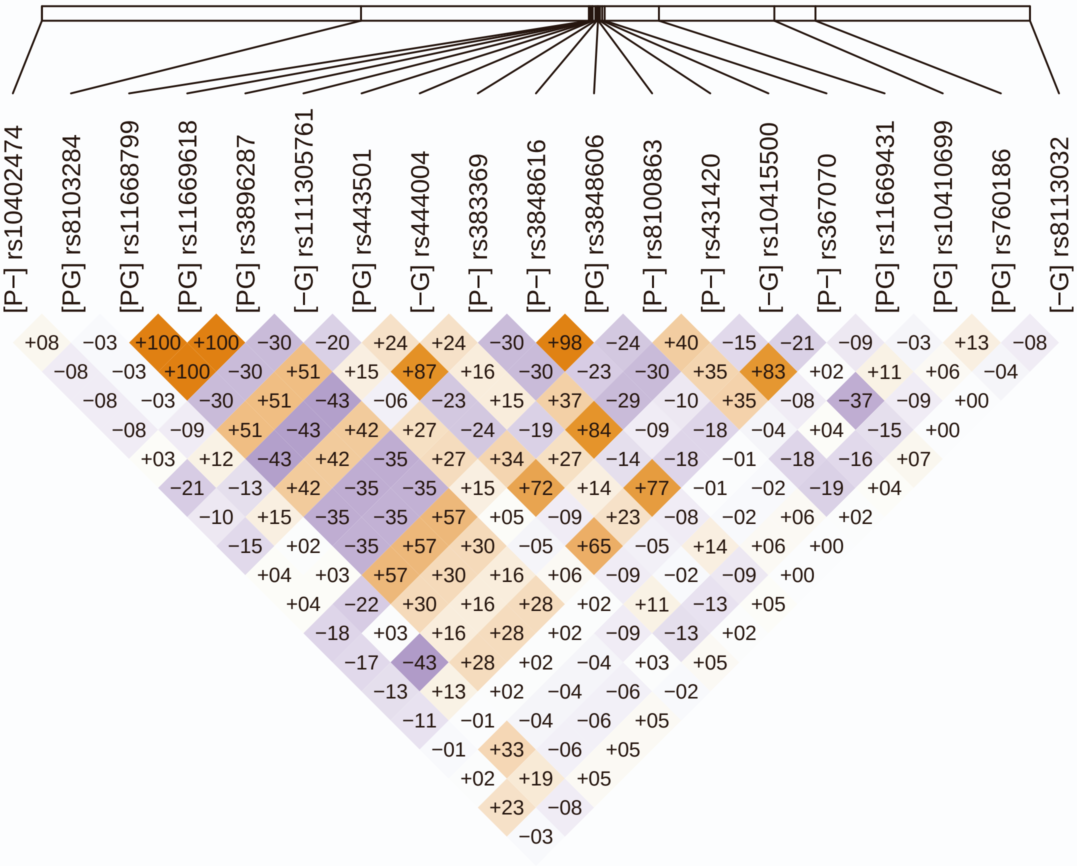

Figure S41: Linkage Disequilibrium of MICB cis SNPs. Only SNPs with large weights in the protein or gene expression predictive model (marked by a P or G before the SNP name, respectively) are displayed.

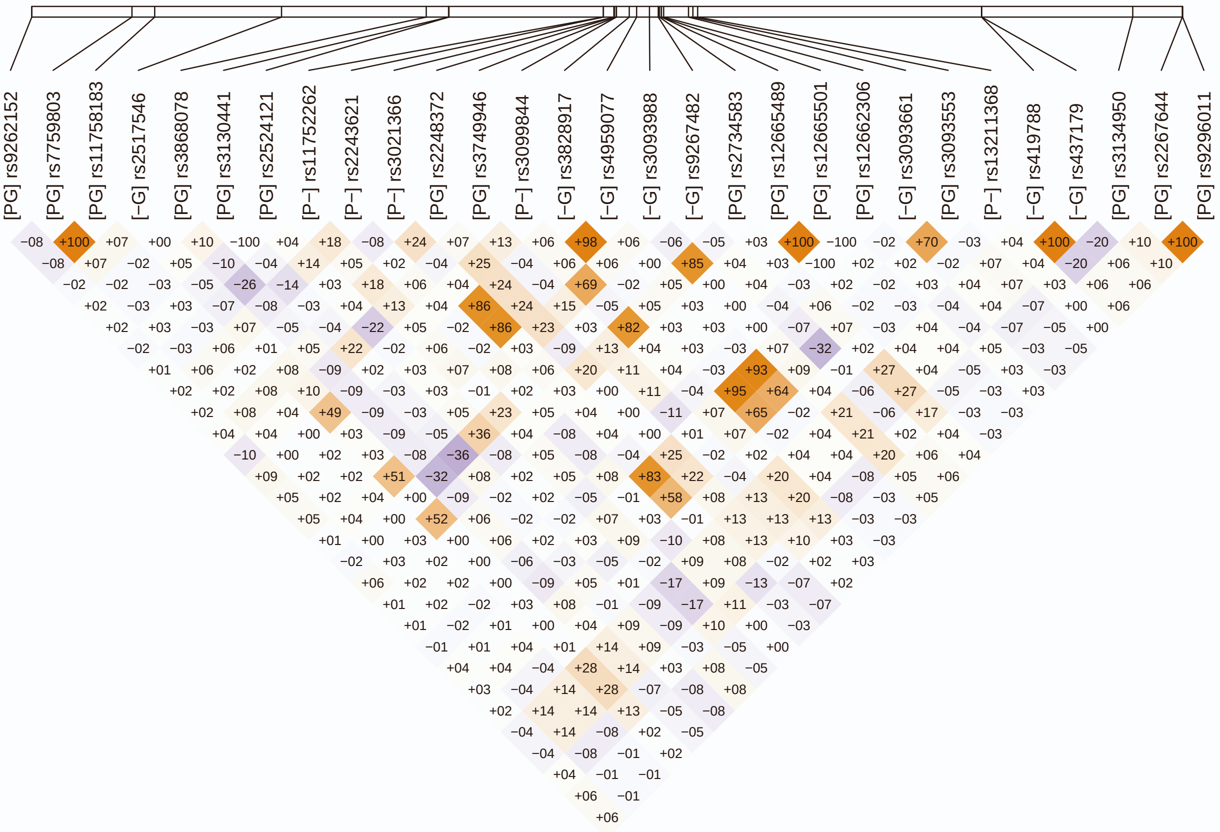

Figure S42: Correlation between signed log p-values of MESA PBMC TWAS for TC and signed log p-values of GTEx tissue-specific TWAS for TC.

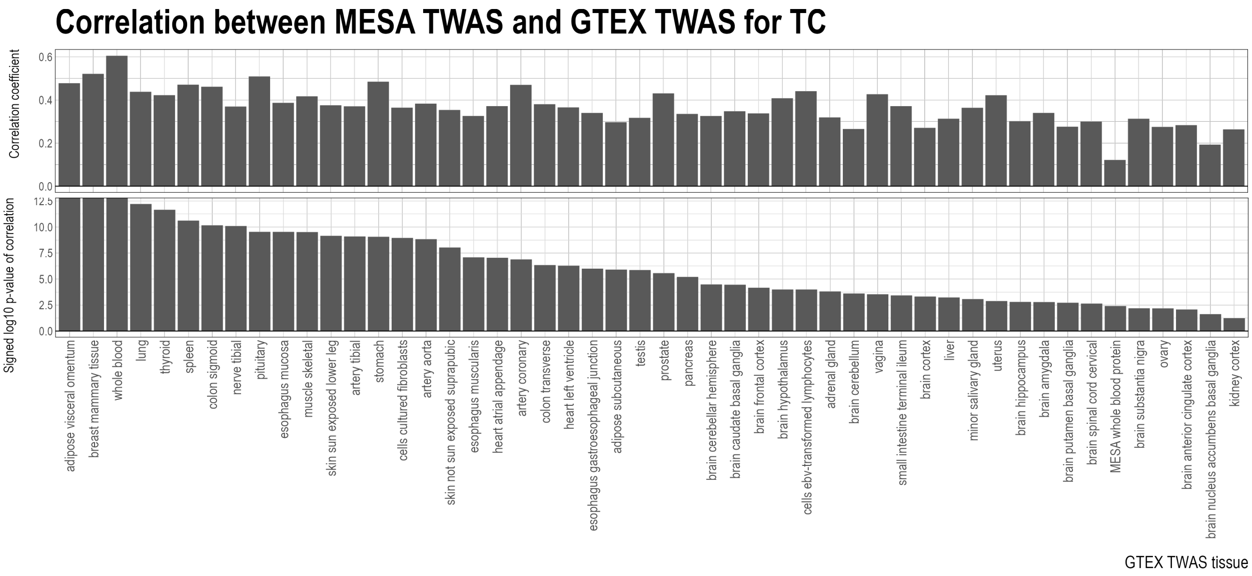

Figure S43: Correlation between signed log p-values of MESA PBMC TWAS for TG and signed log p-values of GTEx tissue-specific TWAS for TG.

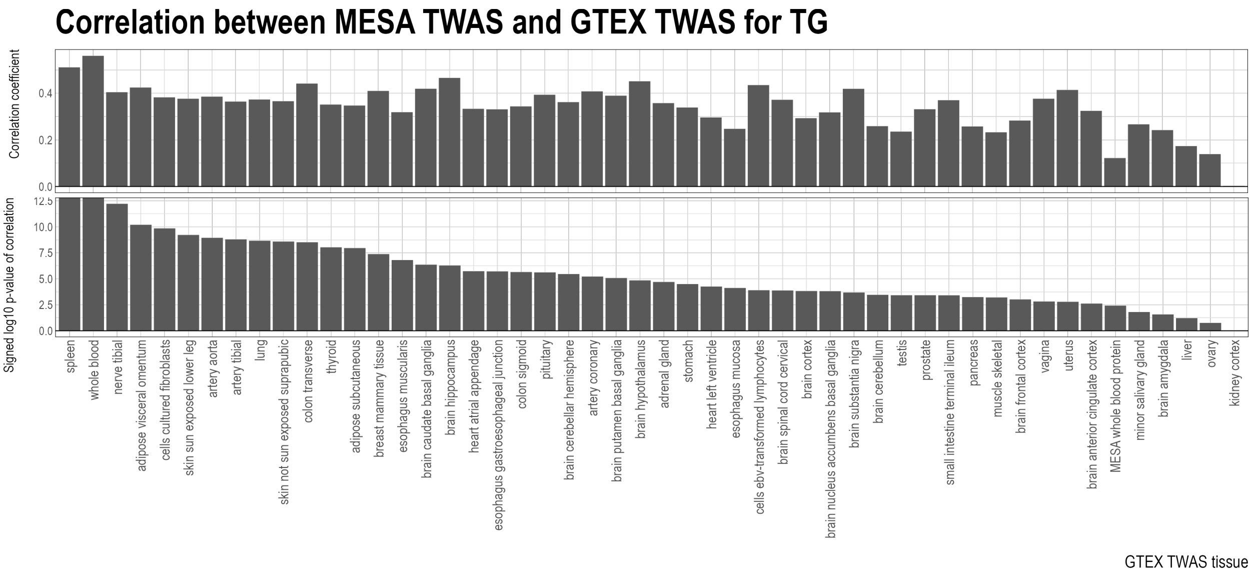

Figure S44: Correlation between signed log p-values of MESA PBMC TWAS for HDL and signed log p-values of GTEx tissue-specific TWAS for HDL.

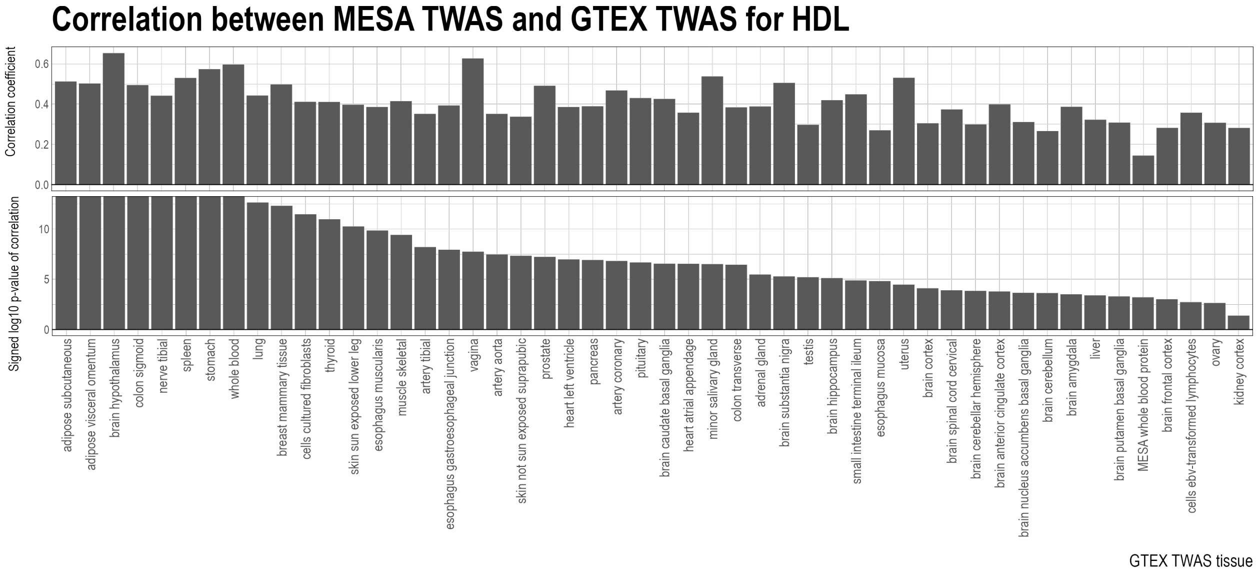

Figure S45: Correlation between signed log p-values of MESA PBMC TWAS for LDL and signed log p-values of GTEx tissue-specific TWAS for LDL.

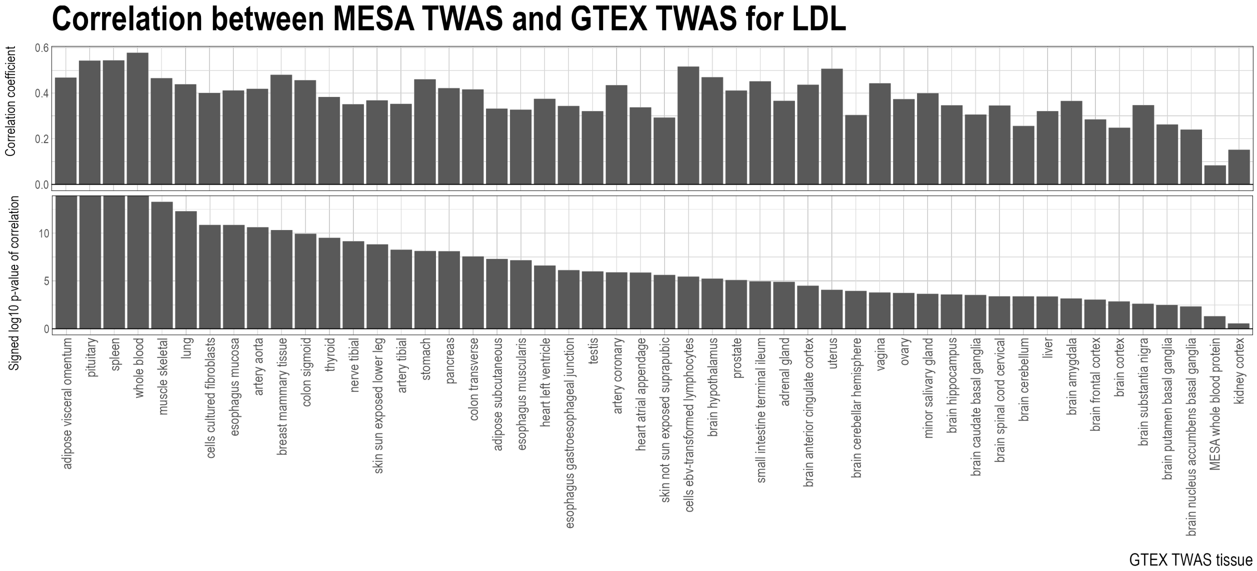

Figure S46: LDL GWAS and APOE protein QTL results used in the colocalization analysis.

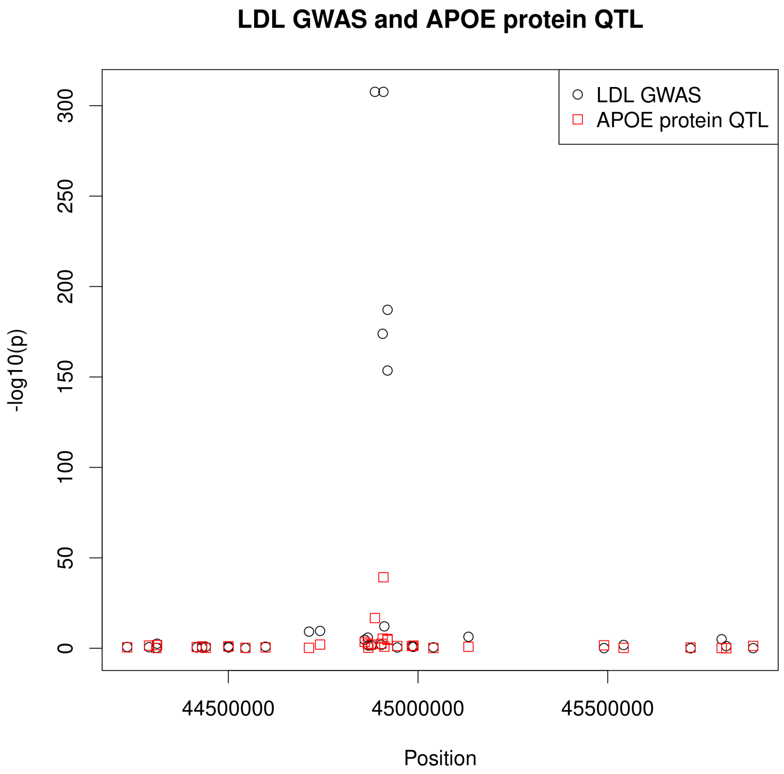

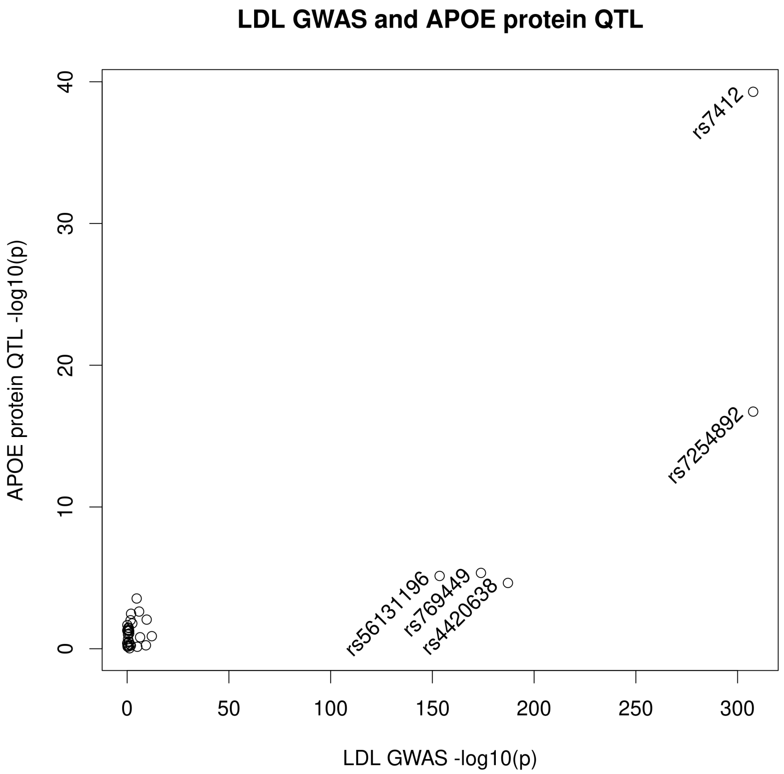

Table S1: Data characteristics of the MESA dataset. For continuous variables, the mean (and standard deviation) are displayed.

Self-reported race	Asian (7%)	Black (20%)	Hispanic (31%)	White (43%)
TC (mg/dl)	196 (29)	189 (39)	197 (35)	197 (33)
TG (mg/dl)	150 (74)	93 (42)	145 (66)	126 (63)
HDL (mg/dl)	49 (11)	52 (14)	48 (12)	53 (15)
LDL (mg/dl)	117 (26)	118 (34)	119 (33)	119 (30)
Age (mg/dl)	62 (10)	61 (10)	59 (09)	61 (10)
Sex (female)	45%	59 %	53 %	52 %
Using lipid medication	22%	13 %	12 %	19 %

Table S2: Colocalization analysis between GLGC GWAS, protein QTLs, and gene expression QTLs. Displayed are the probabilities computed by COLOC for insufficient power (P0+P1+P2), independent signals (P3), and colocalization (P4).

APOE	P0+P1+P2	P3	P4
TC-protein	0.00	0.00	1.00
TG-protein	0.00	1.00	0.00
HDL-protein	0.00	0.00	0.99
LDL-protein	0.00	0.00	1.00
TC-gene expression	0.94	0.01	0.03
TG-gene expression	0.91	0.01	0.07
HDL-gene expression	0.86	0.01	0.12
LDL-gene expression	0.94	0.01	0.03
protein-gene expression	0.94	0.01	0.03

FCGR2B	P0+P1+P2	P3	P4
TC-protein	0.03	0.00	0.95
TG-protein	0.99	0.00	0.00
HDL-protein	0.99	0.00	0.00
LDL-protein	0.03	0.00	0.96
TC-gene expression	0.02	0.00	0.96
TG-gene expression	0.99	0.00	0.00
HDL-gene expression	0.98	0.00	0.00
LDL-gene expression	0.02	0.00	0.97
protein-gene expression	0.00	1.00	0.00

LILRB2	P0+P1+P2	P3	P4
TC-protein	0.00	0.99	0.00
TG-protein	0.96	0.00	0.03
HDL-protein	0.00	1.00	0.00
LDL-protein	0.99	0.00	0.00
TC-gene expression	0.00	0.98	0.00
TG-gene expression	0.97	0.00	0.01
HDL-gene expression	0.00	1.00	0.00
LDL-gene expression	0.99	0.00	0.00
protein-gene expression	0.00	0.99	0.00

MICB	P0+P1+P2	P3	P4
TC-protein	0.00	0.62	0.37
TG-protein	0.00	0.99	0.00
HDL-protein	0.04	0.60	0.35
LDL-protein	0.01	0.95	0.03
TC-gene expression	0.00	1.00	0.00
TG-gene expression	0.00	1.00	0.00
HDL-gene expression	0.06	0.91	0.01
LDL-gene expression	0.01	0.96	0.02
protein-gene expression	0.00	1.00	0.00
