# Supplementary material for: Proteome-wide association studies for blood lipids and comparison with transcriptome-wide association studies
Source: HGG Adv. 2024 Nov 14;6(1):100383. doi: 10.1016/j.xhgg.2024.100383 (PMC11650301; doi:10.1016/j.xhgg.2024.100383)
Supplement: Document S2. Article plus supplemental information [file mmc2.pdf]

HGG
Advances

ARTICLE

Proteome-wide association studies for blood lipids and comparison with transcriptome-wide association studies

Daiwei Zhang,[1,2,16] Boran Gao,[2] Qidi Feng,[2,3] Ani Manichaikul,[6] Gina M. Peloso,[11] Russell P. Tracy,[7] Peter Durda,[8] Kent D. Taylor,[5] Yongmei Liu,[9] W. Craig Johnson,[10] Stacey Gabriel,[13] Namrata Gupta,[13] Joshua D. Smith,[14] Francois Aguet,[3] Kristin G. Ardlie,[3] Thomas W. Blackwell,[15] Robert E. Gerszten,[12] Stephen S. Rich,[6] Jerome I. Rotter,[5] Laura J. Scott,[2,*] Xiang Zhou,[2,*] and Seunggeun Lee[4,2,17,*]

Summary

Blood lipid traits are treatable and heritable risk factors for heart disease, a leading cause of mortality worldwide. Although genome-wide association studies (GWASs) have discovered hundreds of variants associated with lipids in humans, most of the causal mechanisms of lipids remain unknown. To better understand the biological processes underlying lipid metabolism, we investigated the associations of plasma protein levels with total cholesterol (TC), triglycerides (TG), high-density lipoprotein (HDL) cholesterol, and low-density lipoprotein (LDL) cholesterol in blood. We trained protein prediction models based on samples in the Multi-Ethnic Study of Atherosclerosis (MESA) and applied them to conduct proteome-wide association studies (PWASs) for lipids using the Global Lipids Genetics Consortium (GLGC) data. Of the 749 proteins tested, 42 were significantly associated with at least one lipid trait. Furthermore, we performed transcriptome-wide association studies (TWASs) for lipids using 9,714 gene expression prediction models trained on samples from peripheral blood mononuclear cells (PBMCs) in MESA and 49 tissues in the Genotype-Tissue Expression (GTEx) project. We found that although PWASs and TWASs can show different directions of associations in an individual gene, 40 out of 49 tissues showed a positive correlation between PWAS and TWAS signed p values across all the genes, which suggests high-level consistency between proteome-lipid associations and transcriptome-lipid associations.

Introduction

Blood lipid levels, including levels of total cholesterol (TC), triglycerides (TG), high-density lipoprotein (HDL) cholesterol, and low-density lipoprotein (LDL) cholesterol, are heritable risk factors[1] for coronary heart disease and stroke,[2,3] which are leading causes of death in the United States and other nations.[4,5] Genome-wide association studies (GWASs) have identified hundreds of loci that are significantly associated with at least one lipid trait in humans.[6–9] Variant alleles associated with higher concentration of LDL are more abundant among subjects with coronary artery disease than those without.[10] In addition, GWASs on lipids have facilitated the discovery of biological processes involved in lipoprotein metabolism.[11–13]

Although GWASs have been successful in identifying loci associated with lipids, they explain only a small proportion of the heritability,[14] estimated to be 35%–60% for TG, HDL, and LDL.[15] Moreover, most of these variants are located in non-coding regions with unclear functional roles.[16] Because of population stratification and linkage disequilibrium (LD), it is difficult to pinpoint the exact causal variants.[17] In addition, the large number of candidate variants severely limits the statistical power of GWASs.[18,19]

To boost the statistical power of GWASs and provide biologically meaningful interpretations, it is important to analyze downstream "omic" molecules, which include epigenetic, transcriptomic, and proteomic measurements, and then test their associations with phenotypes of interest. Recent multi-omic studies have elucidated the molecular mechanism of complex diseases.[20–24] When downstream omic measurements are not available, which is true for many of the trait- and disease-based GWASs, the genetically expected omic values can be imputed using

[1]Department of Biostatistics, Epidemiology, and Informatics, Perelman School of Medicine, University of Pennsylvania, Philadelphia, PA, USA; [2]Department of Biostatistics and Center for Statistical Genetics, University of Michigan, Ann Arbor, MI, USA; [3]Broad Institute of Massachusetts Institute of Technology and Harvard, Cambridge, MA, USA; [4]Graduate School of Data Science, Seoul National University, Seoul, Republic of Korea; [5]The Institute for Translational Genomics and Population Sciences, Department of Pediatrics, The Lundquist Institute for Biomedical Innovation at Harbor-UCLA Medical Center, Torrance, CA, USA; [6]Center for Public Health Genomics, University of Virginia, Charlottesville, VA, USA; [7]Departments of Pathology and Laboratory Medicine, and Biochemistry, Larner College of Medicine, University of Vermont, Burlington, VT, USA; [8]Department of Pathology and Laboratory Medicine, Larner College of Medicine, University of Vermont, Burlington, VT, USA; [9]Department of Medicine, Divisions of Cardiology and Neurology, Duke University Medical Center, Durham, NC, USA; [10]Department of Biostatistics, University of Washington, Seattle, WA, USA; [11]Department of Biostatistics, Boston University School of Public Health, Boston, MA, USA; [12]Division of Cardiovascular Medicine, Beth Israel Deaconess Medical Center, Boston, MA, USA; [13]Genomics Platform, Broad Institute of Massachusetts Institute of Technology and Harvard, Cambridge, MA, USA; [14]Department of Genome Sciences, Human Genetics, and Translational Genomics, University of Washington, Seattle, WA, USA; [15]Department of Biochemistry and Molecular Biophysics, Washington University School of Medicine, St. Louis, MO, USA; [16]Departments of Biostatistics and Genetics, University of North Carolina, Chapel Hill, NC, USA
[17]Lead contact
*Correspondence: ljst@umich.edu (L.J.S.), lee7801@snu.ac.kr (S.L.)
https://doi.org/10.1016/j.xhgg.2024.100383.
© 2024 The Authors. Published by Elsevier Inc. on behalf of American Society of Human Genetics.
This is an open access article under the CC BY license (http://creativecommons.org/licenses/by/4.0/).

prediction models built upon omic and genetic data from a separate study.[25–27] An association test is then conducted on each gene between the GWAS trait and the imputed omic level. For example, based on imputed gene expression measurements, transcriptome-wide association studies (TWASs)[28–30] have been performed for various diseases and clinical characteristics, such as schizophrenia,[31] breast cancer,[32] and structural neuroimaging traits.[33]

In addition to transcriptomics, proteomics provide further information for understanding complex diseases, since protein levels are downstream products of gene expression and can be more directly related to biological processes.[34] Compared to TWAS, fewer proteome-wide association studies (PWASs), imputation based or not, have been performed. Existing PWASs have investigated the associations between proteins and colorectal cancer,[19] stroke,[35] Alzheimer disease,[34] depression,[36] post-traumatic stress disorder,[37] and other psychiatric disorders.[38] Regarding blood lipids, although TWASs have identified hundreds of genes associated with them,[39–41] to the best of our knowledge, only one PWAS has been conducted for blood lipid traits.[42]

In this work, we investigated the association of blood protein abundance with blood lipid levels to identify proteins significantly associated with lipid variability. To conduct imputation-based PWASs, we trained genotype-based protein prediction models for protein levels measured from whole-blood samples from the Multi-Ethnic Study of Atherosclerosis (MESA).[43,44] The prediction models were then applied to the GWAS data of the Global Lipids Genetics Consortium (GLGC)[16] to identify proteins that are significantly associated with at least one of TC, TG, HDL, and LDL. Moreover, to study the relationship between PWAS and TWAS for lipids, we conducted an imputation-based TWAS for blood lipid traits using gene expression prediction models trained on samples from MESA peripheral blood mononuclear cells (PBMCs) and samples from 49 Genotype-Tissue Expression (GTEx) project tissues.[45] When comparing the TWAS and PWAS directions of association with lipid across all the genes on each of the 49 tissues, for most tissues, we found a positive correlation between the predicted PWAS and TWAS effects. However, for individual genes, we often observed the opposite predicted PWAS and TWAS directions of effects.

Material and methods

Ethics statement

This work was approved by the Health Sciences and Behavioral Sciences Institutional Review Board of the University of Michigan (IRB ID: HUM00152975). All data in this work were collected previously and analyzed anonymously.

Subjects

The MESA, a part of the Trans-Omics for Precision Medicine program (TOPMed),[46,47] investigates characteristics of subclinical cardiovascular diseases (i.e., those that are detected non-invasively before the onset of clinical signs and symptoms). The study aims to identify risk factors that can predict the progression of subclinical cardiovascular disease into clinically overt cardiovascular disease. The diverse, population-based sample includes 6,814 male and female subjects who are asymptomatic and aged between 45 and 84 years. The recruited participants consist of 38% White, 28% Black, 22% Hispanic, and 12% Asian (predominantly Chinese) individuals. In addition to genomic, transcriptomic, proteomic, and lipids data, the study also collected physiological, disease, demographic, lifestyle, and psychological factors.[43,44]

Preprocessing of MESA genotypes, proteomics, and transcriptomics

For the genotypes, we used the sequencing data from TOPMed.[46,47] We removed variants with a minor allele frequency of 0.05 or less among the TOPMed subjects, leaving 12,744,944 variants. Among the subjects who had genotypes, lipid levels, and demographic information, 1,438 of them were included in MESA. Samples with degrees of relatedness up to 2, as determined by KING,[48] were removed, which resulted in 1,403 subjects.

A total of 1,281 proteins were measured from 984 subjects. Protein levels were measured using a SomaScan HTS Assay 1.3K for plasma proteins. The SomaScan HTS Assay is an aptamer-based multiplex protein assay. It measures protein levels by the number of protein-specific aptamers that successfully bind to their target protein, although some proteins may be targeted by multiple aptamers.[42,49,50] In our analysis, targets that corresponded to multiple proteins were removed, which resulted in 1,212 proteins. As part of the TOPMed MESA Multi-Omics project, the 984 participants were selected for proteomic measurement based on the following criteria. First, participant samples were restricted to those already included in the TOPMed Whole Genome Sequencing effort.[46] Second, the race and ethnicity reflected those of participants in the parent MESA cohort. Third, participants were chosen to maximize the amount of overlapping omic data. Fourth, a substantial proportion of participants had biospecimens from MESA Exams 1 and 5.

Among these participants, 935 individuals whose protein levels were available had blood lipid measurements, genotypes, and covariate information. After inversely normalizing the protein levels, we computed the top 10 protein principal-component (PC) scores and the top 10 surrogate values[51] to detect outliers and adjust for unobserved factors that might adversely affect the analysis. Samples with p values less than 0.001 for the chi-squared statistics of either the PC scores or the surrogate values were removed, leaving 918 samples (see Table S1 for sample characteristics). The inversely normalized protein levels were then adjusted for age, sex, self-reported race and ethnicity, usage of lipid-lowering medications, top four genetic PCs, and top 10 surrogate values. The residuals of the protein levels were used for the subsequent analyses.

RNA sequencing was previously performed on MESA PBMCs.[52,53] We used the reads per kilobase of transcript per million reads mapped of each gene in our analysis. After applying the same preprocessing pipeline as for the proteomics (i.e., sample matching, inverse normalization, outlier removal, and adjustment for the same set of covariates), we had 1,021 samples for 22,791 genes, which covered 1,167 out of the 1,212 genes in the proteomic data.

Protein and gene expression prediction models based on MESA

Since MESA has a limited sample size for protein and gene expression measurements, we performed imputation-based PWAS for lipids by using SPrediXcan[54] to achieve higher statistical power. SPrediXcan

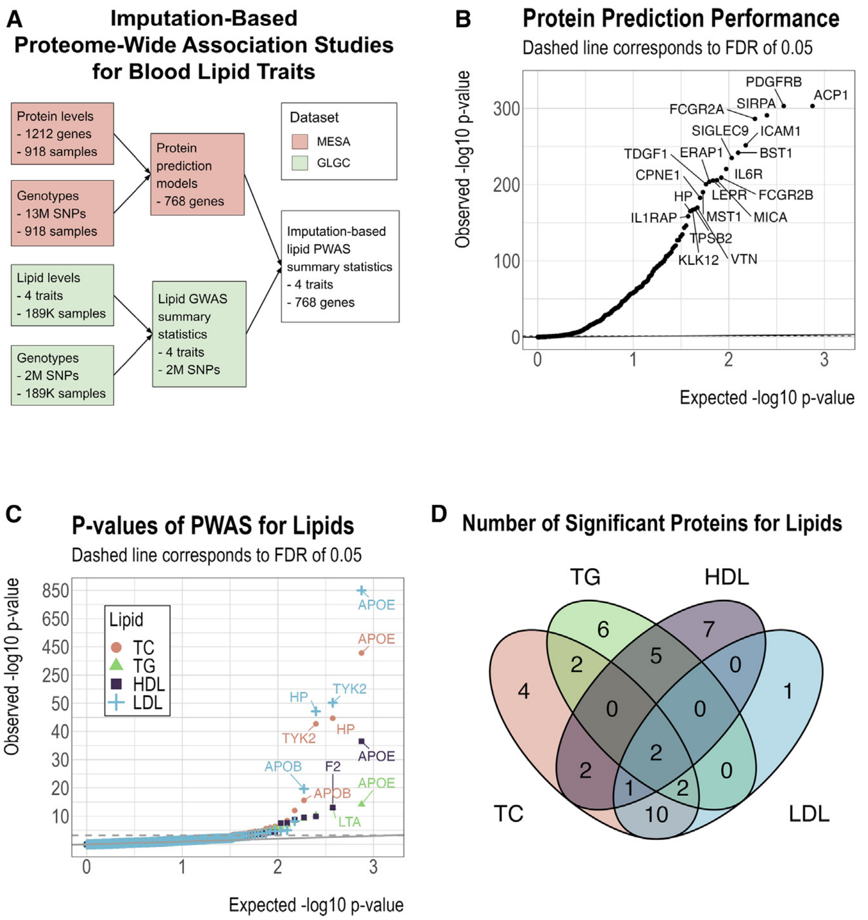

Figure 1. Basic characteristics of the imputation-based proteome-wide association studies (PWAS) for blood lipid traits in this work

(A) Schematic of PWAS for blood lipid traits.

(B and C) Protein prediction performance (B) and p values of PWASs for lipids (C). The solid line is the identity line, while the dashed line represents the false discovery rate (FDR) threshold of 0.05.

(D) Number of overlapping proteins significantly associated with each lipid.

builds an elastic net[55] prediction model of the omic measurements of each gene using its *cis*-SNPs as predictors. These prediction models are then combined with external GWAS summary statistics to predict the associations between the omic levels and the phenotypes of interest. Intuitively, this approach can be understood as an association study between observed phenotypes and predicted omic levels. Figure 1A illustrates the workflow of SPrediXcan. In our analysis, we trained the elastic nets on the MESA data to predict the preprocessed protein levels from the *cis*-SNPs within a window extending 1 MB upstream and 1 MB downstream of the protein's gene body (from the transcription start site to the transcription ending site). During model training, we restricted candidate-predictive SNPs to those that are included in the GWAS. The optimal elastic net penalty weights were selected by cross-validation as recommended for SPrediXcan.[54] We used the same procedure to build the predictive models for the transcriptomic data. After model training on the MESA data, we obtained non-trivial (i.e., at least one *cis*-SNP has a nonzero weight) prediction models for 749 out of 1,212 proteins and 886 out of 1,167 gene expressions, with an intersection of 562 genes that have both a non-trivial protein prediction model and a non-trivial gene expression prediction model.

Gene expression prediction models based on the GTEx project

The GTEx project[45] investigated the influence of regions in the human genome on gene expression and regulation in different tissues. Genotypes and gene expression levels were collected in 49 tissues from 900 postmortem donors, and the sample size for each tissue ranged from 73 to 706. In our analysis, we downloaded gene expression prediction models pre-trained using the GTEx version 8 data by the authors of SPrediXcan,[56] all of which had a predictive p value of less than 0.05. We applied the models to the GWAS summary statistics via the SPrediXcan framework to obtain tissue-specific TWAS results.

Imputation-based PWAS and TWAS using the GLGC

After training the elastic nets on the MESA data, we applied the prediction models to the GWAS summary statistics from the GLGC.[16] GLGC examined the associations between the genotypes and the lipid levels of 188,577 individuals of European ancestry. GWAS effect sizes and their SEs were obtained for more than 2 million SNPs. For each blood lipid trait, we applied the protein prediction models trained on the MESA data and the tissue-specific gene expression prediction models trained on both MESA and GTEx data to the GLGC summary statistics and computed the association between the lipid and the gene's protein and gene expression levels.

Results

Overview of PWAS results

Since our PWAS is imputation based, we assessed the prediction power of the *cis*-SNPs for the protein levels. The protein prediction models for MESA protein and genotype data were trained using the PredictDBPipeline framework,[57] which applies elastic net regression for protein prediction. Prediction performance metrics, including prediction p values and r^2, were calculated through 5-fold cross-validation on the MESA dataset. Figure 1B shows the prediction p values for the 749 proteins that have at least one predictive *cis*-SNP with a nonzero weight. The cumulative distribution function of the predictive r^2 is shown in Figure S1. With the false discovery rate (FDR) controlled at 0.05,[58] 469 (63%) of the 749 proteins were significantly predictable (Figures 1B and S1), and the predictive r^2 of these proteins ranged from 0.01 to 0.80 (Figure S1).

We next applied the protein prediction models to GLGC summary statistics to perform PWAS for TC, TG, HDL, and

Table 1. PWAS results for proteins that are significantly (FDR ≤0.05) associated with at least one blood lipid trait

	Lipid							
	TC		TG		HDL		LDL	
Gene	PWAS	TWAS	PWAS	TWAS	PWAS	TWAS	PWAS	TWAS
APOE	406(+)	16(−)	14(−)	.	37(−)	4(+)	850(+)	19(−)
TYK2	43(−)	.	.	.	.	.	53(−)	.
HP	45(−)	3(−)	4(−)	.	.	.	47(−)	3(−)
LTA	.	.	13(−)	.	.	.	.	.
MICB	12(+)	3(−)	9(+)	.	.	.	5(+)	.
CCL17	.	.	.	.	10(+)	9(+)	.	.
LILRB2	4(−)	4(+)	.	.	10(−)	8(+)	.	.
RBM39	8(−)	.	.	.	.	.	5(−)	.
PCSK7	4(−)	3(−)	8(−)	4(−)	.	.	.	.
FN1	7(+)	.	.	.	.	.	8(+)	.
RSPO3	.	.	6(+)	.	8(−)	.	.	.
PDPK1	.	.	7(−)	.	4(+)	.	.	.
MICA	6(−)	6(−)	.	.	4(−)	3(−)	4(−)	4(−)
IL-1RN	6(+)	.	.	.	.	.	3(+)	.
MMP9	.	.	5(+)	.	4(−)	.	.	.
FCGR2A	5(−)	6(−)	.	.	.	.	5(−)	6(−)
SERPINA1	4(−)	.	.	.	.	.	5(−)	.
ICAM5	5(−)	.	.	.	.	.	4(−)	.
EPHB6	.	.	.	.	4(−)	.	.	.
CTSB	.	.	4(+)	6(+)	.	.	.	.
HAVCR2	4(−)	.	.	.	.	.	3(−)	.
MET	.	.	4(+)	.	.	.	.	.
FCGR2B	3(−)	5(+)	.	.	.	.	4(−)	4(+)
ICAM3	4(+)	.	.	.	.	.	.	.
CPNE1	4(+)	6(+)	.	.	.	.	.	.
COLEC11	4(+)	.	.	.	.	.	.	.
AIF1	.	.	.	.	4(−)	.	.	.
HSPA1A	.	.	4(−)	.	.	.	.	.
TYRO3	.	.	3(+)	.	3(−)	.	.	.
MMP1	3(−)	3(−)	.	.	.	.	3(−)	.
SHBG	.	.	.	.	3(+)	.	.	.
VWF	.	.	.	.	.	.	3(+)	.
AGRP	.	.	.	.	3(+)	.	.	.
TKT	.	.	.	.	3(+)	.	.	.
CSF3	4(−)	.	.	.	8(−)	.	.	.
NAPA	.	.	.	.	3(−)	.	.	.
APOB	16(−)	.	10(−)	.	9(+)	.	20(−)	.
F2	.	.	5(−)	.	13(+)	.	.	.
HGFAC	6(−)	.	6(−)	.	.	.	.	.

(*Continued on next page*)

Table 1. *Continued*

	Lipid							
	TC		TG		HDL		LDL	
Gene	PWAS	TWAS	PWAS	TWAS	PWAS	TWAS	PWAS	TWAS
MDK	5(+)	.	.	.	.	.	.	.
BCAM	.	.	3(−)	.	.	.	.	.
CFC1	.	.	4(−)	.	.	.	.	.

Next to the PWAS summary statistics of every protein, the TWAS summary statistics of the same gene are also displayed. Inside each cell is the −log10 *p* value, followed by the direction of association in parentheses. Associations that are no significant at the threshold of FDR = 0.05 are replaced with a dot.

LDL. The quantile-quantile plot of the PWAS *p* values for each lipid is shown in Figure 1C. Overall, we observed that 23, 17, 17, and 16 proteins were significantly associated (FDR ≤0.05) with TC, TG, HDL, and LDL, respectively, and 42 proteins were significantly associated with at least one lipid (Figure 1D; Table 1). Among these proteins, apolipoprotein E (APOE), haptoglobin (HP), and interleukin-1 receptor antagonist (IL-1RN) have been identified for their associations with lipids in previous studies.[42]

Comparison of MESA-trained PWAS and MESA-trained TWAS

To compare lipid PWAS with lipid TWAS from the same study samples, we also conducted TWAS using GLGC summary data, with the predictive models trained on the MESA PBMC gene expression data. For each lipid trait, we compared the signed log *p* value of the genes in PWAS and TWAS and computed the Spearman correlation coefficient[59] (Figure 2), where the sign reflects the direction of association. The PWAS and TWAS signed log *p* values were modestly positively correlated, where the correlation coefficient ranged from 0.083 to 0.144 and all the correlation *p* values were below 0.05. For TC/TG/HDL/LDL, among the 23/17/17/16 genes whose proteins are associated with the lipid (Figure 1D; Table 1), 10/2/4/5 genes have both protein and gene expression associated with the lipid. Of these 10/2/4/5 genes, 6/2/2/3 genes' protein-lipid association direction and gene expression-lipid

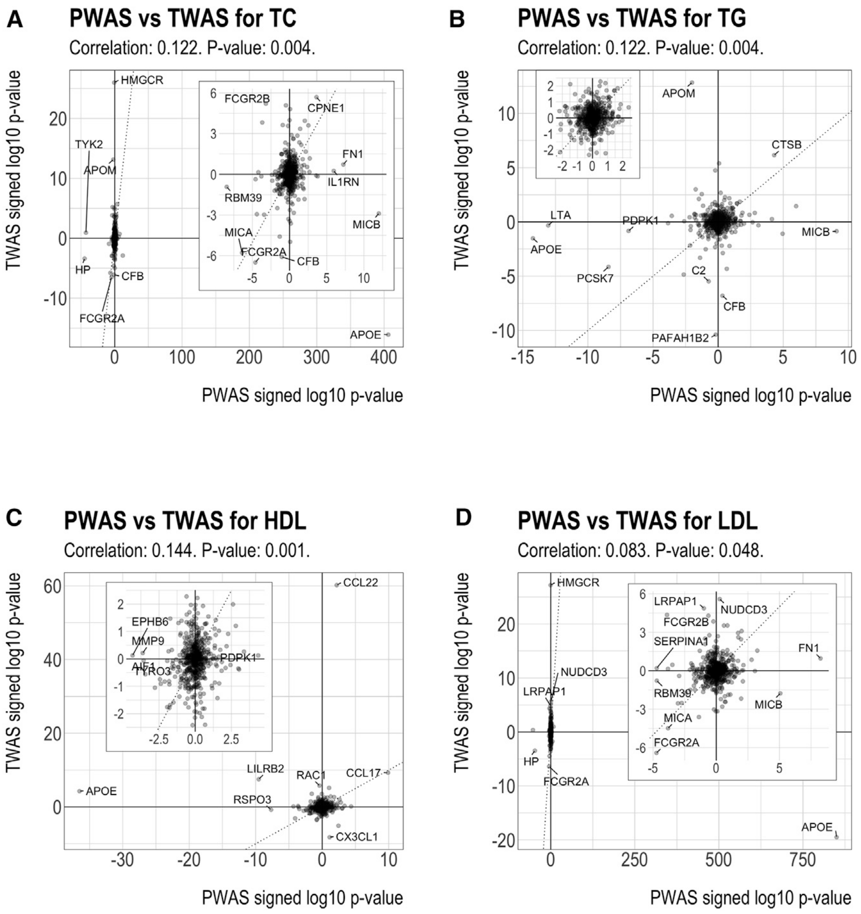

Figure 2. Comparison of PWASs and transcriptome-wide association studies (TWASs) results for lipids

The subplot inside each panel shows magnified results.

(A) PWAS vs. TWAS for total cholesterol (TC).

(B) PWAS vs. TWAS for triglycerides (TG).

(C) PWAS vs. TWAS for high-density lipoprotein (HDL) cholesterol.

(D) PWAS vs. TWAS for low-density lipoprotein (LDL) cholesterol.

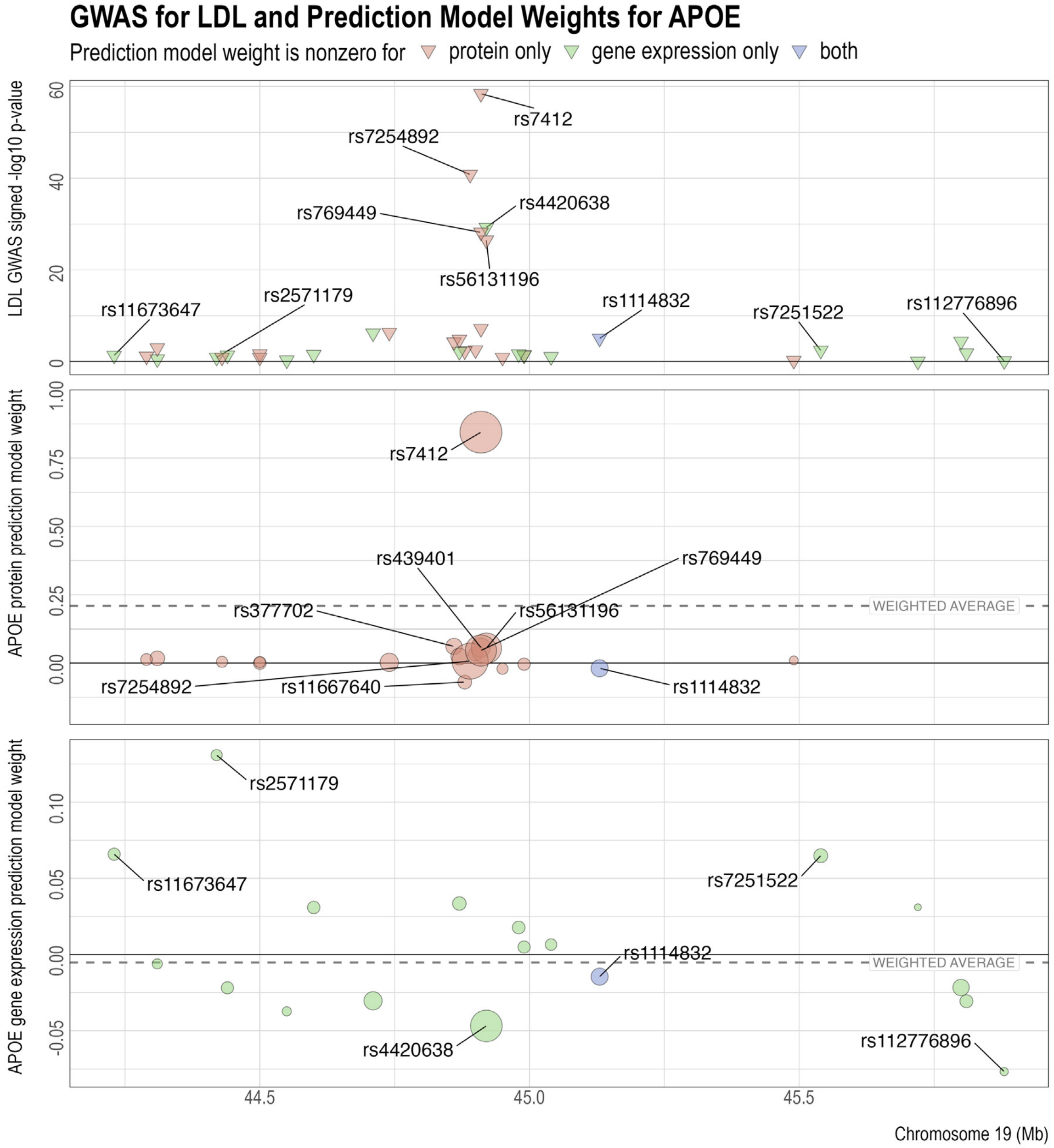

Figure 3. Genome-wide association studies (GWASs) for LDL and prediction models for the protein and gene expression levels of APOE

The reference and alternative alleles for GWAS and the predictive models have been aligned and reordered so that all the SNPs have positive GWAS effects. Center and bottom:, the size of the circles indicates the SNP's GWAS *Z* score. The *Z* scores are used to compute the weighted average of the model weights (dashed line), which has the same sign as and is proportional to the predicted effect of protein or gene expression on the GWAS outcome.

association direction are concordant. In particular, *APOE* was significantly and positively associated with LDL in PWAS but significantly and negatively associated with LDL in TWAS; leukocyte immunoglobulin-like receptor B2 (*LILRB2*) and Fc gamma receptor IIb (*FCGR2B*) were significantly negatively associated with two lipids in PWAS and positively associated with the same lipids in TWAS.

To better understand the opposing PWAS and TWAS effects in some of the genes, we used *APOE* and LDL as an example and compared the LDL GWAS summary statistics with the weights of the *cis*-SNPs in the protein and gene expression prediction models. Figure 3 (top) shows the signed log *p* values of the association between LDL and the *cis*-SNPs of *APOE* in GLGC. Effect alleles were chosen so that all the GWAS effect sizes for LDL were positive. Among SNPs with very significant GWAS *p* values, effect allele C in SNP rs7412 corresponds to the Apoε2 allele of *APOE*.[60,61] This SNP is related to the stability of the *APOE* isoforms[62] and is a risk factor for coronary heart disease.[63]

Another SNP with a very strong GWAS effect is rs4420638, whose effect allele G may elevate TC, TG, and HDL.[64] As indicated by the colors, the sets of predictive *cis*-SNPs for protein and gene expression have little overlap with each other, with only one SNP (rs1114832) having a nonzero weight in both predictive models.

Figure 3 (center) shows the weights of the *cis*-SNPs in the prediction model of APOE protein. The effects of most *cis*-SNPs on APOE protein had the same direction as their effects on LDL, with only four exceptions below the $y = 0$ line. In particular, the effects of rs7412 for LDL and APOE protein were both strong and of the same sign, dominating all the other *cis*-SNPs. Thus, the resulting association between APOE protein and LDL was positive, as indicated by the positive weighted average of the predictive weights (dashed line). However, compared to the PWAS results, the directions of the effects of the predictive *cis*-SNPs on *APOE* gene expression were approximately equally split between positive and negative, as shown in Figure 3 (bottom). Nevertheless, the negative weights outweighed the positive weights, with the greatest contribution from rs4420638 and rs112776896, which have a strong positive association with LDL but a strong negative association with *APOE* gene expression. Thus, the resulting association between LDL and *APOE* gene expression was negative, as indicated by the negative weighted average of the gene expression predictive weights (dashed line). Overall, due to the small proportion of overlapping nonzero predictive weights and their different directions of effects (Figure S2), *APOE* protein and gene expression have opposite directions of association with LDL. We also examined the LD between the SNPs with large weights in the protein or gene expression predictive model to investigate whether the driver SNPs in the protein and gene expression predictive models are correlated. We found that for *APOE* (Figure S38), the correlations between the protein driver SNPs and the gene expression driver SNPs are close to zero weak (e.g., 0.04 for rs7412 vs. rs2571179, −0.03 for rs7412 vs. rs7251522), which indicates that the disparity between the weights in the protein and gene expression models is not due to different driver SNPs tagging the same loci. Similar patterns were observed for LDL with other genes, such as *FCGR2B*, *LILRB2*, and major histocompatibility complex class I polypeptide-related sequence B (*MICB*) (Figures S3–S8 and S39–S41), as well as for the other lipids (Figures S9–S16, S18–S25, and S27–S34).

COLOC probabilities cluster more distinctly into different classes and thus, unlike other methods, suggest a natural cutoff threshold at $p = 0.5$. Another advantage of COLOC is that for genes with a low probability of colocalization, it further distinguishes distinct GWAS and expression quantitative trait loci (eQTL) signals from low power. This is a useful feature that future development of colocalization methods should also offer. SMR, however, uses its own estimate of "heterogeneity" of signals calculated by HEIDI.

In addition, since TWAS and PWAS can be contaminated by LD,[28,54] we performed colocalization analysis to investigate the probability of shared signals. This step was performed using COLOC,[65] which, compared to other colocalization analysis methods, has the advantage of being able to not only distinguish distinct signals from low power but also provide natural cutoffs for colocalization probabilities.[54,66] The results of the colocalization test varied greatly between different genes and lipids (Table S2). For example, APOE protein and LDL have a high probability of shared signals, which is strong evidence for colocalization and a shared causal variant. In particular, among the variants in the APOE gene, SNP rs7412 has a highly significant association with both the LDL level and the APOE protein abundance level (Figure S46). However, APOE protein and TG have a high probability of independent signals, which suggests the absence of shared causal variants. At the same time, the LDL-gene expression or protein-gene expression results for *APOE* do not have sufficient power to support or reject colocalization. The patterns are very different for other genes. For example, *FCGR2B* has high probabilities for lipid-protein colocalization and lipid-gene expression colocalization for TC and LDL, but the protein and gene expression signals have a probability of being independent. These findings demonstrate the heterogeneity among the associations between lipids, proteins, and gene expressions.

Comparison of MESA-trained PWAS and GTEx-trained TWAS

The TWAS results obtained from MESA only used gene expression measurements in PBMCs. Since the gene expression levels in some tissues, such as liver, may be more relevant to lipid levels compared to those in other tissues, we extended our TWAS analysis using gene expression data from 49 GTEx tissues. The results of MESA-trained PWAS, MESA-trained TWAS, and GTEx-trained TWAS are compared in Figures 4A, S17A, S26A, and S35A. Overall, for all lipids, the significance and direction of association for PWAS and TWAS are heterogeneous across individual genes. For some genes, the predicted protein and gene expression levels had very consistent directions of association with LDL. For example, for major histocompatibility complex class I polypeptide-related sequence A (*MICA*), LDL was positively associated with both protein and gene expression in MESA and with gene expression in 43 out of 49 tissues in GTEx. Other examples with similar patterns were observed for *MICA* with TC and HDL, copine 1 (*CPNE1*) with TC, and cathepsin B (*CTSB*) with TG. For some other genes, the protein and gene expression had mixed directions of association. For instance, LDL was positively associated with HP protein levels, but it had approximately equal numbers of positive and negative associations with gene expression levels across tissues. Similar inconsistent patterns were observed for *HP* with TC, *APOE* with TC and LDL, and apolipoprotein B (*APOB*) with TC, TG, and HDL.

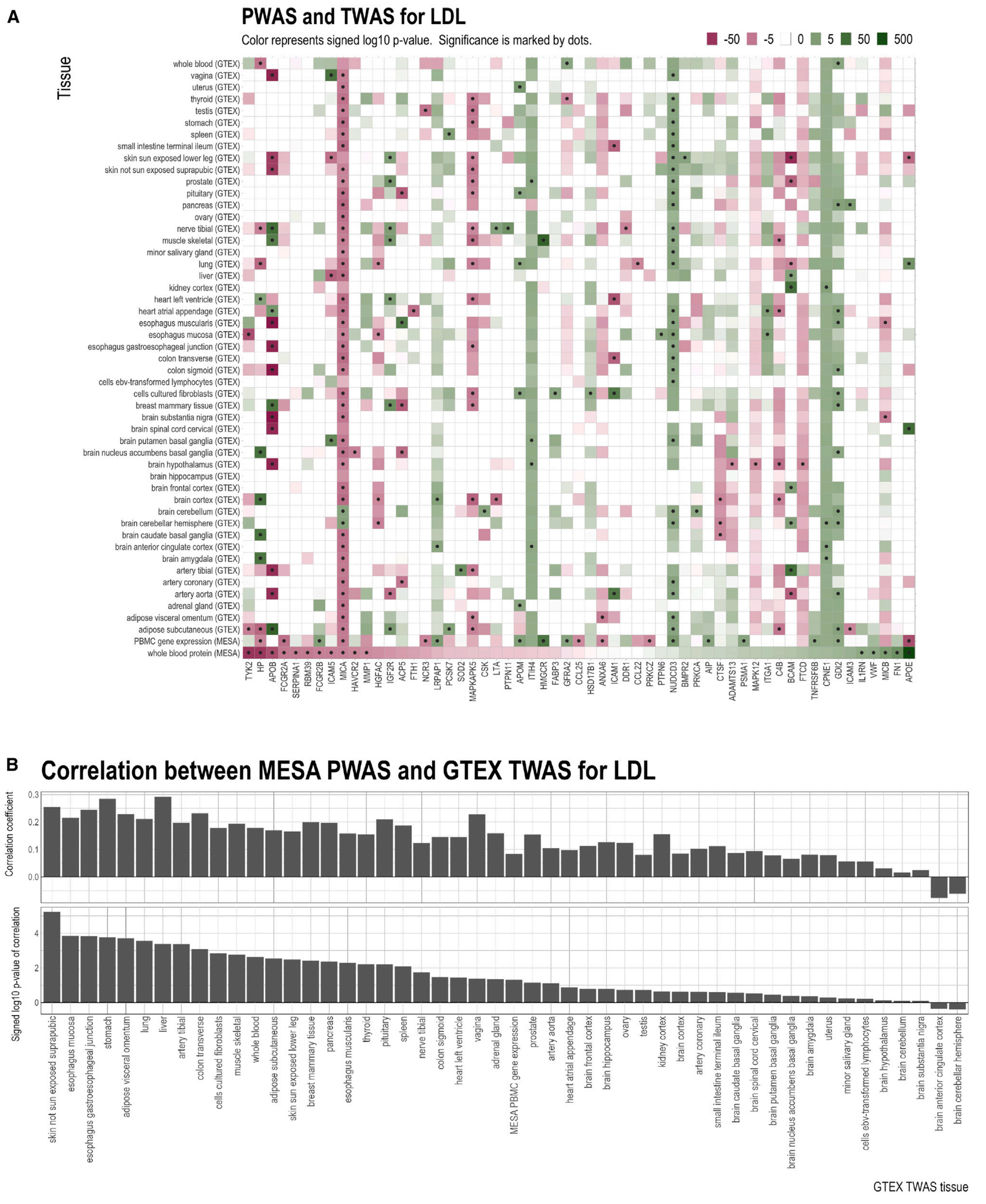

Figure 4. Comparison of MESA PBMC PWAS, MESA PBMC TWAS, and GTEx tissue-specific TWAS results for LDL
(A) Signed log *p* value and significance of association. Missing values are shown in white. Significance of association is determined by the FDR threshold of 0.05. Only genes with at least one significant association with LDL are displayed.
(B) Correlation between signed log *p* values of MESA PBMC PWAS and signed log *p* values of each GTEx tissue-specific TWAS (i.e., the correlation between the bottom row and every other row of the grid in A).

We next evaluated the correlation patterns of PWAS and TWAS effects when aggregated across all the genes and how this correlation varied across tissues. Figure 4B shows the Spearman correlations for each tissue between the signed log p values for MESA-trained PWAS and GTEx-trained TWAS for LDL. Of the 49 tissues in GTEx, the MESA-PWAS vs. GTEx-TWAS correlation was positive in 47 of them (binomial test p value: 2.2×10^{-12}). For TC, TG, and HDL, the corresponding correlations were positive in 41, 43, and 40 tissues, respectively (Figures S17B, S26B, and S35B). These findings indicate that although the relation between the effects of the proteins and the tissue-specific gene expressions on lipids can be mixed on a single gene, the aggregated correlations between TWAS and PWAS results for lipids across all genes were mostly positive, even if the gene expression predictive models and the protein predictive models were trained using different datasets (i.e., MESA and GTEx).

We also performed the same analysis for MESA-trained TWAS and GTEx-trained TWAS (Figures S42–S45). MESA-trained TWAS was obtained by applying MESA-trained gene expression prediction models to GLGC lipid GWAS, while GTEx-trained TWAS was obtained by applying GTEx-trained gene expression prediction models to GLGC lipid GWAS. The MESA-TWAS vs. GTEx-TWAS correlation was positive in all tissues, and both the magnitude and the significance of correlation were much higher than those of the MESA-PWAS vs. GTEx-TWAS correlation. Of the 49 GTEx tissues, the MESA-TWAS vs. GTEx-TWAS correlation was significant in 48/46/49/48 tissues for TC/TG/HDL/LDL. Moreover, recall that the MESA TWAS results were based on gene expression samples collected from PBMCs, which are closely related to whole-blood gene expression. Among the GTEx tissues, for TC, TG, and LDL, the correlation between MESA-TWAS and GTEx-TWAS for whole blood was stronger than that for any other GTEx tissue, and for HDL, this correlation for whole blood was the third highest among all 49 GTEx tissues. Thus, when the two training data sources MESA and GTEx are compared, the TWAS-TWAS relationships are more consistent than the PWAS-TWAS relationships, with the TWAS-TWAS correlation for whole blood among the strongest. These findings indicate that the heterogeneous relationships between TWAS and PWAS are more attributable to differences in their underlying biological mechanisms and processes than replication issues.

Discussion

In this work, we conducted PWAS for blood lipids and identified 42 proteins significantly associated with at least one of TC, TG, HDL, and LDL. Several of these proteins, such as tyrosine kinase 2 (TYK2),[67,68] MICA and MICB,[69,70] IL-1RN,[42] HP,[42,71] and APOE and APOB,[42,72–74] have been previously identified for their association with blood lipids and related diseases. In particular, we found APOE and APOB to be significantly associated with all four lipid traits. Other proteins, such as lymphotoxin alpha (LTA), C-C motif chemokine ligand 17, and LILRB2, have not been previously identified for their associations with blood lipids.

Moreover, we conducted TWAS for blood lipids in different tissues and compared the results with the PWAS results. We demonstrated that one potential cause of the heterogeneous relationships between the lipid PWAS associations and the lipid TWAS associations is the limited proportion of overlapping SNPs with nonzero predictive weights and their different directions of effect. Nevertheless, when we computed the correlation between the PWAS and TWAS signed log p values for all the genes in every tissue, the correlation coefficients across various tissues were almost all positive. These results demonstrate that for a single gene, its gene expression's association with lipids may differ from its protein's association with lipids, but when the results for all the genes are aggregated, the lipid TWAS and lipid PWAS results are more consistent.

A key component in our association studies is the utilization of imputation-based approaches such as SPrediXcan. This type of method boosts statistical power from two different angles. First, relationships between different omic measurements and phenotypes of interest are easier to detect when the predicted instead of the directly measured omic abundance levels are used. For example, for most genes in MESA, the correlation between the directly measured abundance levels of protein and gene expression is positive but close to zero (Figure S36), but the correlation between the predicted abundance levels is much stronger (Figure S37). Using predictive models is thus advantageous for studying biological traits with complex relationships whose variation originates from multiple sources, such as the gene expression samples and protein samples in our study, where, in the MESA data, the former is collected from PBMCs, while the latter is collected from whole blood and secreted from various organs. This imputation-based approach improves statistical power in the same spirit as existing works that utilize predictive models to mitigate noise in multi-omic data, where the protein-gene expression associations are weak in the raw measurements but more pronounced after noise is reduced by the predictive models.[75,76]

A second advantage of imputation-based approaches in association studies is the utilization of GWAS with large sample sizes. The limited sample size of MESA makes it difficult to detect associations between variables of interest. The overall weak protein-gene expression correlations in MESA are partly attributable to the small sample size, as there are only 699 samples with protein, gene expression, and lipid measurements. However, when we used the MESA samples to train predictive models and applied them to summary statistics from a large GWAS such as the GLGC—which, in the version used in our study, contains 188,577 individuals[16]—the associations between omics and phenotypes became magnified and more noticeable. We note that the statistical power could be

improved further by incorporating more recent GWAS results with an even larger sample size.[9] We leave this for future work.

One limitation of our study is the artifacts of the protein level measurement platform in the PWAS results. The proteomic data are collected using SomaScan, which is an aptamer-based protein-binding assay. It has been known that this platform is known to have cross-activity for protein isoforms.[42,77–79] It is possible that a missense SNP alters the isoform of protein and changes its affinity with the binding aptamers without changing the protein abundance. This could lead to inaccurate association and contribute to the inconsistent association patterns between PWAS and TWAS.

Another limitation of our analyses is that not all confounders of omic or lipid levels might have been accounted for. Blood lipids in GWAS can come from a variety of sources, and there could be factors that are correlated with omic levels but are not included in the study. Similarly, for training the omic prediction models, although we computed the surrogate values to adjust for unobserved factors that are relevant to the analysis, there could still be factors that are not reflected by the surrogate values and other covariates in the model, such as those related to the collection, processing, and storage of blood or plasma, as well as machine artifacts. Furthermore, the set of covariates included in the GWAS might not be the same as those that are adjusted for in the omic prediction models. These potential issues with the covariates and unobserved factors may cause suboptimal accuracy or efficiency in the imputation-based PWAS and TWAS results.

A limitation of our tissue-specific GTEx-based TWAS for lipids is the high number of missing gene-tissue pairs due to their absence in the GTEx data. Imputation methods can be applied to these gene-tissue pairs, so that the missing signed p values of the tissue-specific gene expression-lipid associations could be imputed, which could provide more insight into the connection between the lipid PWAS and the lipid TWAS.

In addition, for training the omic prediction models, samples from all ancestry groups were used to gain power, but in GLGC, most samples are European. This discrepancy in study populations could cause inaccuracy in the analysis.[80–82] A multi-ethnic omic dataset with a larger sample size than MESA will facilitate the training of ancestry-specific, high-power prediction models, and lipid GWAS with more diverse samples will make imputation-based lipid PWAS and lipid TWAS findings more applicable to individuals from non-European populations.[32,83]

Data and code availability

The MESA data are provided by the TOPMed program (https://www.nhlbi.nih.gov/science/trans-omics-precision-medicine-topmed-program). The GLGC GWAS results are available at https://csg.sph.umich.edu/willer/public/lipids2013/. The GTEx TWAS prediction models are available at https://predictdb.org/post/2021/07/21/gtex-v8-models-on-eqtl-and-sqtl/. PredictDBPipeline for training prediction models is available at https://github.com/hakyimlab/PredictDBPipeline. The SPrediXcan software is available at https://github.com/hakyimlab/MetaXcan.

Acknowledgments

This research was supported by NIH grant R01HL142023. Whole-genome sequencing (WGS) for the TOPMed program was supported by the National Heart, Lung, and Blood Institute (NHLBI). WGS for "NHLBI TOPMed: Multi-Ethnic Study of Atherosclerosis (MESA)" (phs001416.v1.p1) was performed at the Broad Institute of MIT and Harvard (3U54HG003067-13S1). Centralized read mapping and genotype calling, along with variant quality metrics and filtering were provided by the TOPMed Informatics Research Center (3R01HL-117626-02S1). Phenotype harmonization, data management, sample-identity quality control, and general study coordination were provided by the TOPMed Data Coordinating Center (3R01HL-120393-02S1) and TOPMed MESA Multi-Omics (HHSN2682015000031/HSN26800004). The MESA projects are conducted and supported by NHLBI in collaboration with MESA investigators. Support for the MESA projects are conducted and supported by NHLBI in collaboration with MESA investigators. Support for MESA is provided by contracts 75N92020D00001, HHSN268201500003I, N01-HC-95159, 75N92020D00005, N01-HC-95160, 75N92020D00002, N01-HC-95161, 75N92020D00003, N01-HC-95162, 75N92020D00006, N01-HC-95163, 75N92020D00004, N01-HC-95164, 75N92020D00007, N01-HC-95165, N01-HC-95166, N01-HC-95167, N01-HC-95168, N01-HC-95169, UL1-TR-000040, UL1-TR-001079, UL1-TR-001420, UL1TR001881, DK063491, and R01HL105756. The authors thank the other investigators, the staff, and the participants of the MESA study for their valuable contributions. A full list of participating MESA investigators and institutes can be found at http://www.mesa-nhlbi.org. S.L. is supported by by the Brain Pool Plus Program through the National Research Foundation of Korea, funded by the Ministry of Science and ICT (2020H1D3A2A03100666). G.M.P. is supported by NIH grants R01HL142711 and R01HL127564. The authors thank Hae Kyung Im and Alvaro Barbeira for their help with using SPrediXcan.

Author contributions

This study was conceived of and led by L.S., X.Z, S.L. D.Z. implemented developed the data processing pipeline, implemented the experiments, and led data analyses with input from L.S., X.Z., S.L. B.G., Q.F. helped with data analyses. The MESA Multi-Omics project was led by S.S.R, J.I.R., with contributions from A.M., G.M.P., R.P.T., P.D., K.D.T., Y.L., W.C.J., S.G., N.G., J.D.S., F.A., K.G.A., T.W.B., R.E.G. The paper was written by D.Z., L.S., X.Z., S.L. with feedback from the other co-authors.

Declaration of interests

The authors declare no competing interests.

Supplemental information

Supplemental information can be found online at https://doi.org/10.1016/j.xhgg.2024.100383.

Web resources

TOPMed program: https://www.nhlbi.nih.gov/science/trans-omics-precision-medicine-topmed-program.

GLGC GWAS results: https://csg.sph.umich.edu/willer/public/lipids2013.

GTEx TWAS prediction models: https://predictdb.org/post/2021/07/21/gtex-v8-models-on-eqtl-and-sqtl/.

PredictDBPipeline: https://github.com/hakyimlab/PredictDBPipeline.

SprediXcan: https://github.com/hakyimlab/MetaXcan.

Received: August 21, 2023
Accepted: November 8, 2024

References

1. Pilia, G., Chen, W.M., Scuteri, A., Orrú, M., Albai, G., Dei, M., Lai, S., Usala, G., Lai, M., Loi, P., et al. (2006). Heritability of Cardiovascular and Personality Traits in 6,148 Sardinians. PLoS Genet. *2*, e132.
2. Willer, C.J., and Mohlke, K.L. (2012). Finding genes and variants for lipid levels after genome-wide association analysis. Curr. Opin. Lipidol. *23*, 98–103.
3. Kannel, W.B., Dawber, T.R., Kagan, A., Revotskie, N., and Stokes, J. (1961). Factors of Risk in the Development of Coronary Heart Disease—Six-Year Follow-up Experience. Ann. Intern. Med. *55*, 33–50.
4. Roger, V.L., Go, A.S., Lloyd-Jones, D.M., Adams, R.J., Berry, J.D., Brown, T.M., Carnethon, M.R., Dai, S., de Simone, G., Ford, E.S., et al. (2011). Heart disease and stroke statistics–2011 update: a report from the American Heart Association. Circulation *123*, e18–e209.
5. Ahmad, F.B., and Anderson, R.N. (2021). The Leading Causes of Death in the US for 2020. JAMA *325*, 1829–1830.
6. Chen, C., Yang, B., Zeng, Z., Yang, H., Liu, C., Ren, J., and Huang, L. (2013). Genetic dissection of blood lipid traits by integrating genome-wide association study and gene expression profiling in a porcine model. BMC Genom. *14*, 848.
7. Hoffmann, T.J., Theusch, E., Haldar, T., Ranatunga, D.K., Jorgenson, E., Medina, M.W., Kvale, M.N., Kwok, P.Y., Schaefer, C., Krauss, R.M., et al. (2018). A large electronic-health-record-based genome-wide study of serum lipids. Nat. Genet. *50*, 401–413.
8. de Vries, P.S., Brown, M.R., Bentley, A.R., Sung, Y.J., Winkler, T.W., Ntalla, I., Schwander, K., Kraja, A.T., Guo, X., Franceschini, N., et al. (2019). Multiancestry Genome-Wide Association Study of Lipid Levels Incorporating Gene-Alcohol Interactions. Am. J. Epidemiol. *188*, 1033–1054.
9. Graham, S.E., Clarke, S.L., Wu, K.H.H., Kanoni, S., Zajac, G.J.M., Ramdas, S., Surakka, I., Ntalla, I., Vedantam, S., Winkler, T.W., et al. (2021). The power of genetic diversity in genome-wide association studies of lipids. Nature *600*, 675–679.
10. Willer, C.J., Sanna, S., Jackson, A.U., Scuteri, A., Bonnycastle, L.L., Clarke, R., Heath, S.C., Timpson, N.J., Najjar, S.S., Stringham, H.M., et al. (2008). Newly identified loci that influence lipid concentrations and risk of coronary artery disease. Nat. Genet. *40*, 161–169.
11. Musunuru, K., Strong, A., Frank-Kamenetsky, M., Lee, N.E., Ahfeldt, T., Sachs, K.V., Li, X., Li, H., Kuperwasser, N., Ruda, V.M., et al. (2010). From noncoding variant to phenotype via SORT1 at the 1p13 cholesterol locus. Nature *466*, 714–719.
12. Burkhardt, R., Toh, S.A., Lagor, W.R., Birkeland, A., Levin, M., Li, X., Robblee, M., Fedorov, V.D., Yamamoto, M., Satoh, T., et al. (2010). Trib1 is a lipid- and myocardial infarction–associated gene that regulates hepatic lipogenesis and VLDL production in mice. J. Clin. Invest. *120*, 4410–4414.
13. Kozlitina, J., Smagris, E., Stender, S., Nordestgaard, B.G., Zhou, H.H., Tybjærg-Hansen, A., Vogt, T.F., Hobbs, H.H., and Cohen, J.C. (2014). Exome-wide association study identifies a TM6SF2 variant that confers susceptibility to nonalcoholic fatty liver disease. Nat. Genet. *46*, 352–356.
14. Manolio, T.A., Collins, F.S., Cox, N.J., Goldstein, D.B., Hindorff, L.A., Hunter, D.J., McCarthy, M.I., Ramos, E.M., Cardon, L.R., Chakravarti, A., et al. (2009). Finding the missing heritability of complex diseases. Nature *461*, 747–753.
15. Kathiresan, S., Manning, A.K., Demissie, S., D'Agostino, R.B., Surti, A., Guiducci, C., Gianniny, L., Burtt, N.P., Melander, O., Orho-Melander, M., et al. (2007). A genome-wide association study for blood lipid phenotypes in the Framingham Heart Study. BMC Med. Genet. *8*, S17.
16. Willer, C.J., Schmidt, E.M., Sengupta, S., Peloso, G.M., Gustafsson, S., Kanoni, S., Ganna, A., Chen, J., Buchkovich, M.L., Mora, S., et al. (2013). Discovery and refinement of loci associated with lipid levels. Nat. Genet. *45*, 1274–1283.
17. Visscher, P.M., Brown, M.A., McCarthy, M.I., and Yang, J. (2012). Five Years of GWAS Discovery. Am. J. Hum. Genet. *90*, 7–24.
18. Wang, S.-B., Feng, J.Y., Ren, W.L., Huang, B., Zhou, L., Wen, Y.J., Zhang, J., Dunwell, J.M., Xu, S., and Zhang, Y.M. (2016). Improving power and accuracy of genome-wide association studies via a multi-locus mixed linear model methodology. Sci. Rep. *6*, 19444.
19. Brandes, N., Linial, N., and Linial, M. (2020). PWAS: proteome-wide association study—linking genes and phenotypes by functional variation in proteins. Genome Biol. *21*, 173.
20. Arneson, D., Shu, L., Tsai, B., Barrere-Cain, R., Sun, C., and Yang, X. (2017). Multidimensional Integrative Genomics Approaches to Dissecting Cardiovascular Disease. Front. Cardiovasc. Med. *4*, 8.
21. Leon-Mimila, P., Wang, J., and Huertas-Vazquez, A. (2019). Relevance of Multi-Omics Studies in Cardiovascular Diseases. Front. Cardiovasc. Med. *6*, 91.
22. Hasin, Y., Seldin, M., and Lusis, A. (2017). Multi-omics approaches to disease. Genome Biol. *18*, 83.
23. Ramazzotti, D., Lal, A., Wang, B., Batzoglou, S., and Sidow, A. (2018). Multi-omic tumor data reveal diversity of molecular mechanisms that correlate with survival. Nat. Commun. *9*, 4453.
24. Xiao, H., Bartoszek, K., and Lio', P. (2018). Multi–omic analysis of signalling factors in inflammatory comorbidities. BMC Bioinf. *19*, 439.
25. Gamazon, E.R., Wheeler, H.E., Shah, K.P., Mozaffari, S.V., Aquino-Michaels, K., Carroll, R.J., Eyler, A.E., Denny, J.C., GTEx Consortium, and Nicolae, D.L., et al. (2015). A gene-based association method for mapping traits using reference transcriptome data. Nat. Genet. *47*, 1091–1098.
26. Gusev, A., Ko, A., Shi, H., Bhatia, G., Chung, W., Penninx, B.W.J.H., Jansen, R., de Geus, E.J.C., Boomsma, D.I., Wright, F.A., et al. (2016). Integrative approaches for large-scale transcriptome-wide association studies. Nat. Genet. *48*, 245–252.
27. Hu, Y., Li, M., Lu, Q., Weng, H., Wang, J., Zekavat, S.M., Yu, Z., Li, B., Gu, J., Muchnik, S., et al. (2019). A statistical framework for cross-tissue transcriptome-wide association analysis. Nat. Genet. *51*, 568–576.

28. Wainberg, M., Sinnott-Armstrong, N., Mancuso, N., Barbeira, A.N., Knowles, D.A., Golan, D., Ermel, R., Ruusalepp, A., Quertermous, T., Hao, K., et al. (2019). Opportunities and challenges for transcriptome-wide association studies. Nat. Genet. *51*, 592–599.
29. Zhu, H., and Zhou, X. (2021). Transcriptome-wide association studies: a view from Mendelian randomization. Quant. Biol. *9*, 107–121. https://doi.org/10.1007/s40484-020-0207-4.
30. Cao, C., Ding, B., Li, Q., Kwok, D., Wu, J., and Long, Q. (2021). Power analysis of transcriptome-wide association study: Implications for practical protocol choice. PLoS Genet. *17*, e1009405.
31. Gusev, A., Mancuso, N., Won, H., Kousi, M., Finucane, H.K., Reshef, Y., Song, L., Safi, A., Schizophrenia Working Group of the Psychiatric Genomics Consortium, and McCarroll, S., et al. (2018). Transcriptome-wide association study of schizophrenia and chromatin activity yields mechanistic disease insights. Nat. Genet. *50*, 538–548.
32. Bhattacharya, A., García-Closas, M., Olshan, A.F., Perou, C.M., Troester, M.A., and Love, M.I. (2020). A framework for transcriptome-wide association studies in breast cancer in diverse study populations. Genome Biol. *21*, 42.
33. Zhao, B., Shan, Y., Yang, Y., Yu, Z., Li, T., Wang, X., Luo, T., Zhu, Z., Sullivan, P., Zhao, H., et al. (2021). Transcriptome-wide association analysis of brain structures yields insights into pleiotropy with complex neuropsychiatric traits. Nat. Commun. *12*, 2878.
34. Wingo, A.P., Liu, Y., Gerasimov, E.S., Gockley, J., Logsdon, B.A., Duong, D.M., Dammer, E.B., Robins, C., Beach, T.G., Reiman, E.M., et al. (2021). Integrating human brain proteomes with genome-wide association data implicates new proteins in Alzheimer's disease pathogenesis. Nat. Genet. *53*, 143–146.
35. Wu, B.-S., Chen, S.F., Huang, S.Y., Ou, Y.N., Deng, Y.T., Chen, S.D., Dong, Q., and Yu, J.T. (2022). Identifying causal genes for stroke via integrating the proteome and transcriptome from brain and blood. J. Transl. Med. *20*, 181.
36. Wingo, T.S., Liu, Y., Gerasimov, E.S., Gockley, J., Logsdon, B.A., Duong, D.M., Dammer, E.B., Lori, A., Kim, P.J., Ressler, K.J., et al. (2021). Brain proteome-wide association study implicates novel proteins in depression pathogenesis. Nat. Neurosci. *24*, 810–817.
37. Wingo, T.S., Gerasimov, E.S., Liu, Y., Duong, D.M., Vattathil, S.M., Lori, A., Gockley, J., Breen, M.S., Maihofer, A.X., Nievergelt, C.M., et al. (2022). Integrating human brain proteomes with genome-wide association data implicates novel proteins in post-traumatic stress disorder. Mol. Psychiatr. *27*, 3075–3084.
38. Liu, J., Li, X., and Luo, X.-J. (2021). Proteome-wide Association Study Provides Insights Into the Genetic Component of Protein Abundance in Psychiatric Disorders. Biol. Psychiatr. *90*, 781–789.
39. Veturi, Y., Lucas, A., Bradford, Y., Hui, D., Dudek, S., Theusch, E., Verma, A., Miller, J.E., Kullo, I., Hakonarson, H., et al. (2021). A unified framework identifies new links between plasma lipids and diseases from electronic medical records across large-scale cohorts. Nat. Genet. *53*, 972–981.
40. Feng, H., Mancuso, N., Pasaniuc, B., and Kraft, P. (2021). Multitrait transcriptome-wide association study (TWAS) tests. Genet. Epidemiol. *45*, 563–576.
41. Yang, T., Wu, C., Wei, P., and Pan, W. (2020). Integrating DNA sequencing and transcriptomic data for association analyses of low-frequency variants and lipid traits. Hum. Mol. Genet. *29*, 515–526.
42. Schubert, R., Geoffroy, E., Gregga, I., Mulford, A.J., Aguet, F., Ardlie, K., Gerszten, R., Clish, C., Van Den Berg, D., Taylor, K.D., et al. (2022). Protein prediction for trait mapping in diverse populations. PLoS One *17*, e0264341.
43. Bild, D.E., Bluemke, D.A., Burke, G.L., Detrano, R., Diez Roux, A.V., Folsom, A.R., Greenland, P., Jacob, D.R., Jr., Kronmal, R., Liu, K., et al. (2002). Multi-Ethnic Study of Atherosclerosis: Objectives and Design. Am. J. Epidemiol. *156*, 871–881.
44. Burke, G., Lima, J., Wong, N.D., and Narula, J. (2016). The Multiethnic Study of Atherosclerosis. Glob. Heart *11*, 267–268.
45. Lonsdale, J., Thomas, J., Salvatore, M., Phillips, R., Lo, E., Shad, S., Hasz, R., Walters, G., Garcia, F., Young, N., et al. (2013). The Genotype-Tissue Expression (GTEx) project. Nat. Genet. *45*, 580–585.
46. Taliun, D., Harris, D.N., Kessler, M.D., Carlson, J., Szpiech, Z.A., Torres, R., Taliun, S.A.G., Corvelo, A., Gogarten, S.M., Kang, H.M., et al. (2021). Sequencing of 53,831 diverse genomes from the NHLBI TOPMed Program. Nature *590*, 290–299.
47. Kowalski, M.H., Qian, H., Hou, Z., Rosen, J.D., Tapia, A.L., Shan, Y., Jain, D., Argos, M., Arnett, D.K., Avery, C., et al. (2019). Use of >100,000 NHLBI Trans-Omics for Precision Medicine (TOPMed) Consortium whole genome sequences improves imputation quality and detection of rare variant associations in admixed African and Hispanic/Latino populations. PLoS Genet. *15*, e1008500.
48. Manichaikul, A., Mychaleckyj, J.C., Rich, S.S., Daly, K., Sale, M., and Chen, W.M. (2010). Robust relationship inference in genome-wide association studies. Bioinformatics *26*, 2867–2873.
49. Gold, L., Ayers, D., Bertino, J., Bock, C., Bock, A., Brody, E.N., Carter, J., Dalby, A.B., Eaton, B.E., Fitzwater, T., et al. (2010). Aptamer-Based Multiplexed Proteomic Technology for Biomarker Discovery. PLoS One *5*, e15004.
50. Raffield, L.M., Dang, H., Pratte, K.A., Jacobson, S., Gillenwater, L.A., Ampleford, E., Barjaktarevic, I., Basta, P., Clish, C.B., Comellas, A.P., et al. (2020). Comparison of Proteomic Assessment Methods in Multiple Cohort Studies. Proteomics *20*, 1900278.
51. Lee, S., Sun, W., Wright, F.A., and Zou, F. (2017). An improved and explicit surrogate variable analysis procedure by coefficient adjustment. Biometrika *104*, 303–316.
52. Brown, K.M., Diez-Roux, A.V., Smith, J.A., Needham, B.L., Mukherjee, B., Ware, E.B., Liu, Y., Cole, S.W., Seeman, T.E., and Kardia, S.L.R. (2019). Expression of socially sensitive genes: The multi-ethnic study of atherosclerosis. PLoS One *14*, e0214061.
53. Liu, Y., Ding, J., Reynolds, L.M., Lohman, K., Register, T.C., De La Fuente, A., Howard, T.D., Hawkins, G.A., Cui, W., Morris, J., et al. (2013). Methylomics of gene expression in human monocytes. Hum. Mol. Genet. *22*, 5065–5074.
54. Barbeira, A.N., Dickinson, S.P., Bonazzola, R., Zheng, J., Wheeler, H.E., Torres, J.M., Torstenson, E.S., Shah, K.P., Garcia, T., Edwards, T.L., et al. (2018). Exploring the phenotypic consequences of tissue specific gene expression variation inferred from GWAS summary statistics. Nat. Commun. *9*, 1825.
55. Zou, H., and Hastie, T. (2005). Regularization and variable selection via the elastic net. J. R. Stat. Soc. Ser. B Stat. Methodol. *67*, 301–320.
56. PredictDB Team (2021). GTEx v8 models on eQTL and sQTL. https://predictdb.org/post/2021/07/21/gtex-v8-models-on-eqtl-and-sqtl/.

57. Haky Im Lab (2013). PredictDBPipeline. GitHub repository. https://github.com/charlespwd/project-title.
58. Ferreira, J.A., and Zwinderman, A.H. (2006). On the Benjamini–Hochberg method. Ann. Stat. *34*, 1827–1849.
59. Myers, L., and Sirois, M.J. (2006). Spearman Correlation Coefficients, Differences between. In Encyclopedia of Statistical Sciences (American Cancer Society). https://doi.org/10.1002/0471667196.ess5050.pub2.
60. Zhen, J., Huang, X., Van Halm-Lutterodt, N., Dong, S., Ma, W., Xiao, R., and Yuan, L. (2017). ApoE rs429358 and rs7412 Polymorphism and Gender Differences of Serum Lipid Profile and Cognition in Aging Chinese Population. Front. Aging Neurosci. *9*, 248.
61. Wu, H., Huang, Q., Yu, Z., Wu, H., and Zhong, Z. (2020). The SNPs rs429358 and rs7412 of APOE gene are association with cerebral infarction but not SNPs rs2306283 and rs4149056 of SLCO1B1 gene in southern Chinese Hakka population. Lipids Health Dis. *19*, 202.
62. Clément-Collin, V., Barbier, A., Dergunov, A.D., Visvikis, A., Siest, G., Desmadril, M., Takahashi, M., and Aggerbeck, L.P. (2006). The structure of human apolipoprotein E2, E3 and E4 in solution. 2. Multidomain organization correlates with the stability of apoE structure. Biophys. Chem. *119*, 170–185.
63. Tejedor, M.T., Garcia-Sobreviela, M.P., Ledesma, M., and Arbones-Mainar, J.M. (2014). The Apolipoprotein E Polymorphism rs7412 Associates with Body Fatness Independently of Plasma Lipids in Middle Aged Men. PLoS One *9*, e108605.
64. Huang, Y., Ye, H.D., Gao, X., Nie, S., Hong, Q.X., Ji, H.H., Sun, J., Zhou, S.J., Fei, B., Li, K.Q., et al. (2015). Significant interaction of APOE rs4420638 polymorphism with HDL-C and APOA-I levels in coronary heart disease in Han Chinese men. Genet. Mol. Res. *14*, 13414–13424.
65. Giambartolomei, C., Vukcevic, D., Schadt, E.E., Franke, L., Hingorani, A.D., Wallace, C., and Plagnol, V. (2014). Bayesian Test for Colocalisation between Pairs of Genetic Association Studies Using Summary Statistics. PLoS Genet. *10*, e1004383.
66. Zuber, V., Grinberg, N.F., Gill, D., Manipur, I., Slob, E.A.W., Patel, A., Wallace, C., and Burgess, S. (2022). Combining evidence from Mendelian randomization and colocalization: Review and comparison of approaches. Am. J. Hum. Genet. *109*, 767–782.
67. Qi, W., Zhou, L., Zhao, T., Ding, S., Xu, Q., Han, X., Zhao, Y., Song, X., Zhao, T., Zhang, X., and Ye, L. (2019). Effect of the TYK-2/STAT-3 pathway on lipid accumulation induced by mono-2-ethylhexyl phthalate. Mol. Cell. Endocrinol. *484*, 52–58.
68. Grunert, T., Leitner, N.R., Marchetti-Deschmann, M., Miller, I., Wallner, B., Radwan, M., Vogl, C., Kolbe, T., Kratky, D., Gemeiner, M., et al. (2011). A comparative proteome analysis links tyrosine kinase 2 (Tyk2) to the regulation of cellular glucose and lipid metabolism in response to poly(I:C). J. Proteonomics *74*, 2866–2880.
69. Bilotta, M.T., Abruzzese, M.P., Molfetta, R., Scarno, G., Fionda, C., Zingoni, A., Soriani, A., Garofalo, T., Petrucci, M.T., Ricciardi, M.R., et al. (2019). Activation of liver X receptor up-regulates the expression of the NKG2D ligands MICA and MICB in multiple myeloma through different molecular mechanisms. Faseb. J. *33*, 9489–9504.
70. Yamamoto, K., Fujiyama, Y., Andoh, A., Bamba, T., and Okabe, H. (2001). Oxidative stress increases MICA and MICB gene expression in the human colon carcinoma cell line (CaCo-2). Biochim. Biophys. Acta *1526*, 10–12.
71. Braeckman, L., De Bacquer, D., Delanghe, J., Claeys, L., and De Backer, G. (1999). Associations between haptoglobin polymorphism, lipids, lipoproteins and inflammatory variables. Atherosclerosis *143*, 383–388.
72. Weisgraber, K.H. (1994). Apolipoprotein E: Structure-Function Relationships. In Advances in Protein Chemistry, *45*, C.B. Anfinsen, J.T. Edsall, F.M. Richards, and D.S. Eisenberg, eds. *(Academic Press), pp. 249–302*.
73. Emerging Risk Factors Collaboration, Di Angelantonio, E., Sarwar, N., Perry, P., Kaptoge, S., Ray, K.K., Thompson, A., Wood, A.M., Lewington, S., Sattar, N., et al. (2009). Major Lipids, Apolipoproteins, and Risk of Vascular Disease. JAMA *302*, 1993–2000.
74. Abd El-Aziz, T.A., and Mohamed, R.H. (2016). LDLR, ApoB and ApoE genes polymorphisms and classical risk factors in premature coronary artery disease. Gene *590*, 263–269.
75. Zhou, Z., Ye, C., Wang, J., and Zhang, N.R. (2020). Surface protein imputation from single cell transcriptomes by deep neural networks. Nat. Commun. *11*, 651.
76. Lakkis, J., Schroeder, A., Su, K., Lee, M.Y.Y., Bashore, A.C., Reilly, M.P., and Li, M. (2022). A multi-use deep learning method for CITE-seq and single-cell RNA-seq data integration with cell surface protein prediction and imputation. Nat. Mach. Intell. *4*, 940–952.
77. Joshi, A., and Mayr, M. (2018). In Aptamers They Trust. Circulation *138*, 2482–2485.
78. Mosley, J.D., Benson, M.D., Smith, J.G., Melander, O., Ngo, D., Shaffer, C.M., Ferguson, J.F., Herzig, M.S., McCarty, C.A., Chute, C.G., et al. (2018). Probing the Virtual Proteome to Identify Novel Disease Biomarkers. Circulation *138*, 2469–2481.
79. Sun, B.B., Maranville, J.C., Peters, J.E., Stacey, D., Staley, J.R., Blackshaw, J., Burgess, S., Jiang, T., Paige, E., Surendran, P., et al. (2018). Genomic atlas of the human plasma proteome. Nature *558*, 73–79.
80. Abdellaoui, A., Hugh-Jones, D., Yengo, L., Kemper, K.E., Nivard, M.G., Veul, L., Holtz, Y., Zietsch, B.P., Frayling, T.M., Wray, N.R., et al. (2019). Genetic correlates of social stratification in Great Britain. Nat. Human Behav. *3*, 1332–1342.
81. Price, A.L., Zaitlen, N.A., Reich, D., and Patterson, N. (2010). New approaches to population stratification in genome-wide association studies. Nat. Rev. Genet. *11*, 459–463.
82. Zhang, D., Dey, R., and Lee, S. (2020). Fast and robust ancestry prediction using principal component analysis. Bioinformatics *36*, 3439–3446.
83. Keys, K.L., Mak, A.C.Y., White, M.J., Eckalbar, W.L., Dahl, A.W., Mefford, J., Mikhaylova, A.V., Contreras, M.G., Elhawary, J.R., Eng, C., et al. (2020). On the cross-population generalizability of gene expression prediction models. PLoS Genet. *16*, e1008927.

HGGA, Volume *6*

Supplemental information

Proteome-wide association studies for blood lipids and comparison with transcriptome-wide association studies

Daiwei Zhang, Boran Gao, Qidi Feng, Ani Manichaikul, Gina M. Peloso, Russell P. Tracy, Peter Durda, Kent D. Taylor, Yongmei Liu, W. Craig Johnson, Stacey Gabriel, Namrata Gupta, Joshua D. Smith, Francois Aguet, Kristin G. Ardlie, Thomas W. Blackwell, Robert E. Gerszten, Stephen S. Rich, Jerome I. Rotter, Laura J. Scott, Xiang Zhou, and Seunggeun Lee

Supplementary Materials

S1 Additional results for all lipids

Figure S1: MESA protein prediction model performance.

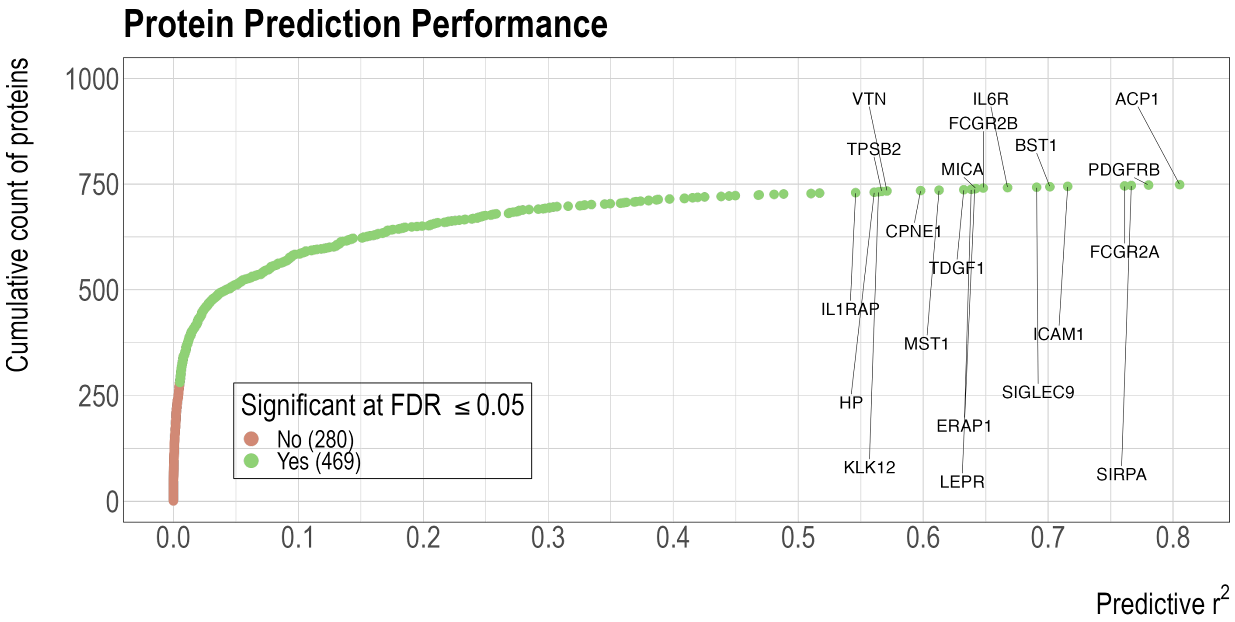

Figure S2: Comparison of APOE's protein and gene expression predictive model weights with the LDL GWAS z-scores of the SNPs. The reference and alternative alleles for GWAS and the predictive models have been aligned and reordered so that all the SNPs have positive GWAS effects. The z-scores are used to compute the weighted average of the model weights (dashed lines), which have the same signs as and are proportional to the predicted effects of protein and gene expression on the GWAS outcome.

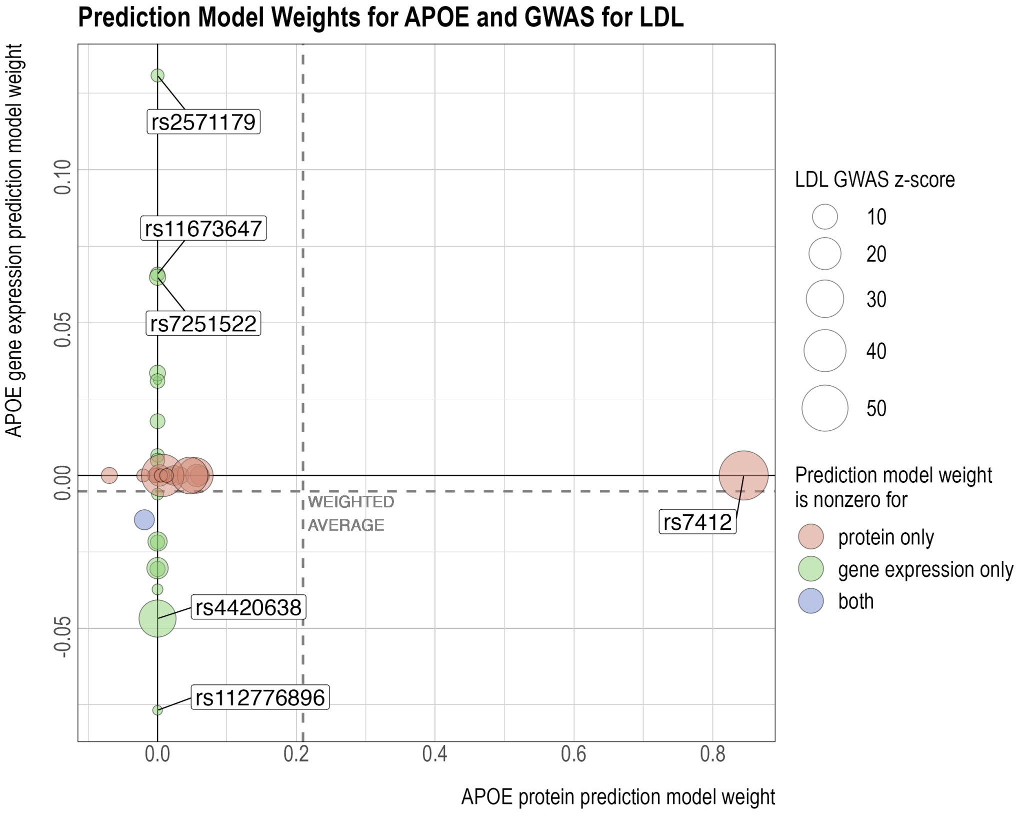

Figure S3: GWAS for LDL and prediction models for FCGR2B's protein and gene expression levels. The reference and alternative alleles for GWAS and the predictive models have been aligned and reordered so that all the SNPs have positive GWAS effects. In the center and bottom panels, the size of the circles indicates the SNP's GWAS z-score. The z-scores are used to compute the weighted average of the model weights (dashed line), which has the same sign as and is proportional to the predicted effect of protein or gene expression on the GWAS outcome.

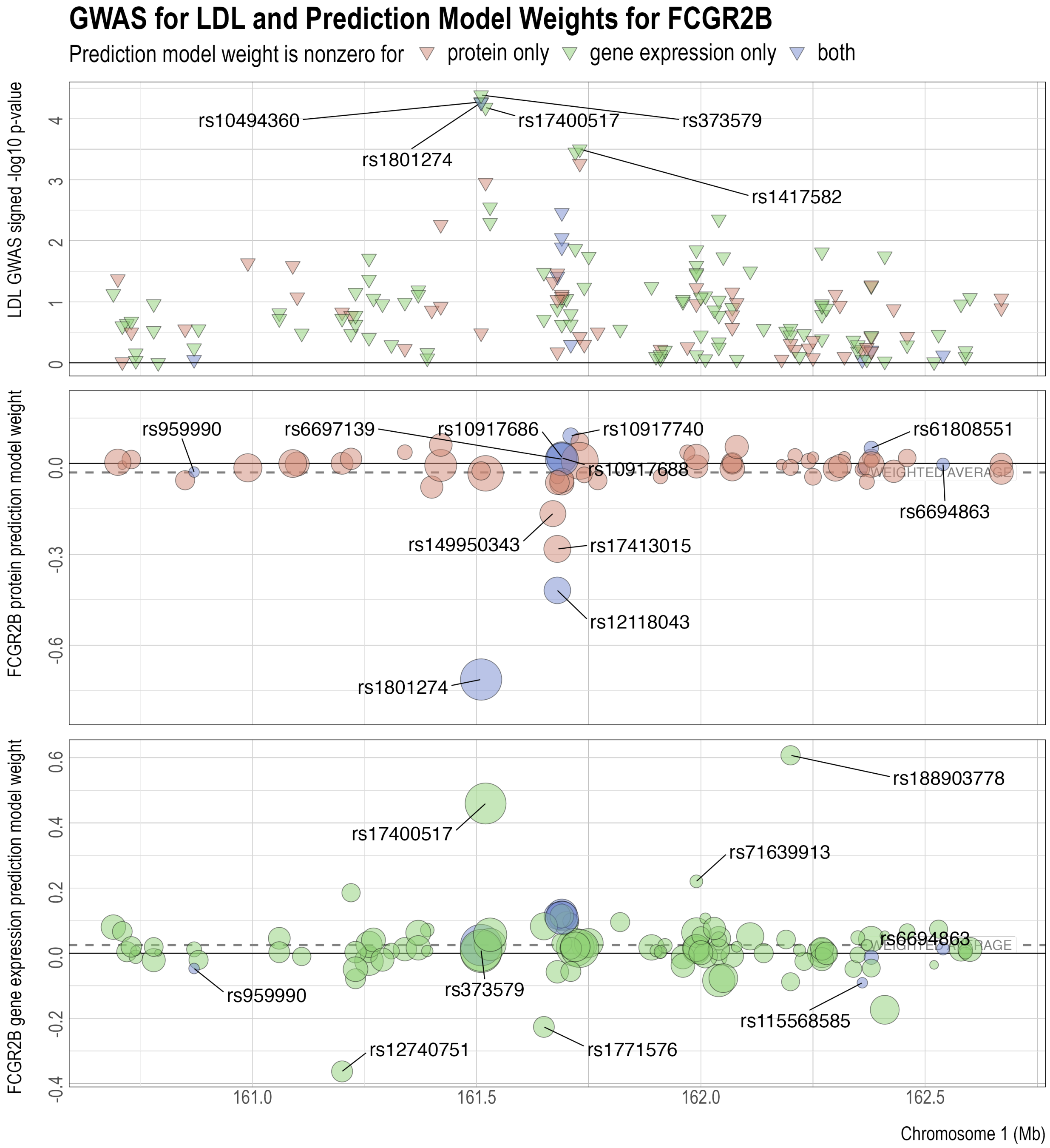

Figure S4: Comparison of FCGR2B's protein and gene expression predictive model weights with the LDL GWAS z-scores of the SNPs. The reference and alternative alleles for GWAS and the predictive models have been aligned and reordered so that all the SNPs have positive GWAS effects. The z-scores are used to compute the weighted average of the model weights (dashed lines), which have the same signs as and are proportional to the predicted effects of protein and gene expression on the GWAS outcome.

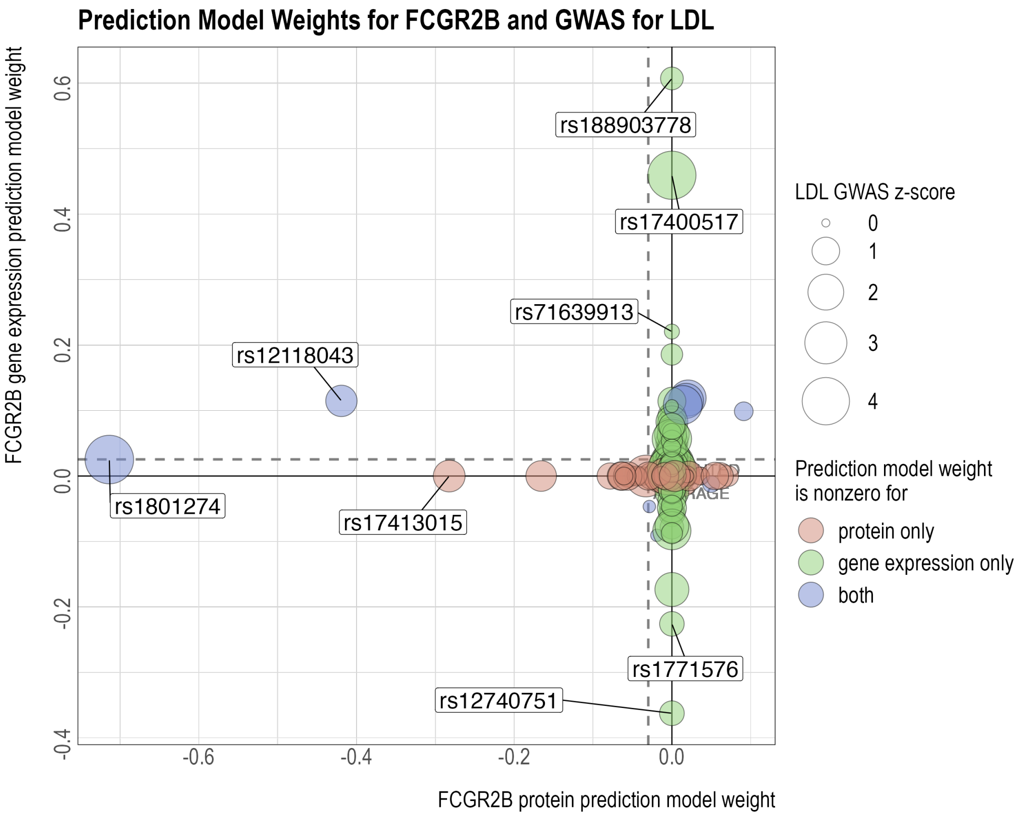

Figure S5: GWAS for LDL and prediction models for LILRB2's protein and gene expression levels. The reference and alternative alleles for GWAS and the predictive models have been aligned and reordered so that all the SNPs have positive GWAS effects. In the center and bottom panels, the size of the circles indicates the SNP's GWAS z-score. The z-scores are used to compute the weighted average of the model weights (dashed line), which has the same sign as and is proportional to the predicted effect of protein or gene expression on the GWAS outcome.

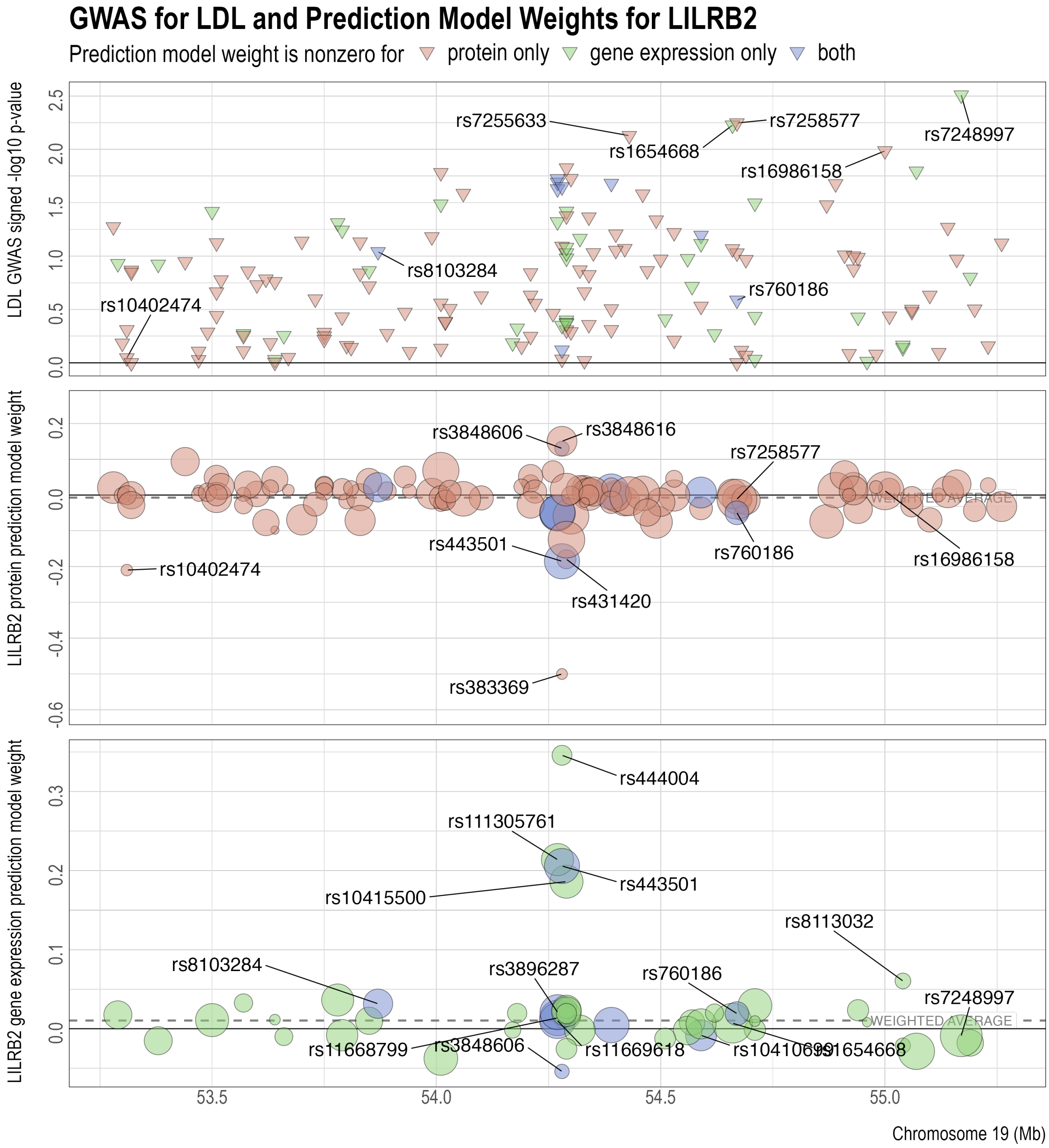

Figure S6: Comparison of LILRB2's protein and gene expression predictive model weights with the LDL GWAS z-scores of the SNPs. The reference and alternative alleles for GWAS and the predictive models have been aligned and reordered so that all the SNPs have positive GWAS effects. The z-scores are used to compute the weighted average of the model weights (dashed lines), which have the same signs as and are proportional to the predicted effects of protein and gene expression on the GWAS outcome.

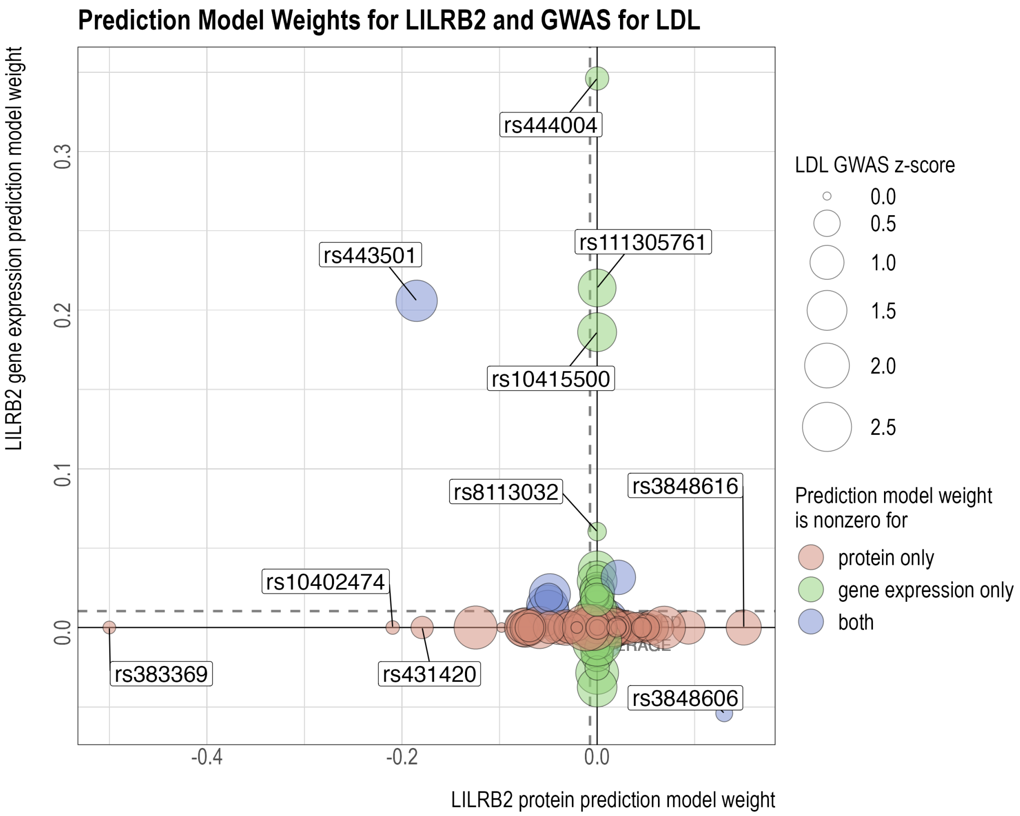

Figure S7: GWAS for LDL and prediction models for MICB's protein and gene expression levels. The reference and alternative alleles for GWAS and the predictive models have been aligned and reordered so that all the SNPs have positive GWAS effects. In the center and bottom panels, the size of the circles indicates the SNP's GWAS z-score. The z-scores are used to compute the weighted average of the model weights (dashed line), which has the same sign as and is proportional to the predicted effect of protein or gene expression on the GWAS outcome.

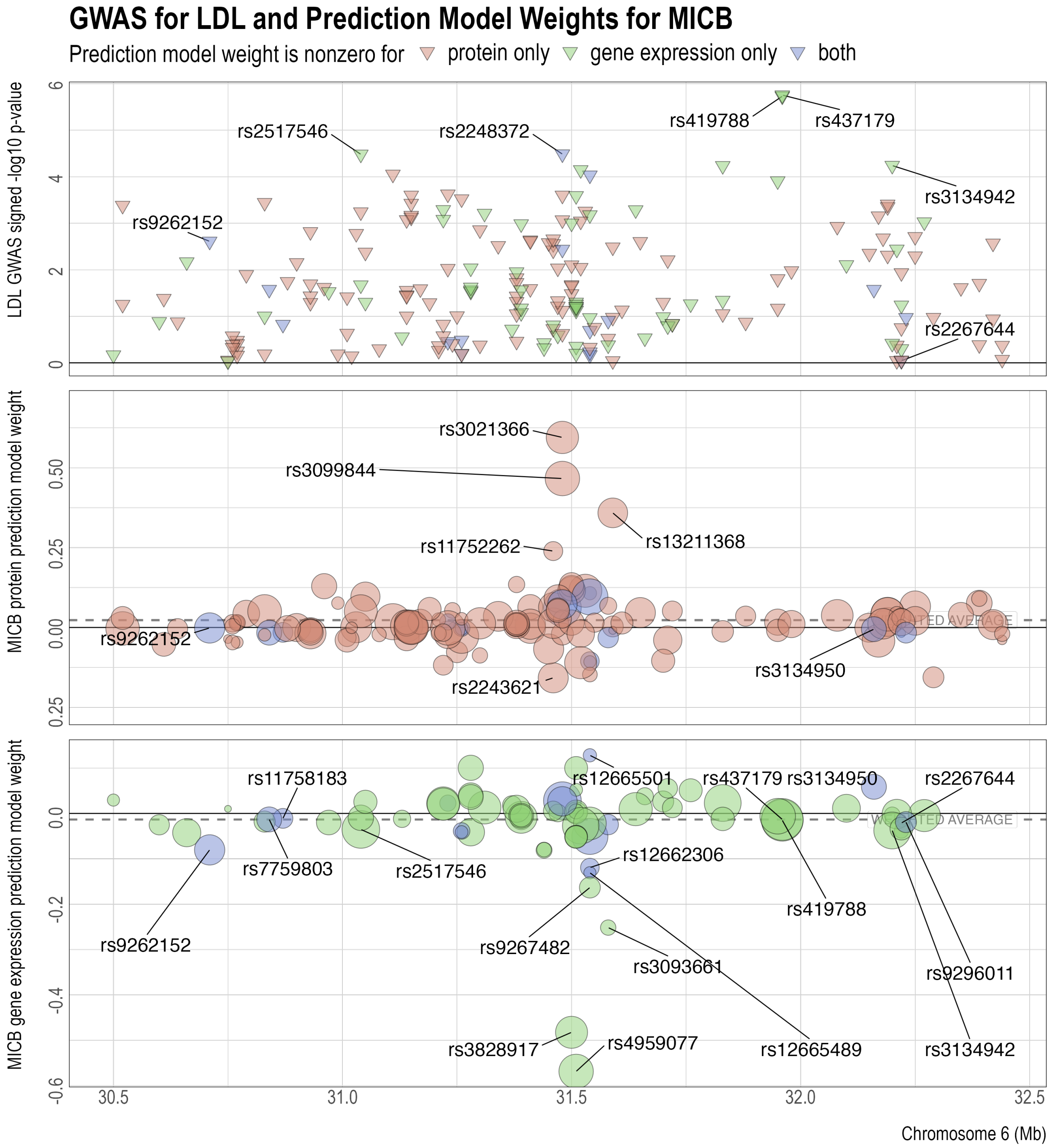

Figure S8: Comparison of MICB's protein and gene expression predictive model weights with the LDL GWAS z-scores of the SNPs. The reference and alternative alleles for GWAS and the predictive models have been aligned and reordered so that all the SNPs have positive GWAS effects. The z-scores are used to compute the weighted average of the model weights (dashed lines), which have the same signs as and are proportional to the predicted effects of protein and gene expression on the GWAS outcome.

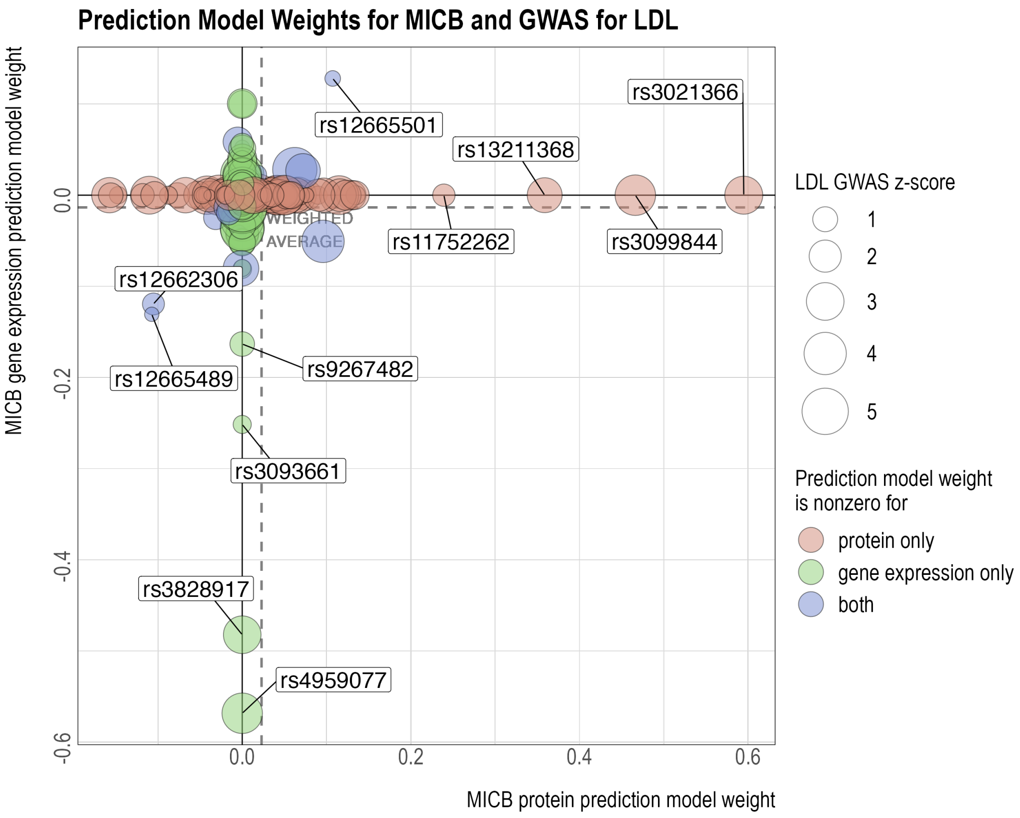

Figure S9: GWAS for TC and prediction models for APOE's protein and gene expression levels. The reference and alternative alleles for GWAS and the predictive models have been aligned and reordered so that all the SNPs have positive GWAS effects. In the center and bottom panels, the size of the circles indicates the SNP's GWAS z-score. The z-scores are used to compute the weighted average of the model weights (dashed line), which has the same sign as and is proportional to the predicted effect of protein or gene expression on the GWAS outcome.

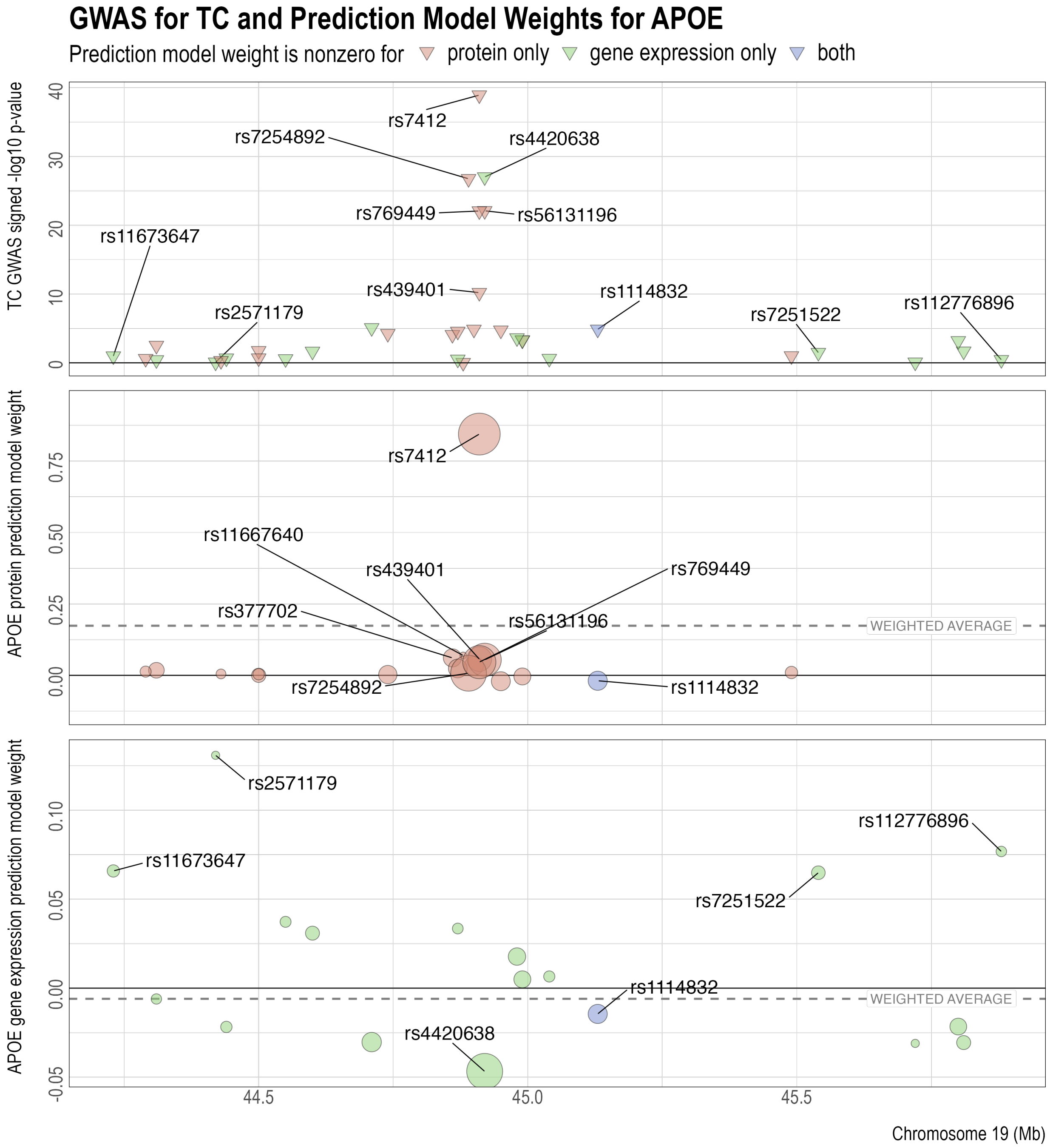

Figure S10: Comparison of APOE's protein and gene expression predictive model weights with the TC GWAS z-scores of the SNPs. The reference and alternative alleles for GWAS and the predictive models have been aligned and reordered so that all the SNPs have positive GWAS effects. The z-scores are used to compute the weighted average of the model weights (dashed lines), which have the same signs as and are proportional to the predicted effects of protein and gene expression on the GWAS outcome.

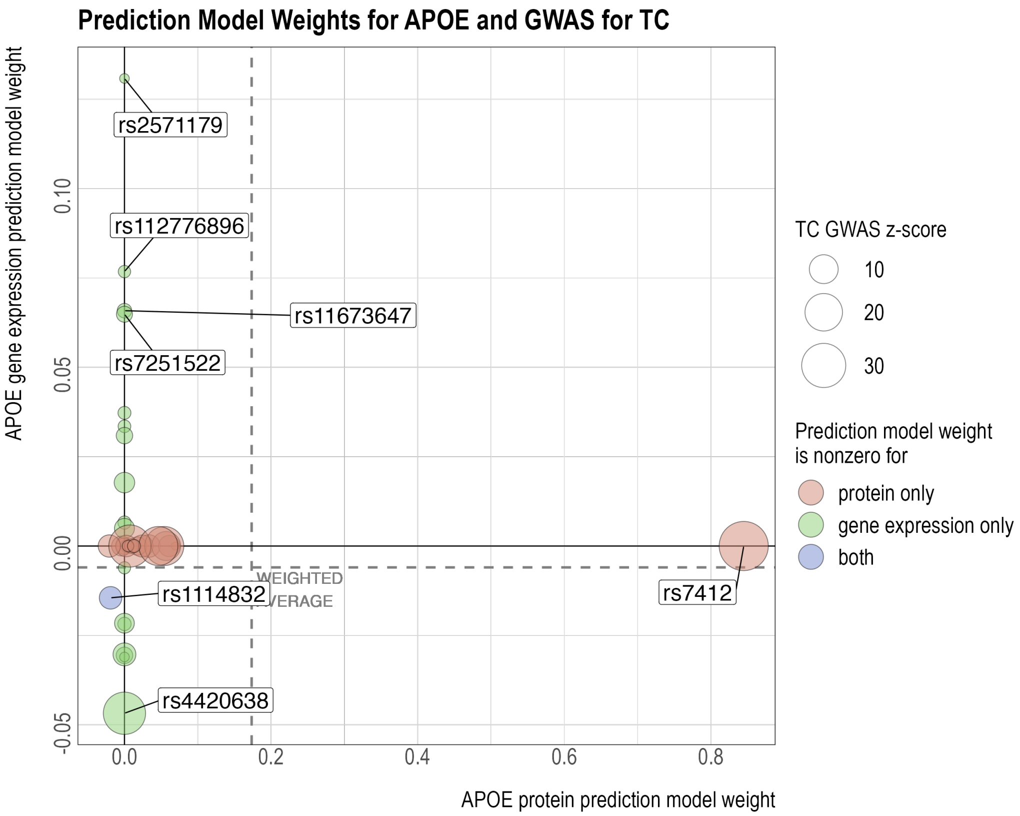

Figure S11: GWAS for TC and prediction models for FCGR2B's protein and gene expression levels. The reference and alternative alleles for GWAS and the predictive models have been aligned and reordered so that all the SNPs have positive GWAS effects. In the center and bottom panels, the size of the circles indicates the SNP's GWAS z-score. The z-scores are used to compute the weighted average of the model weights (dashed line), which has the same sign as and is proportional to the predicted effect of protein or gene expression on the GWAS outcome.

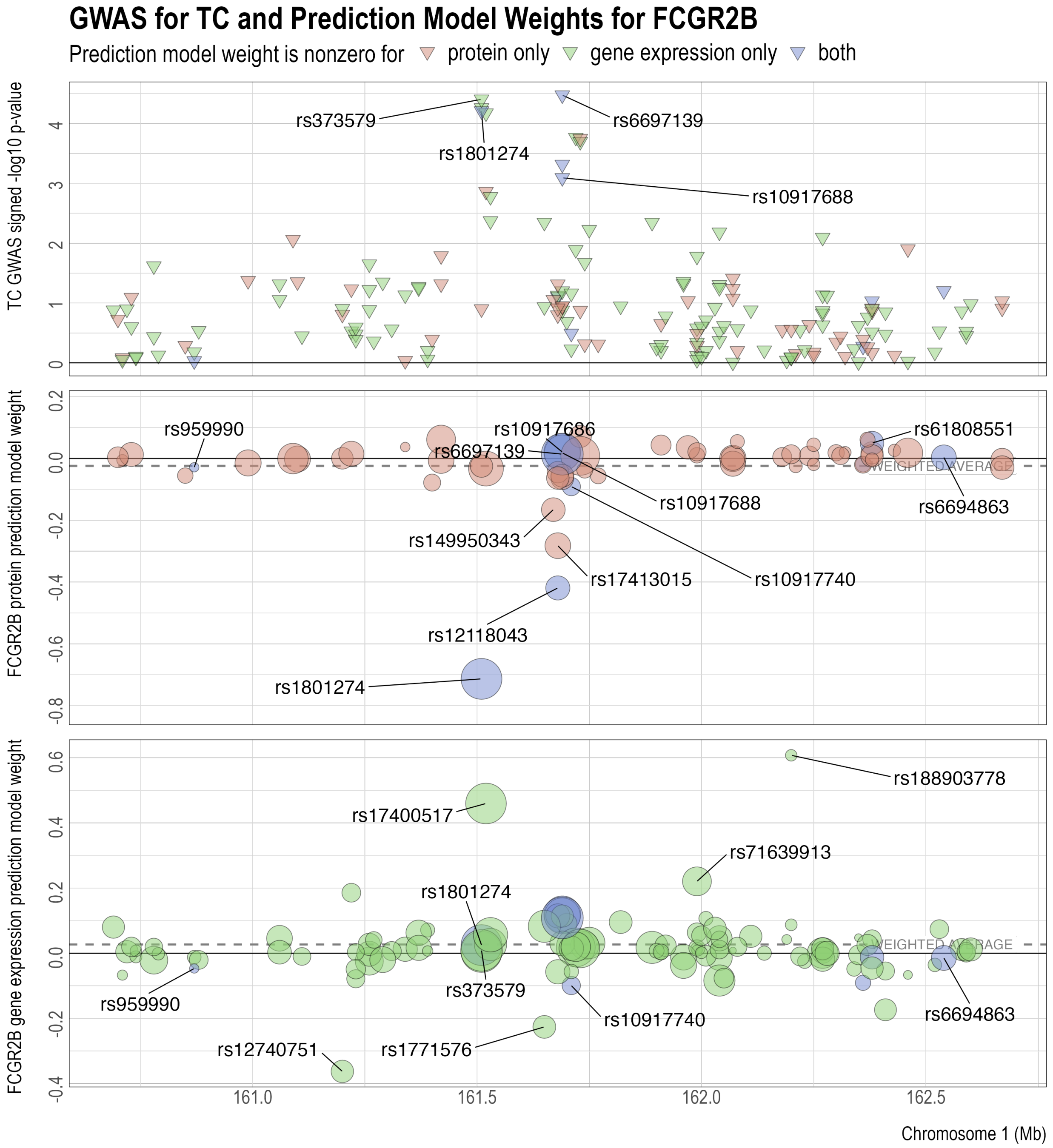

Figure S12: Comparison of FCGR2B's protein and gene expression predictive model weights with the TC GWAS z-scores of the SNPs. The reference and alternative alleles for GWAS and the predictive models have been aligned and reordered so that all the SNPs have positive GWAS effects. The z-scores are used to compute the weighted average of the model weights (dashed lines), which have the same signs as and are proportional to the predicted effects of protein and gene expression on the GWAS outcome.

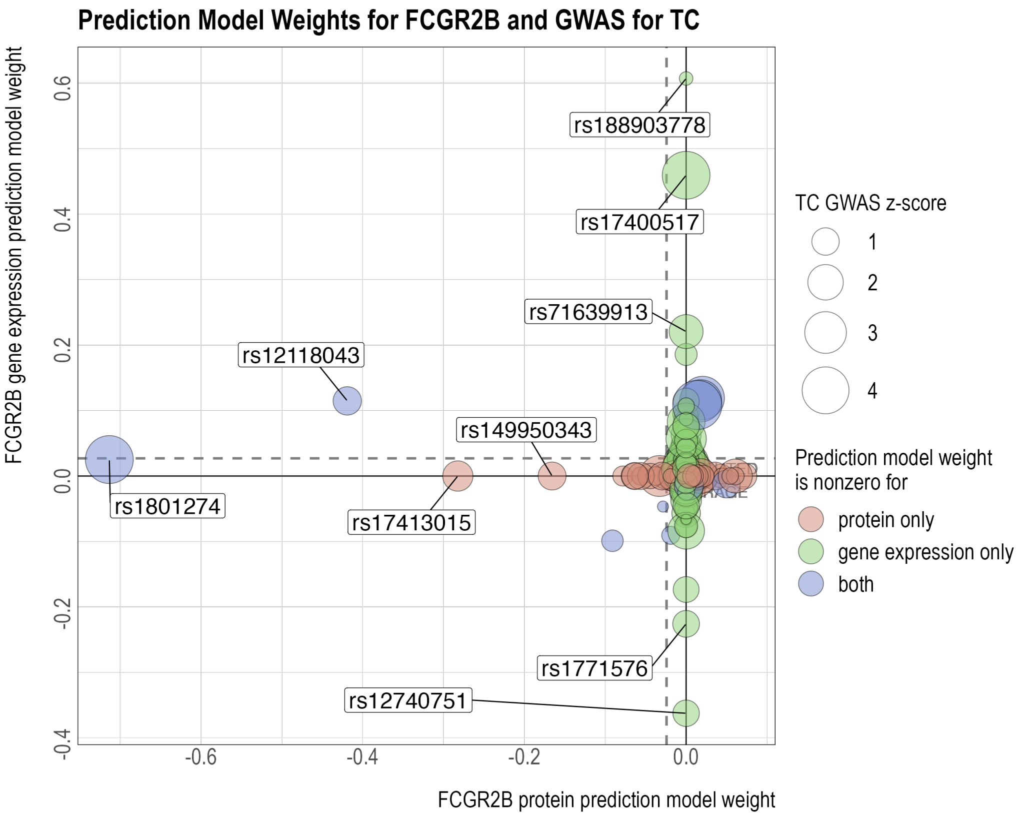

Figure S13: GWAS for TC and prediction models for LILRB2's protein and gene expression levels. The reference and alternative alleles for GWAS and the predictive models have been aligned and reordered so that all the SNPs have positive GWAS effects. In the center and bottom panels, the size of the circles indicates the SNP's GWAS z-score. The z-scores are used to compute the weighted average of the model weights (dashed line), which has the same sign as and is proportional to the predicted effect of protein or gene expression on the GWAS outcome.

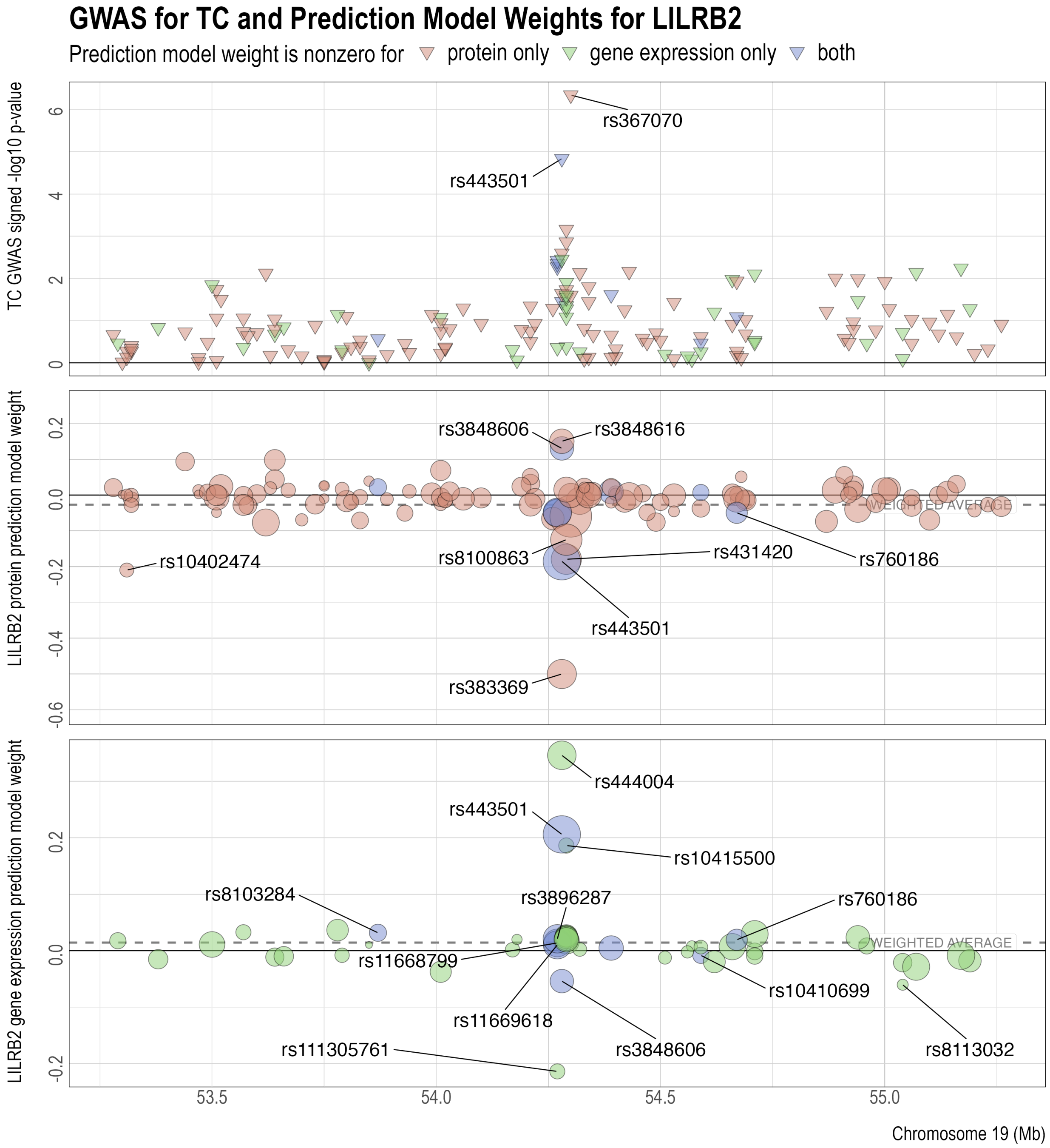

Figure S14: Comparison of LILRB2's protein and gene expression predictive model weights with the TC GWAS z-scores of the SNPs. The reference and alternative alleles for GWAS and the predictive models have been aligned and reordered so that all the SNPs have positive GWAS effects. The z-scores are used to compute the weighted average of the model weights (dashed lines), which have the same signs as and are proportional to the predicted effects of protein and gene expression on the GWAS outcome.

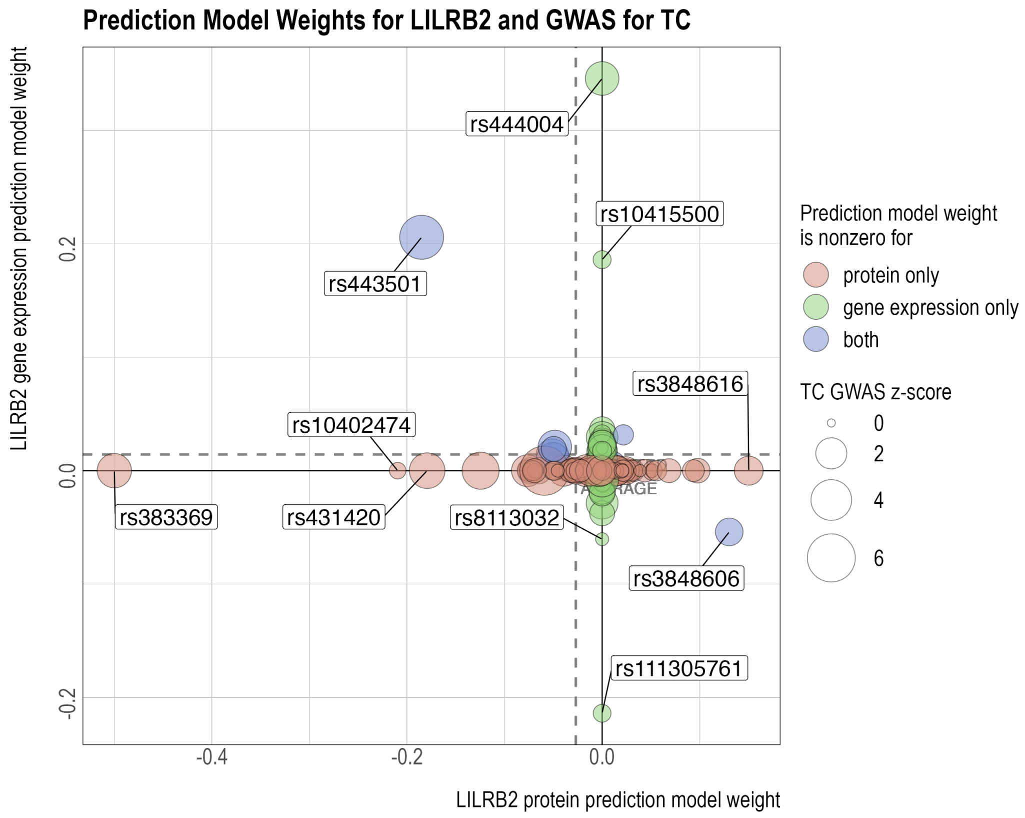

Figure S15: GWAS for TC and prediction models for MICB's protein and gene expression levels. The reference and alternative alleles for GWAS and the predictive models have been aligned and reordered so that all the SNPs have positive GWAS effects. In the center and bottom panels, the size of the circles indicates the SNP's GWAS z-score. The z-scores are used to compute the weighted average of the model weights (dashed line), which has the same sign as and is proportional to the predicted effect of protein or gene expression on the GWAS outcome.

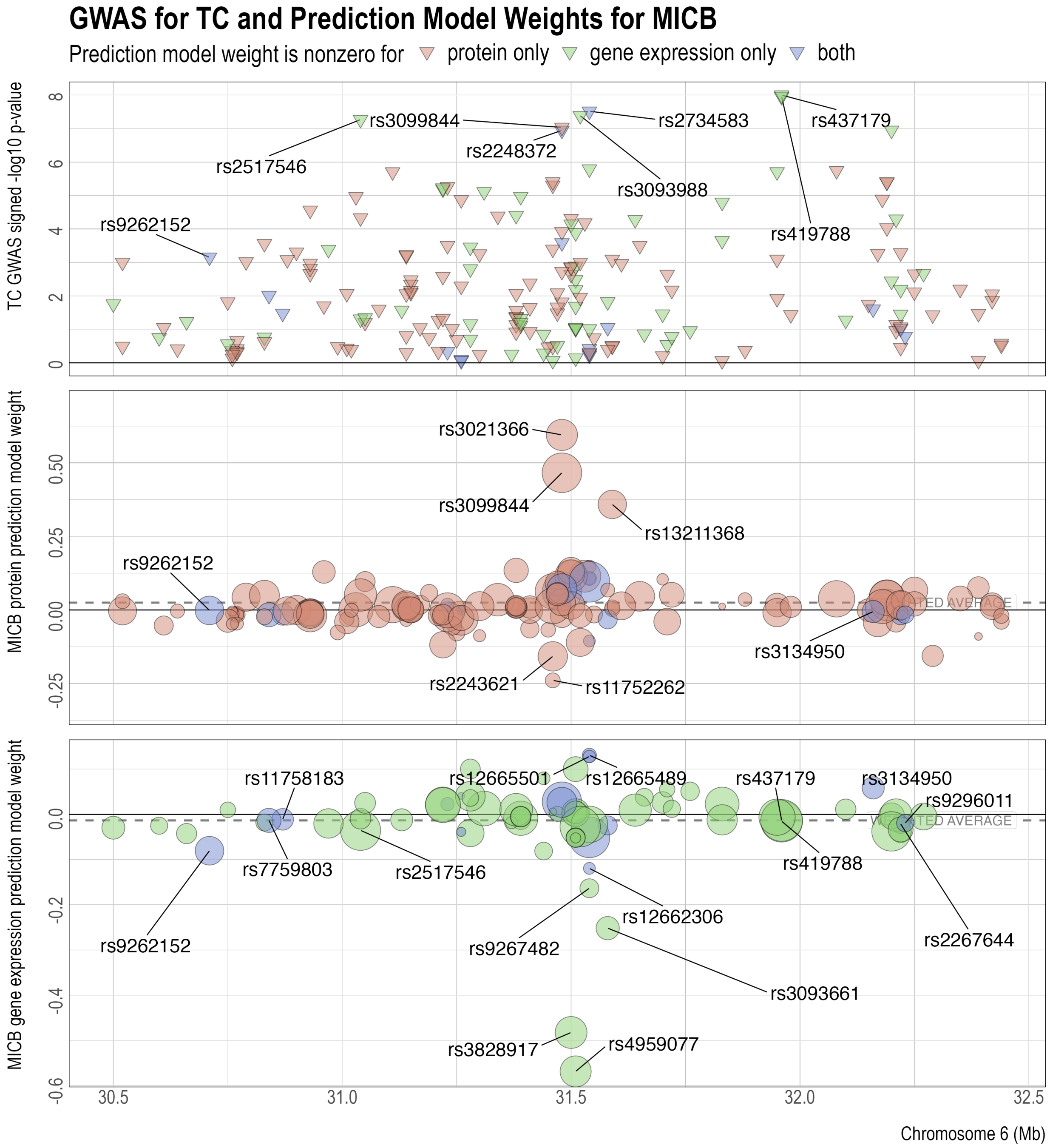

Figure S16: Comparison of MICB's protein and gene expression predictive model weights with the TC GWAS z-scores of the SNPs. The reference and alternative alleles for GWAS and the predictive models have been aligned and reordered so that all the SNPs have positive GWAS effects. The z-scores are used to compute the weighted average of the model weights (dashed lines), which have the same signs as and are proportional to the predicted effects of protein and gene expression on the GWAS outcome.

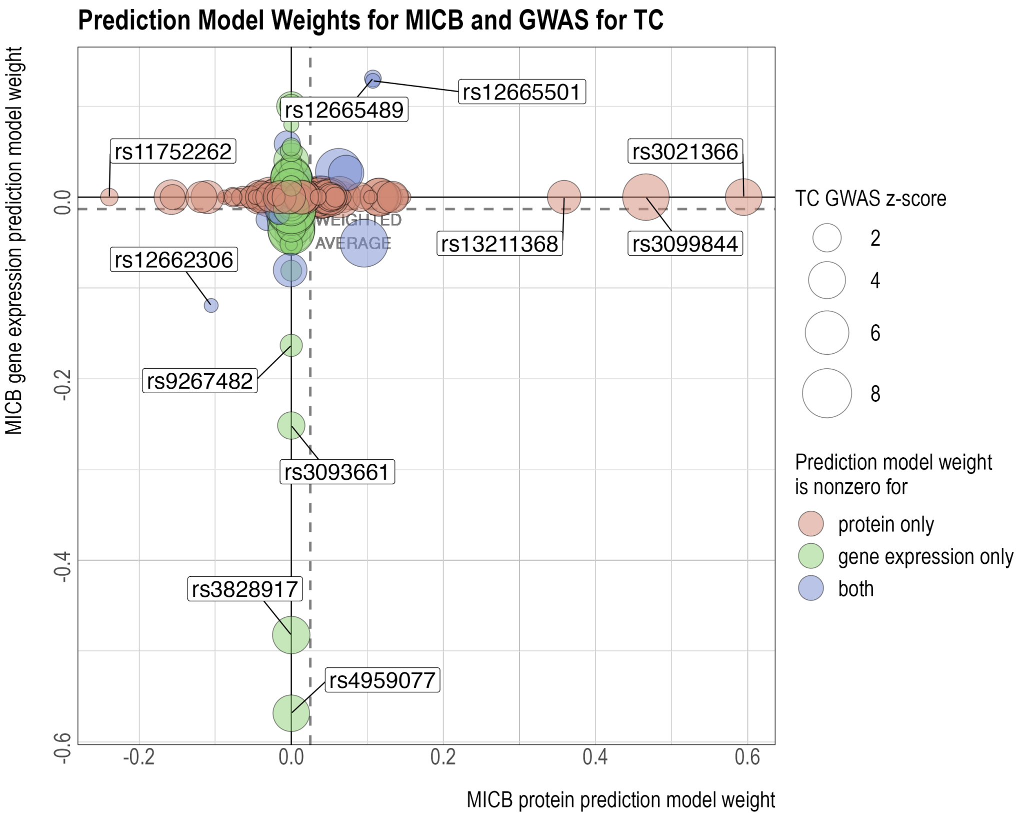

Figure S17: Comparison of MESA whole blood PWAS, MESA PBMC TWAS, and GTEx tissue-specific TWAS results for TC. Panel (a): signed log p-value and significance of association. Missing values are shown in white. Significance of association is deteremined by the false discovery rate (FDR) threshold of 0.05. Panel (b): correlation between signed log p-values of MESA whole blood PWAS and signed log p-values of each GTEx tissue-specific TWAS (i.e. the correlation between the bottom row and every other row of the grid in Panel (a)).

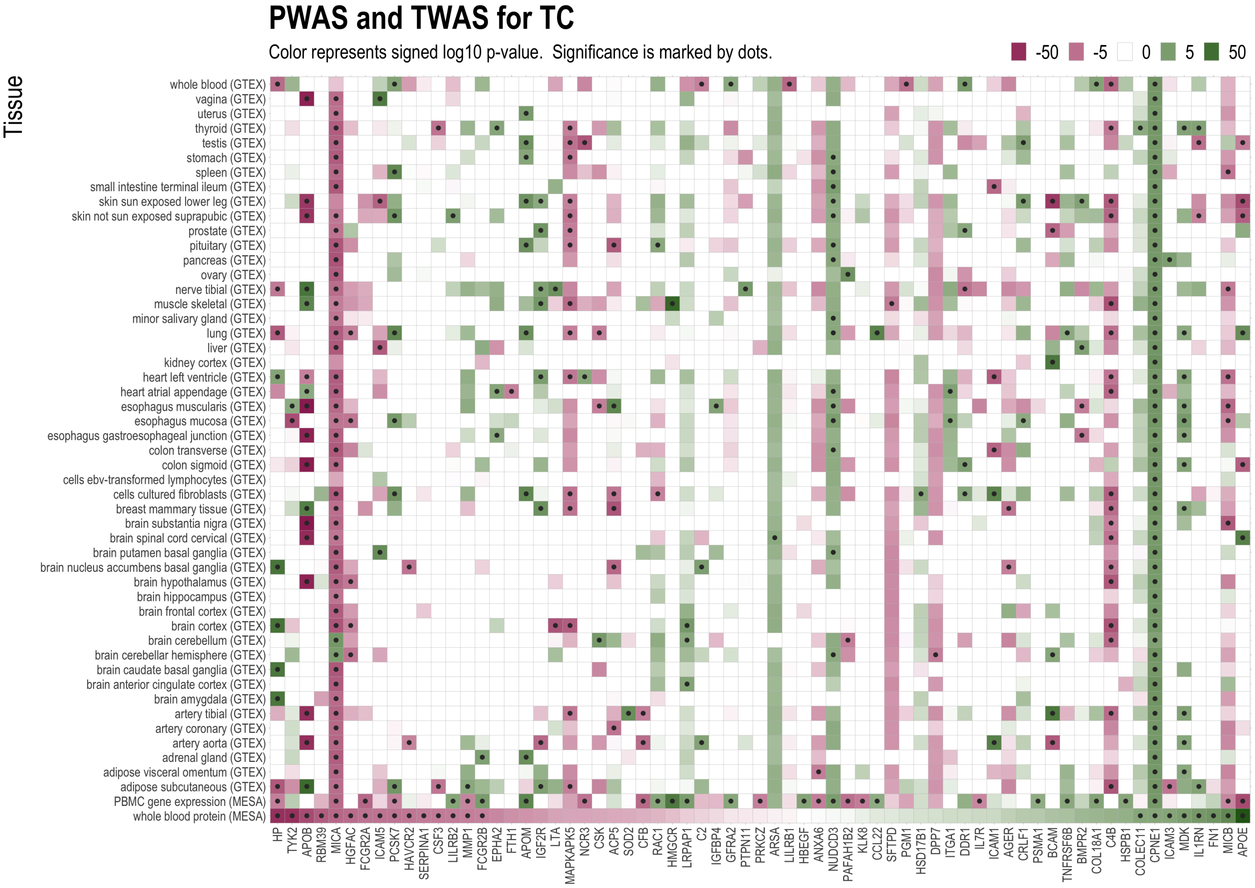

(a)

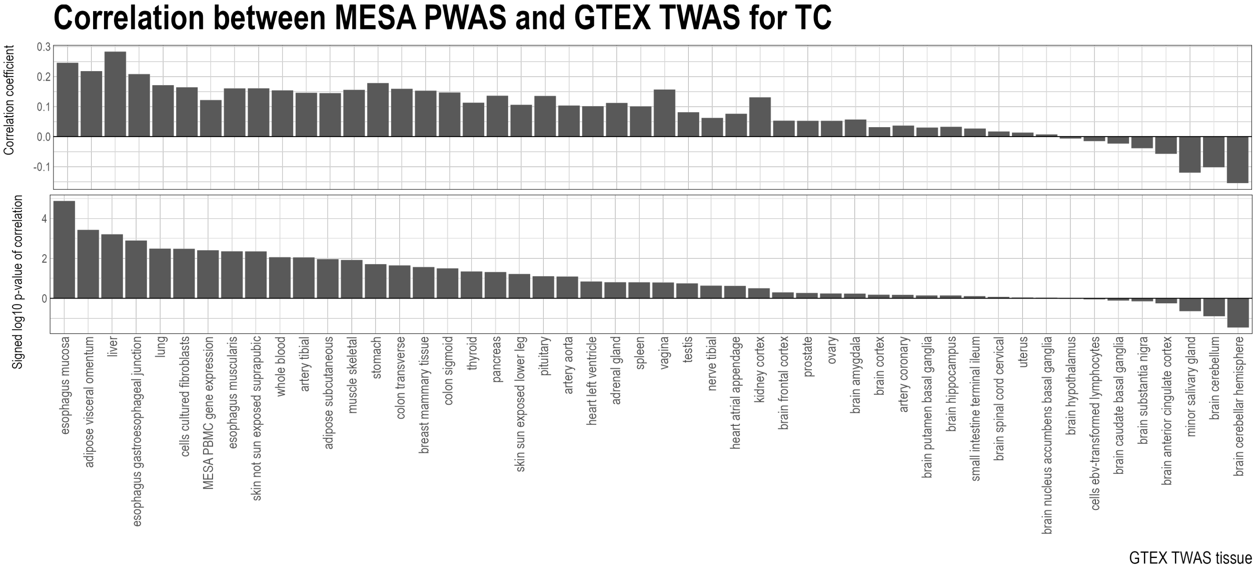

(b)

Figure S18: GWAS for TG and prediction models for APOE's protein and gene expression levels. The reference and alternative alleles for GWAS and the predictive models have been aligned and reordered so that all the SNPs have positive GWAS effects. In the center and bottom panels, the size of the circles indicates the SNP's GWAS z-score. The z-scores are used to compute the weighted average of the model weights (dashed line), which has the same sign as and is proportional to the predicted effect of protein or gene expression on the GWAS outcome.

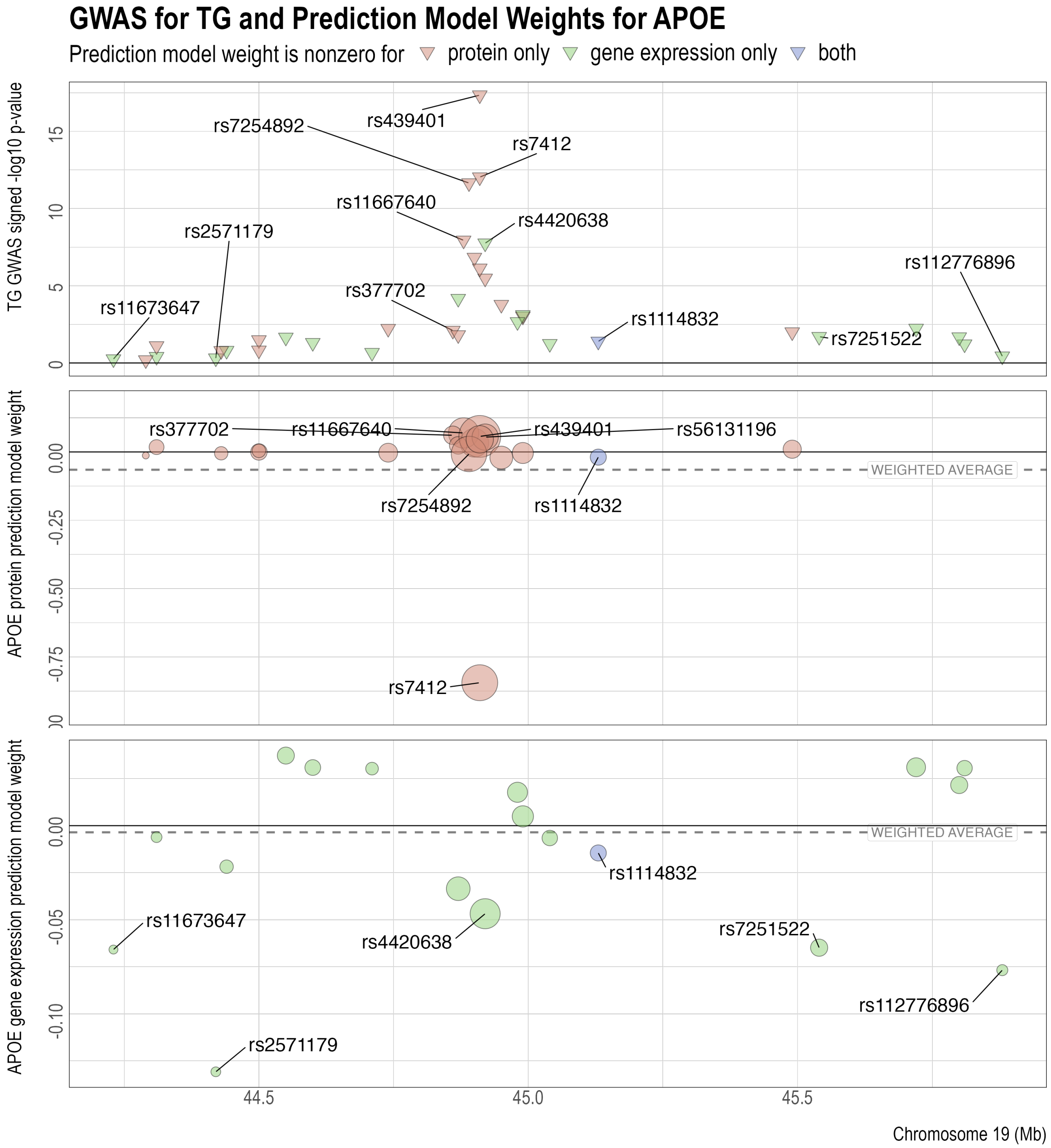

Figure S19: Comparison of APOE's protein and gene expression predictive model weights with the TG GWAS z-scores of the SNPs. The reference and alternative alleles for GWAS and the predictive models have been aligned and reordered so that all the SNPs have positive GWAS effects. The z-scores are used to compute the weighted average of the model weights (dashed lines), which have the same signs as and are proportional to the predicted effects of protein and gene expression on the GWAS outcome.

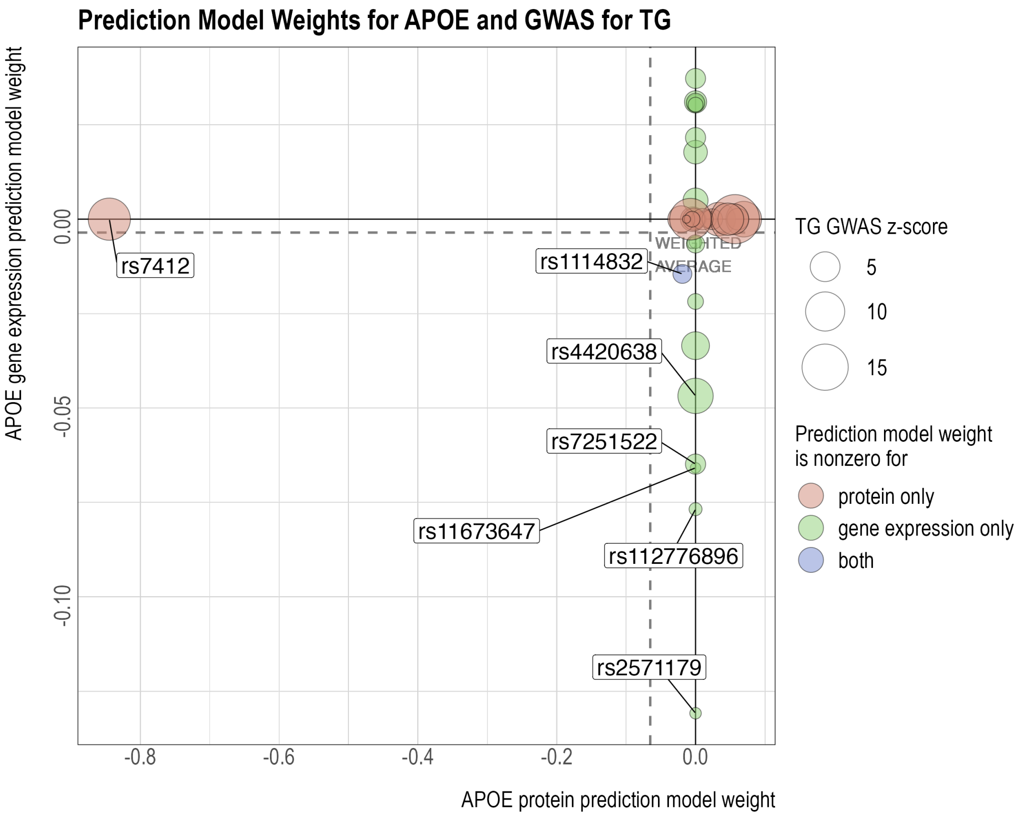

Figure S20: GWAS for TG and prediction models for FCGR2B's protein and gene expression levels. The reference and alternative alleles for GWAS and the predictive models have been aligned and reordered so that all the SNPs have positive GWAS effects. In the center and bottom panels, the size of the circles indicates the SNP's GWAS z-score. The z-scores are used to compute the weighted average of the model weights (dashed line), which has the same sign as and is proportional to the predicted effect of protein or gene expression on the GWAS outcome.

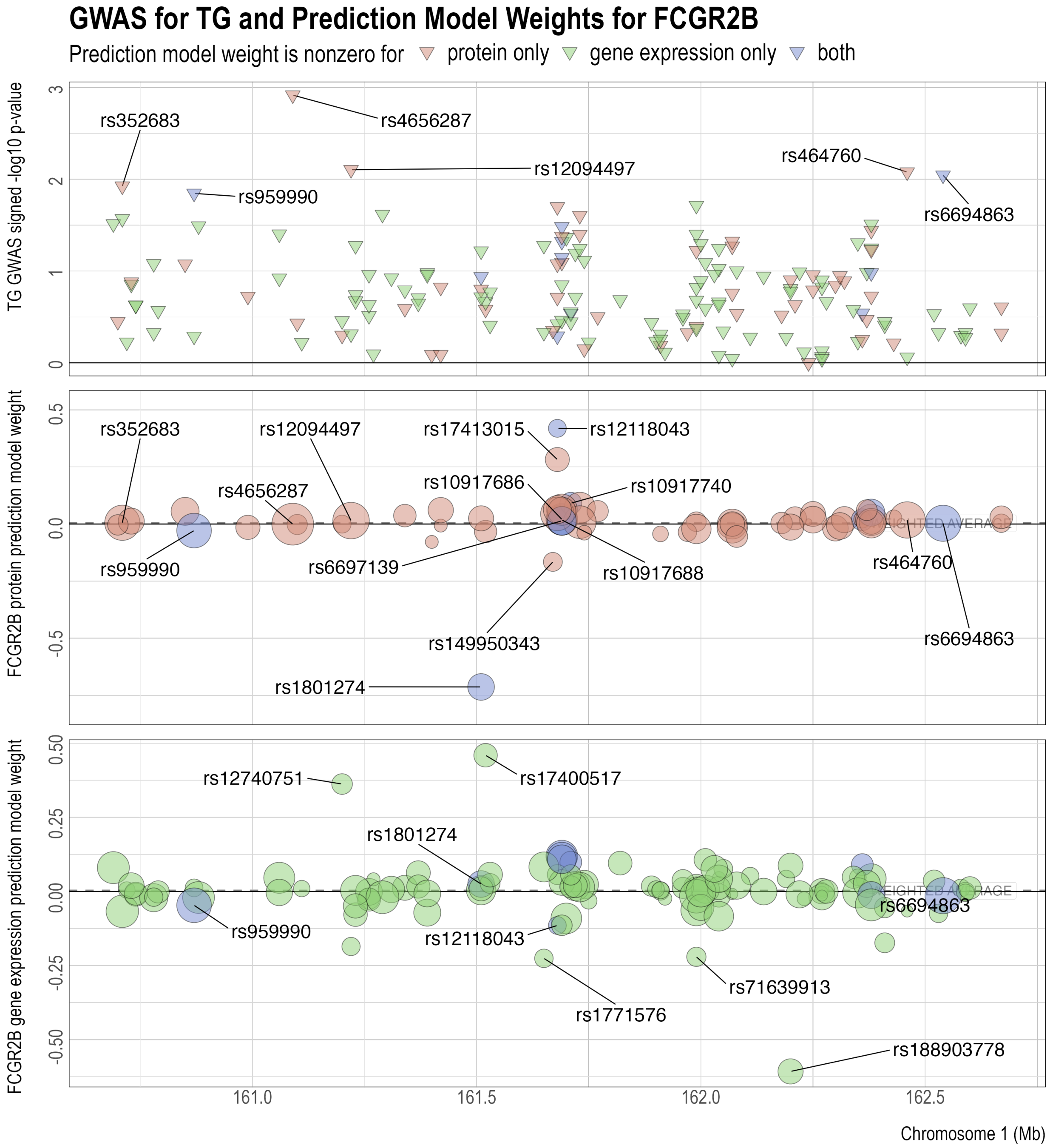

Figure S21: Comparison of FCGR2B's protein and gene expression predictive model weights with the TG GWAS z-scores of the SNPs. The reference and alternative alleles for GWAS and the predictive models have been aligned and reordered so that all the SNPs have positive GWAS effects. The z-scores are used to compute the weighted average of the model weights (dashed lines), which have the same signs as and are proportional to the predicted effects of protein and gene expression on the GWAS outcome.

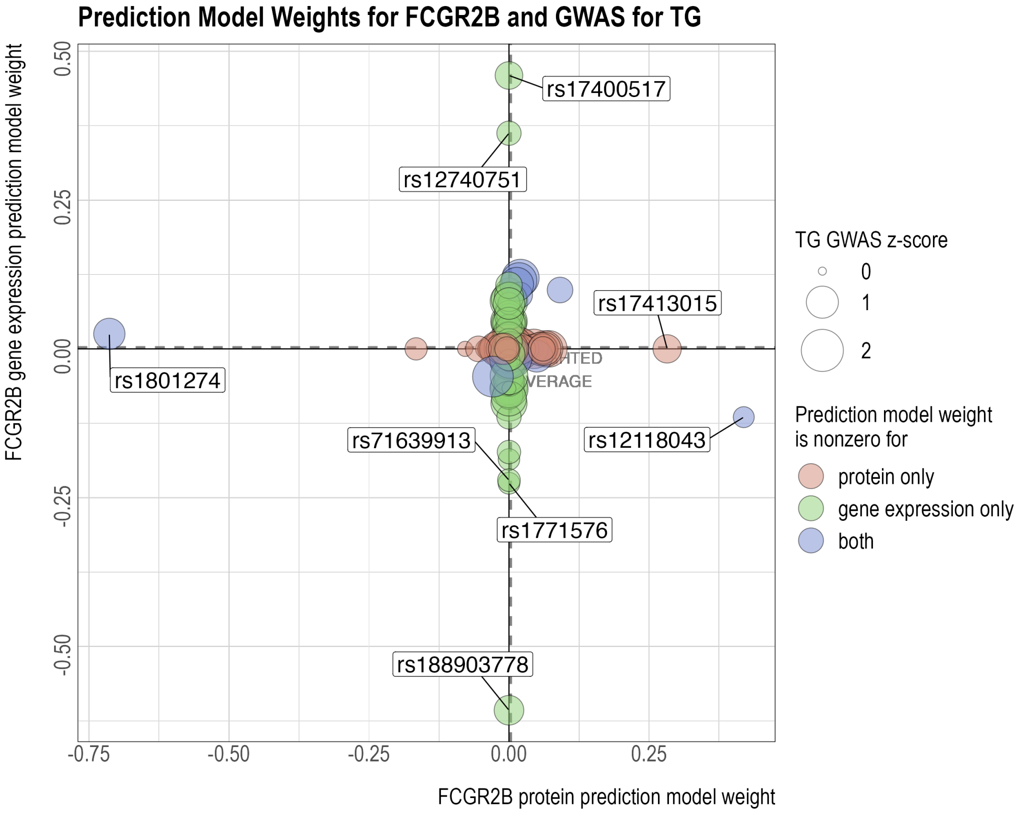

Figure S22: GWAS for TG and prediction models for LILRB2's protein and gene expression levels. The reference and alternative alleles for GWAS and the predictive models have been aligned and reordered so that all the SNPs have positive GWAS effects. In the center and bottom panels, the size of the circles indicates the SNP's GWAS z-score. The z-scores are used to compute the weighted average of the model weights (dashed line), which has the same sign as and is proportional to the predicted effect of protein or gene expression on the GWAS outcome.

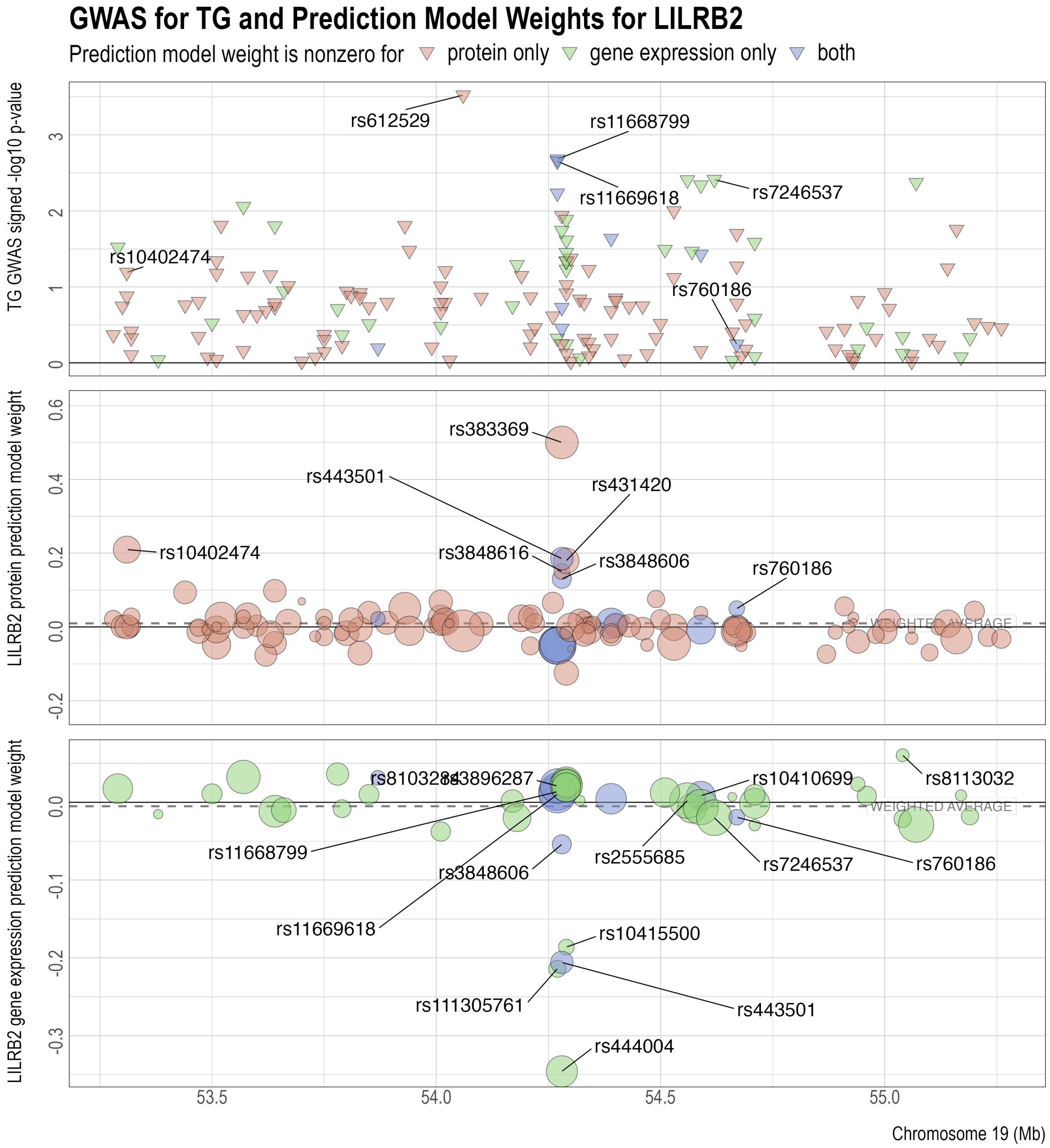

Figure S23: Comparison of LILRB2's protein and gene expression predictive model weights with the TG GWAS z-scores of the SNPs. The reference and alternative alleles for GWAS and the predictive models have been aligned and reordered so that all the SNPs have positive GWAS effects. The z-scores are used to compute the weighted average of the model weights (dashed lines), which have the same signs as and are proportional to the predicted effects of protein and gene expression on the GWAS outcome.

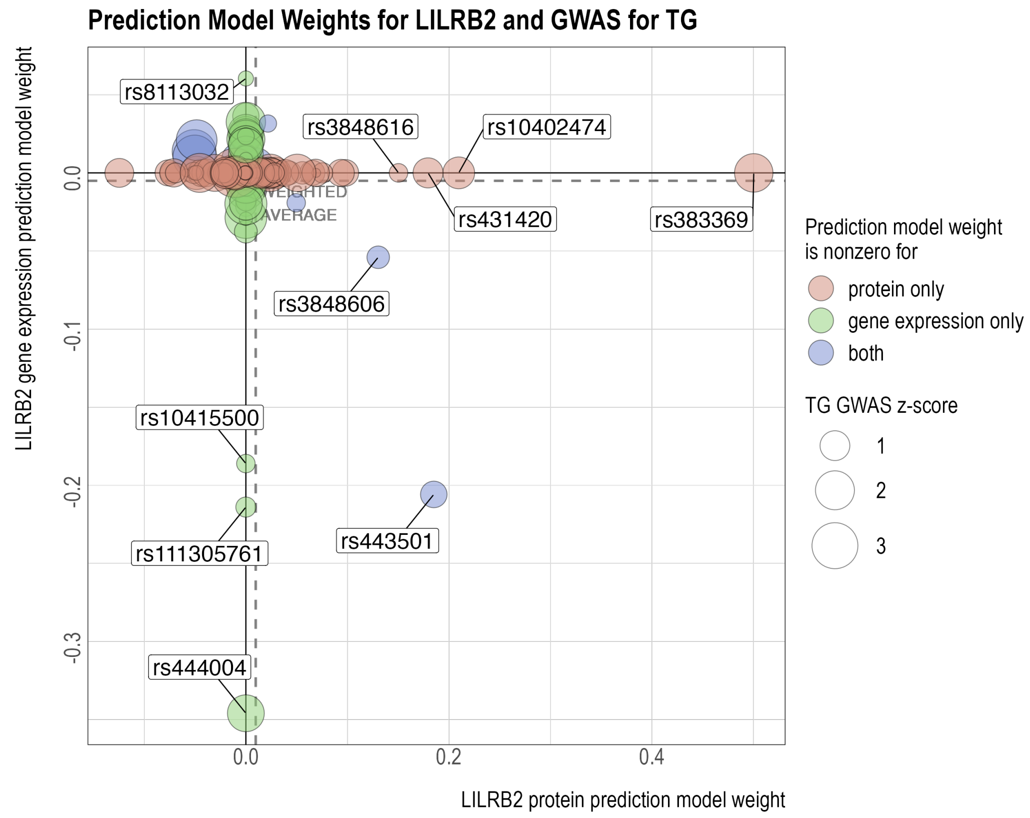

Figure S24: GWAS for TG and prediction models for MICB's protein and gene expression levels. The reference and alternative alleles for GWAS and the predictive models have been aligned and reordered so that all the SNPs have positive GWAS effects. In the center and bottom panels, the size of the circles indicates the SNP's GWAS z-score. The z-scores are used to compute the weighted average of the model weights (dashed line), which has the same sign as and is proportional to the predicted effect of protein or gene expression on the GWAS outcome.

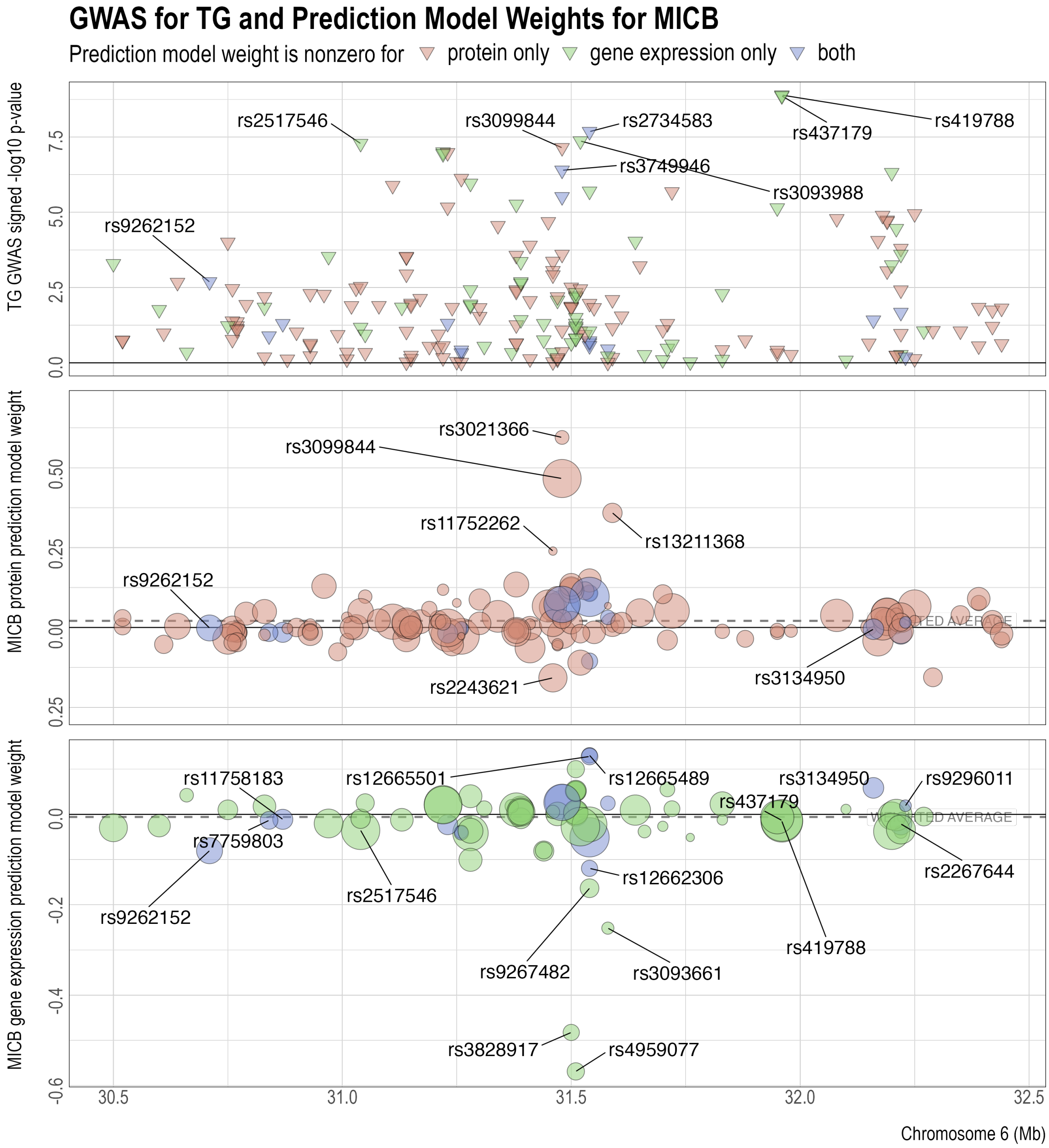

Figure S25: Comparison of MICB's protein and gene expression predictive model weights with the TG GWAS z-scores of the SNPs. The reference and alternative alleles for GWAS and the predictive models have been aligned and reordered so that all the SNPs have positive GWAS effects. The z-scores are used to compute the weighted average of the model weights (dashed lines), which have the same signs as and are proportional to the predicted effects of protein and gene expression on the GWAS outcome.

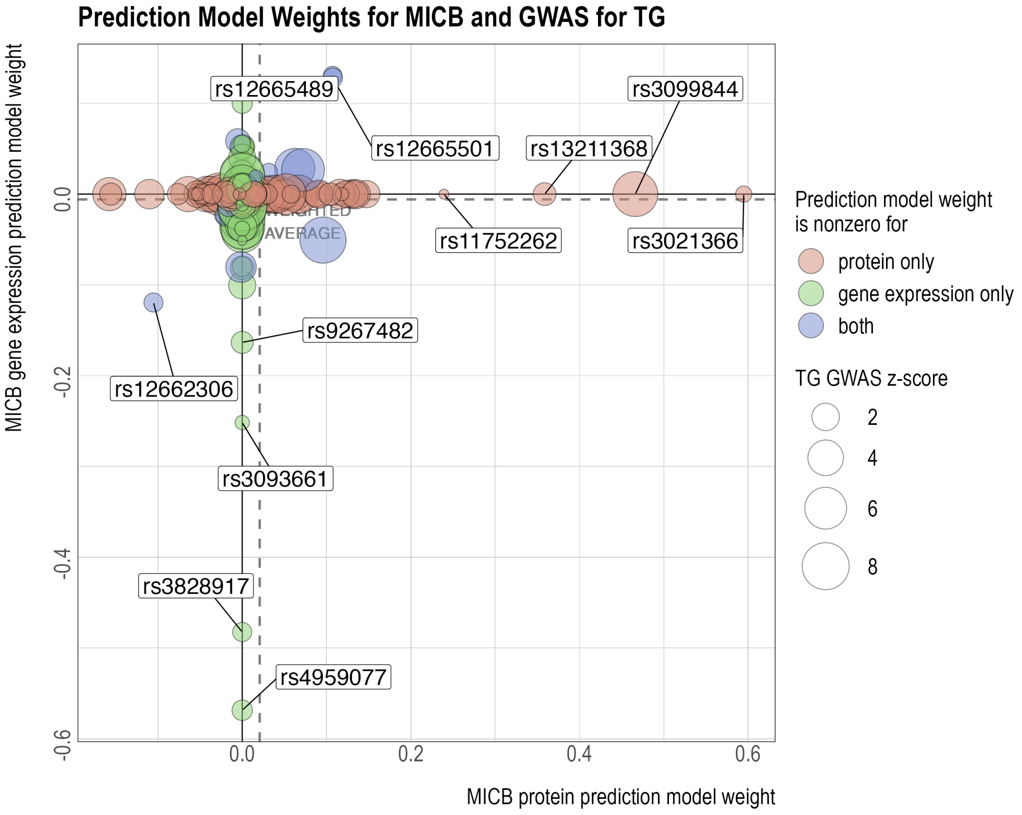

Figure S26: Comparison of MESA whole blood PWAS, MESA PBMC TWAS, and GTEx tissue-specific TWAS results for TG. Panel (a): signed log p-value and significance of association. Missing values are shown in white. Significance of association is deteremined by the false discovery rate (FDR) threshold of 0.05. Panel (b): correlation between signed log p-values of MESA whole blood PWAS and signed log p-values of each GTEx tissue-specific TWAS (i.e. the correlation between the bottom row and every other row of the grid in Panel (a)).

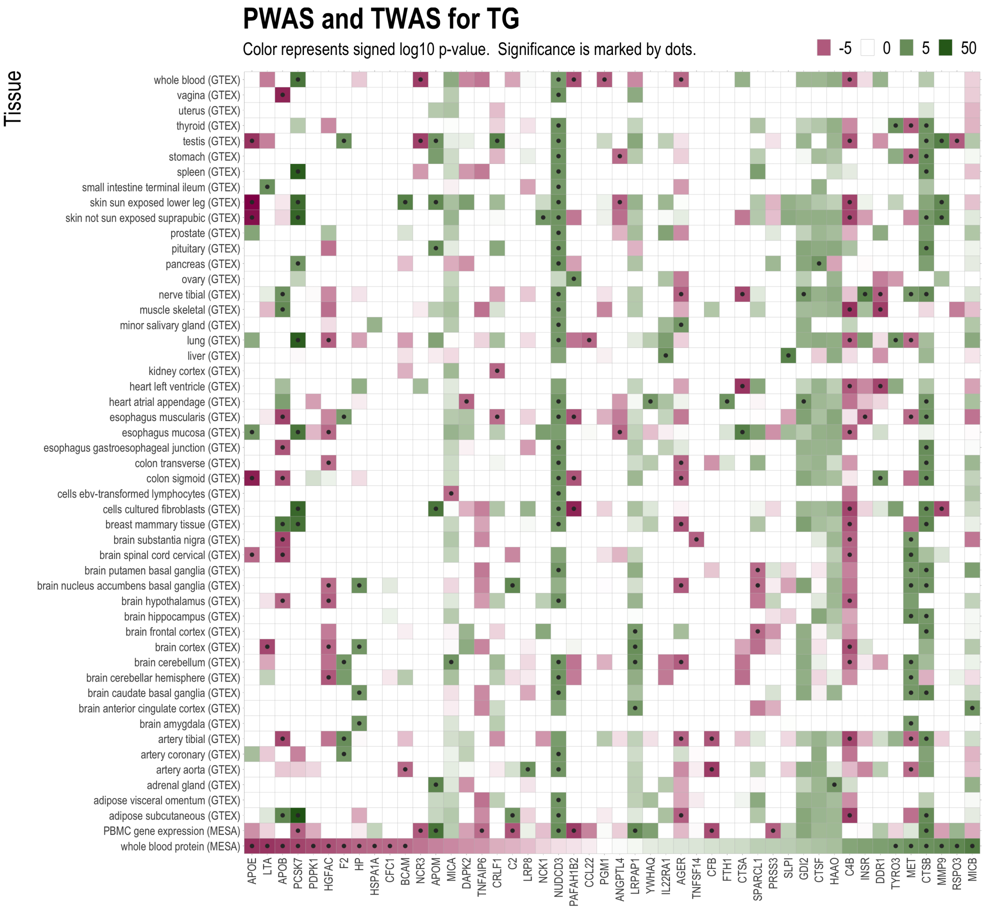

(a)

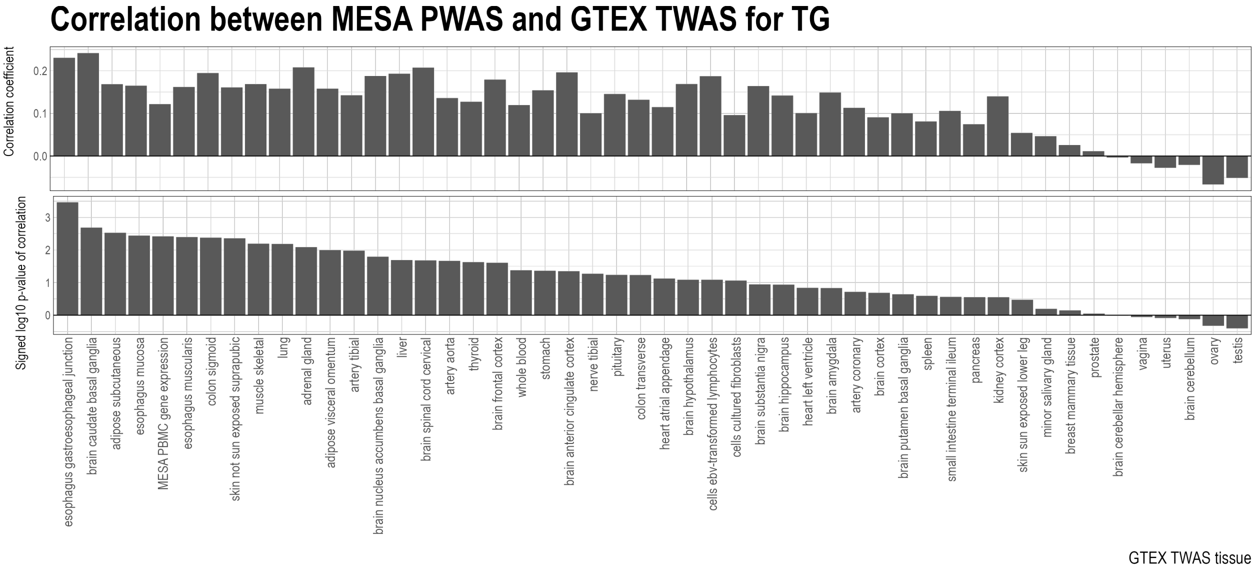

(b)

Figure S27: GWAS for HDL and prediction models for APOE's protein and gene expression levels. The reference and alternative alleles for GWAS and the predictive models have been aligned and reordered so that all the SNPs have positive GWAS effects. In the center and bottom panels, the size of the circles indicates the SNP's GWAS z-score. The z-scores are used to compute the weighted average of the model weights (dashed line), which has the same sign as and is proportional to the predicted effect of protein or gene expression on the GWAS outcome.

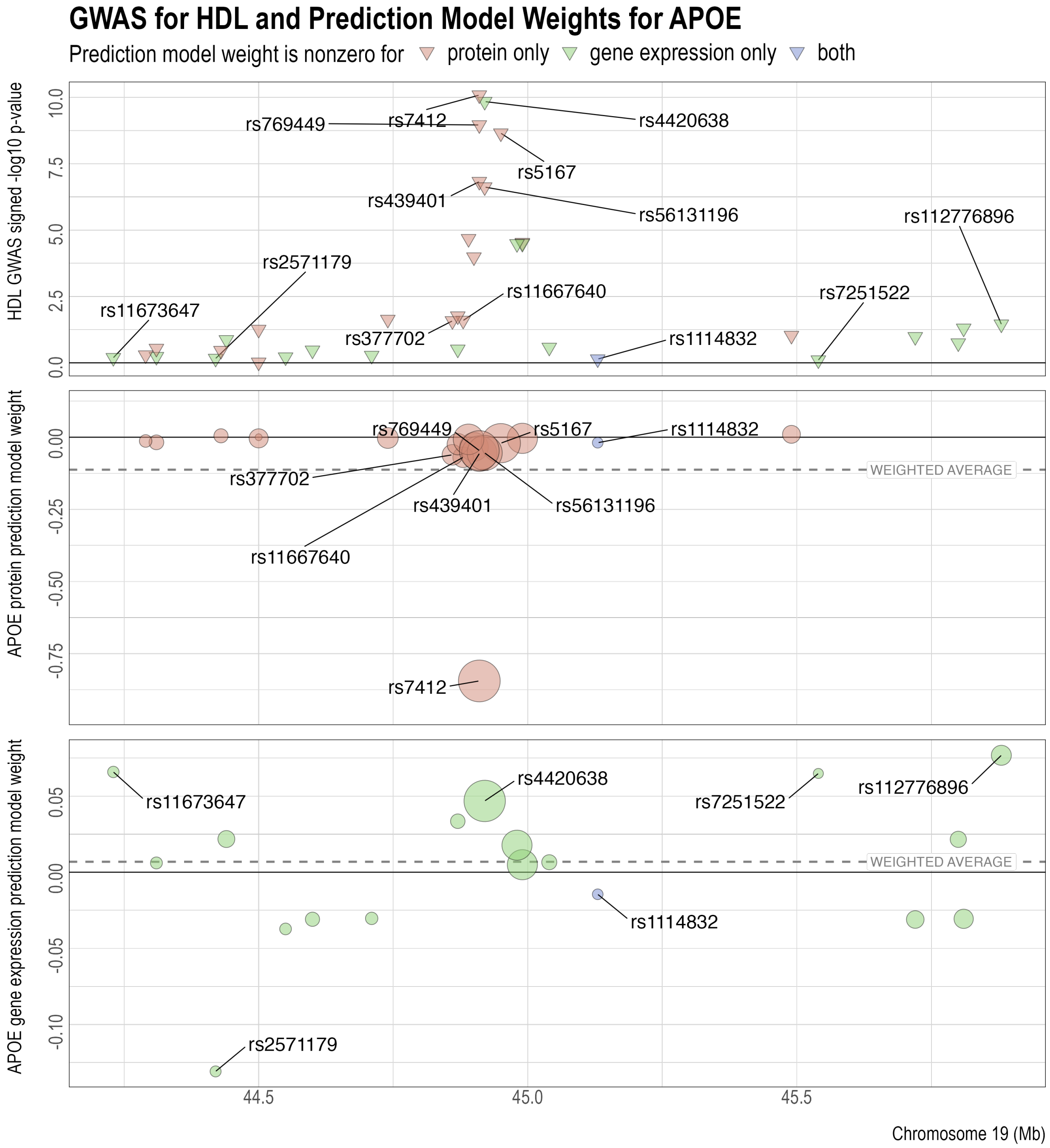

Figure S28: Comparison of APOE's protein and gene expression predictive model weights with the HDL GWAS z-scores of the SNPs. The reference and alternative alleles for GWAS and the predictive models have been aligned and reordered so that all the SNPs have positive GWAS effects. The z-scores are used to compute the weighted average of the model weights (dashed lines), which have the same signs as and are proportional to the predicted effects of protein and gene expression on the GWAS outcome.

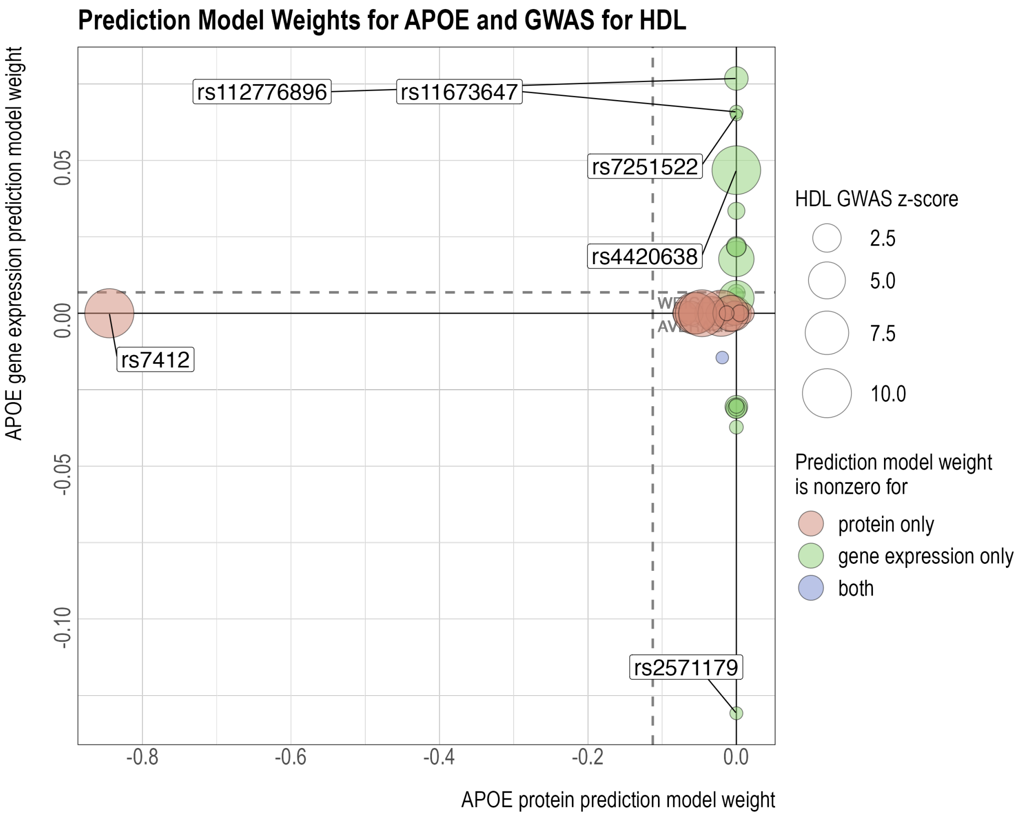

Figure S29: GWAS for HDL and prediction models for FCGR2B's protein and gene expression levels. The reference and alternative alleles for GWAS and the predictive models have been aligned and reordered so that all the SNPs have positive GWAS effects. In the center and bottom panels, the size of the circles indicates the SNP's GWAS z-score. The z-scores are used to compute the weighted average of the model weights (dashed line), which has the same sign as and is proportional to the predicted effect of protein or gene expression on the GWAS outcome.

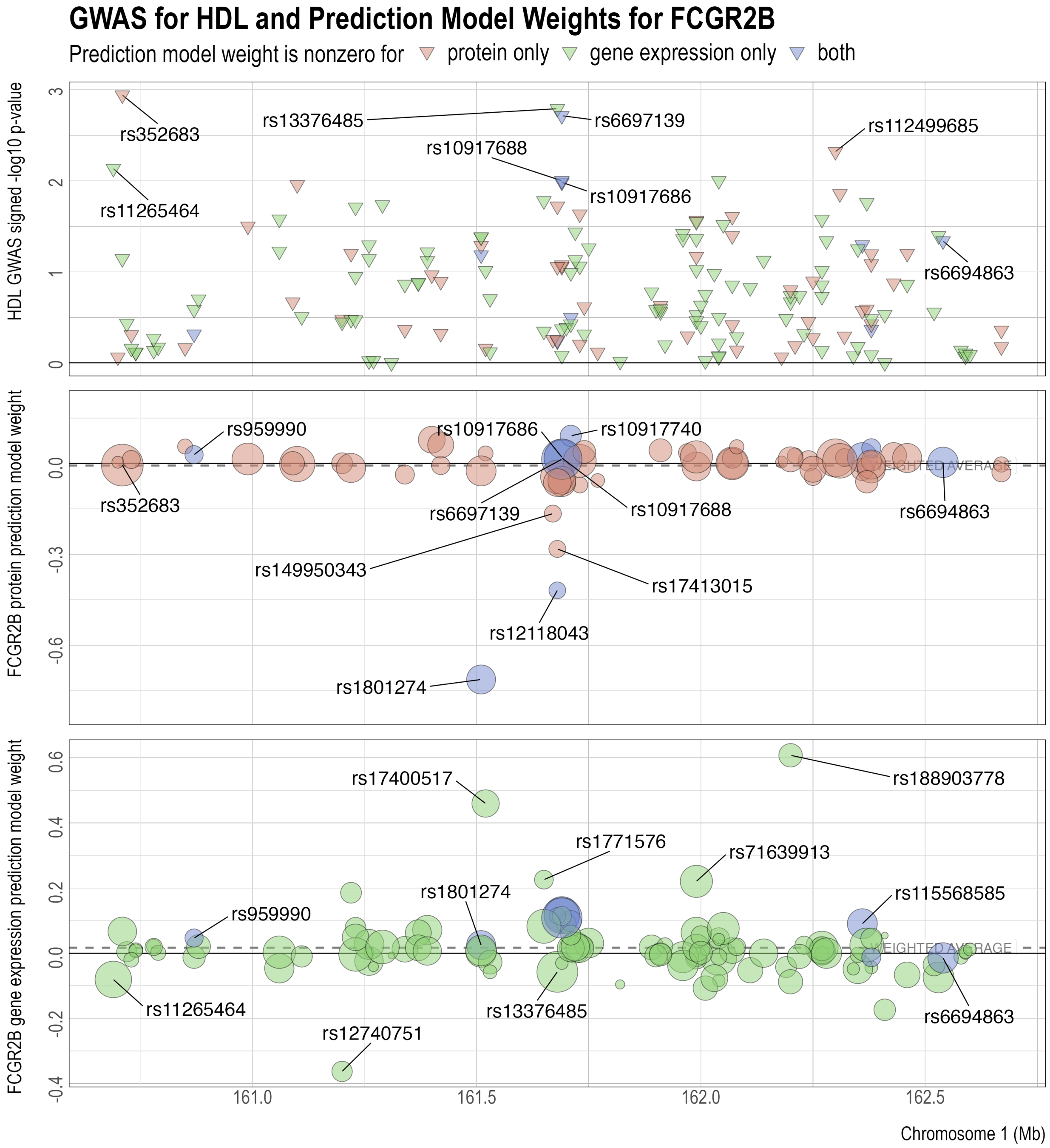

Figure S30: Comparison of FCGR2B's protein and gene expression predictive model weights with the HDL GWAS z-scores of the SNPs. The reference and alternative alleles for GWAS and the predictive models have been aligned and reordered so that all the SNPs have positive GWAS effects. The z-scores are used to compute the weighted average of the model weights (dashed lines), which have the same signs as and are proportional to the predicted effects of protein and gene expression on the GWAS outcome.

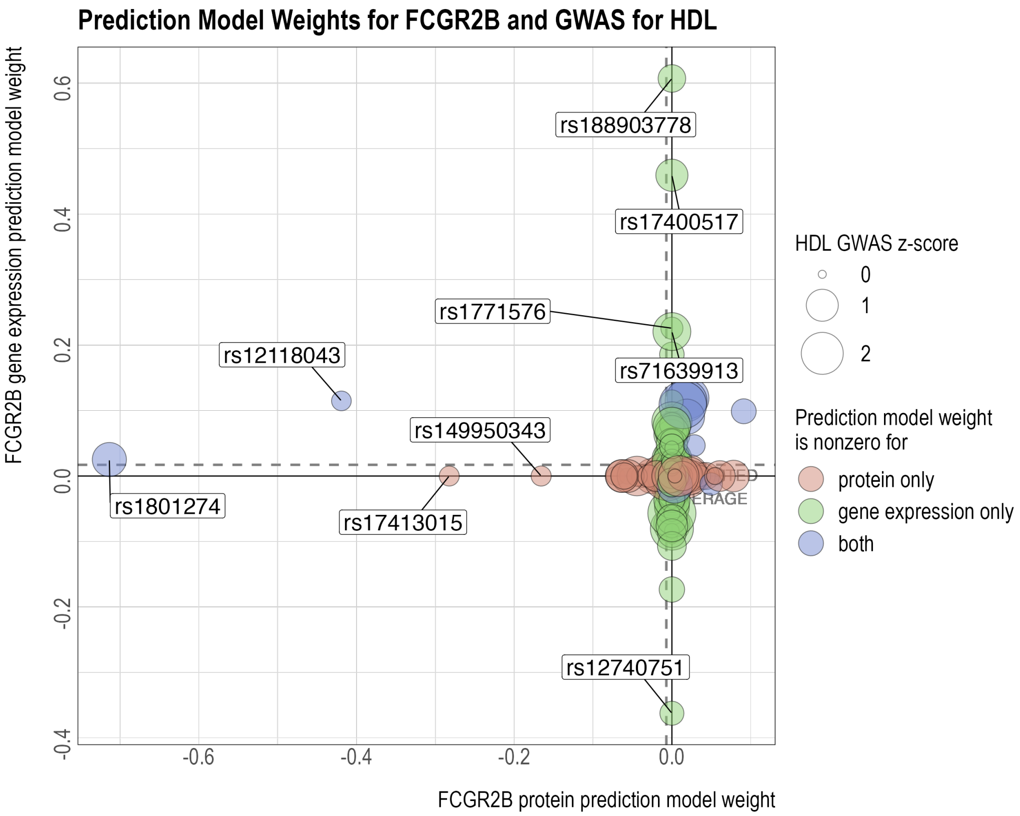

Figure S31: GWAS for HDL and prediction models for LILRB2's protein and gene expression levels. The reference and alternative alleles for GWAS and the predictive models have been aligned and reordered so that all the SNPs have positive GWAS effects. In the center and bottom panels, the size of the circles indicates the SNP's GWAS z-score. The z-scores are used to compute the weighted average of the model weights (dashed line), which has the same sign as and is proportional to the predicted effect of protein or gene expression on the GWAS outcome.

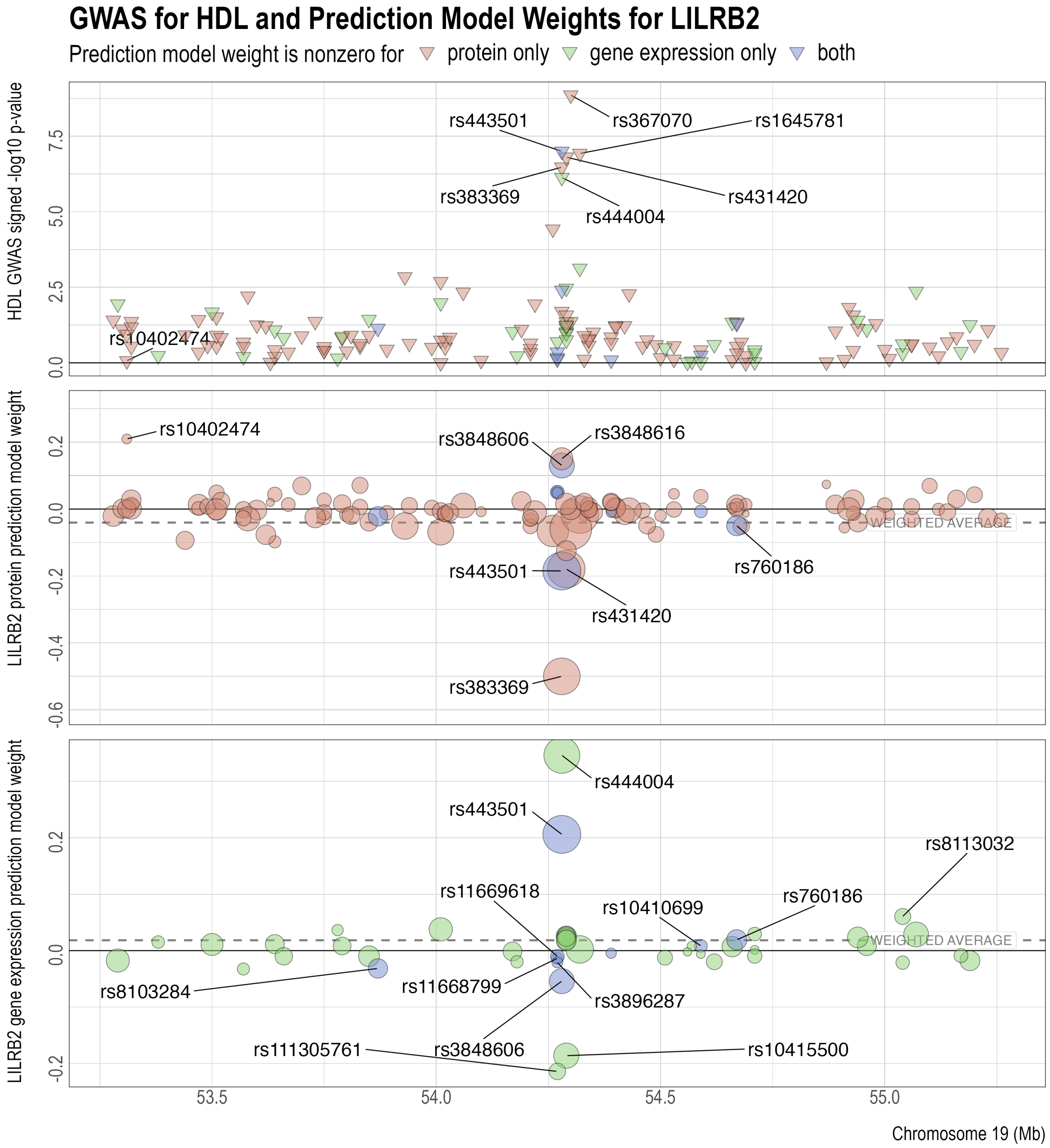

Figure S32: Comparison of LILRB2's protein and gene expression predictive model weights with the HDL GWAS z-scores of the SNPs. The reference and alternative alleles for GWAS and the predictive models have been aligned and reordered so that all the SNPs have positive GWAS effects. The z-scores are used to compute the weighted average of the model weights (dashed lines), which have the same signs as and are proportional to the predicted effects of protein and gene expression on the GWAS outcome.

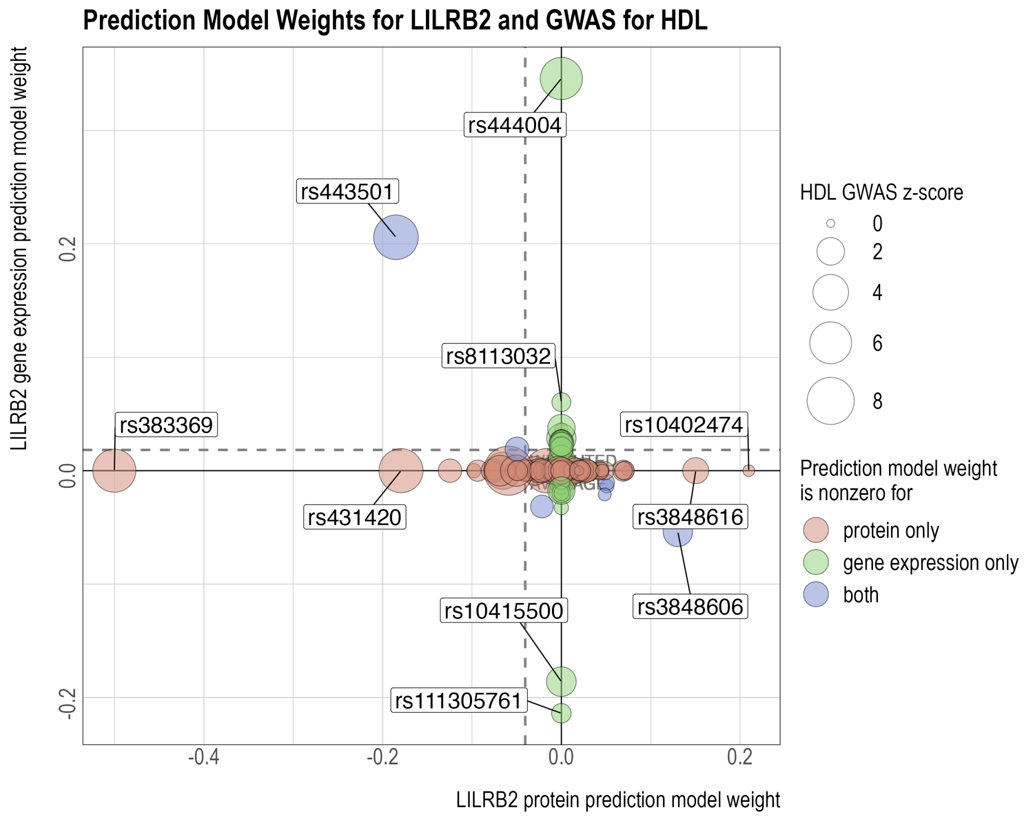

Figure S33: GWAS for HDL and prediction models for MICB's protein and gene expression levels. The reference and alternative alleles for GWAS and the predictive models have been aligned and reordered so that all the SNPs have positive GWAS effects. In the center and bottom panels, the size of the circles indicates the SNP's GWAS z-score. The z-scores are used to compute the weighted average of the model weights (dashed line), which has the same sign as and is proportional to the predicted effect of protein or gene expression on the GWAS outcome.

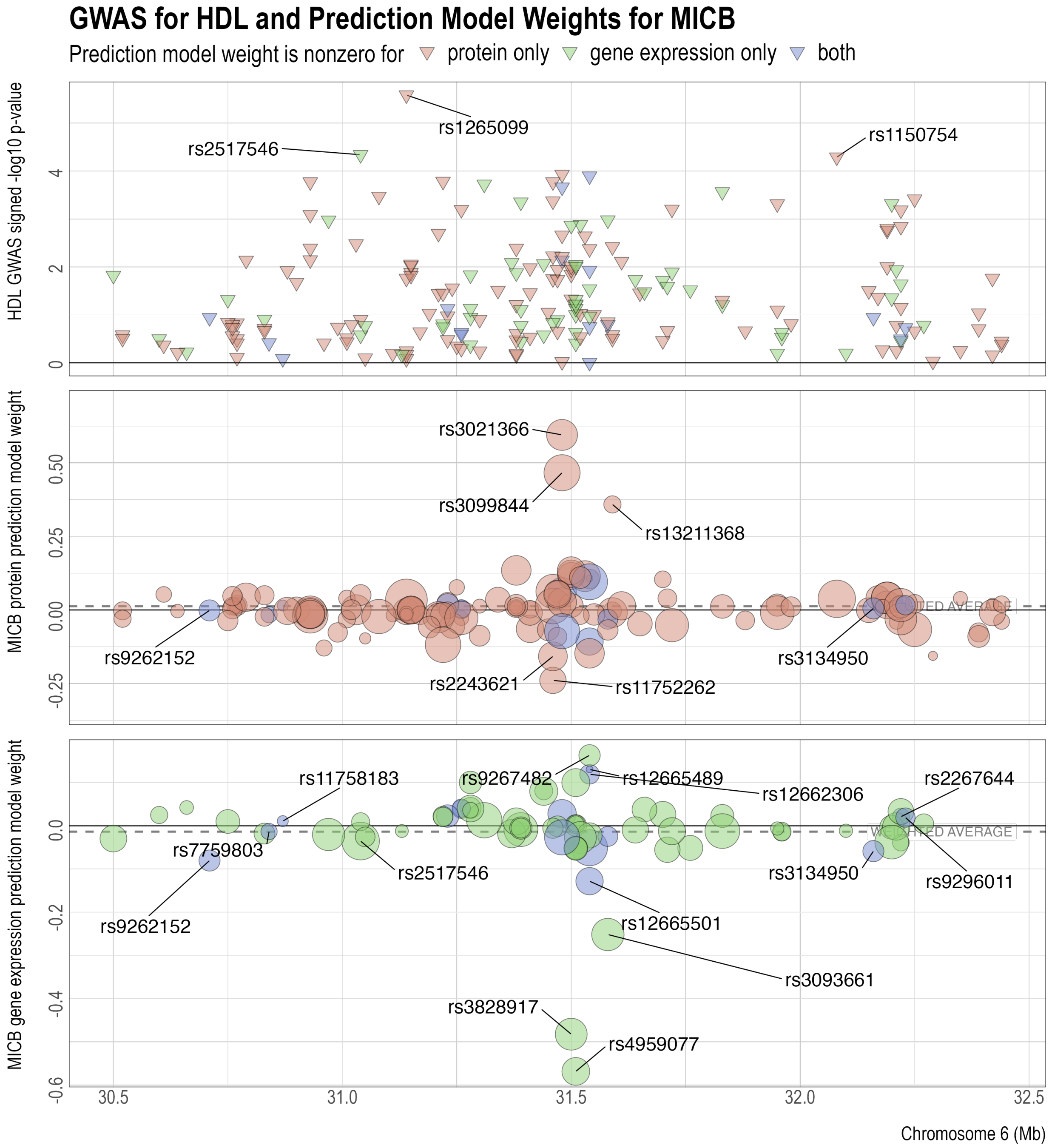

Figure S34: Comparison of MICB's protein and gene expression predictive model weights with the HDL GWAS z-scores of the SNPs. The reference and alternative alleles for GWAS and the predictive models have been aligned and reordered so that all the SNPs have positive GWAS effects. The z-scores are used to compute the weighted average of the model weights (dashed lines), which have the same signs as and are proportional to the predicted effects of protein and gene expression on the GWAS outcome.

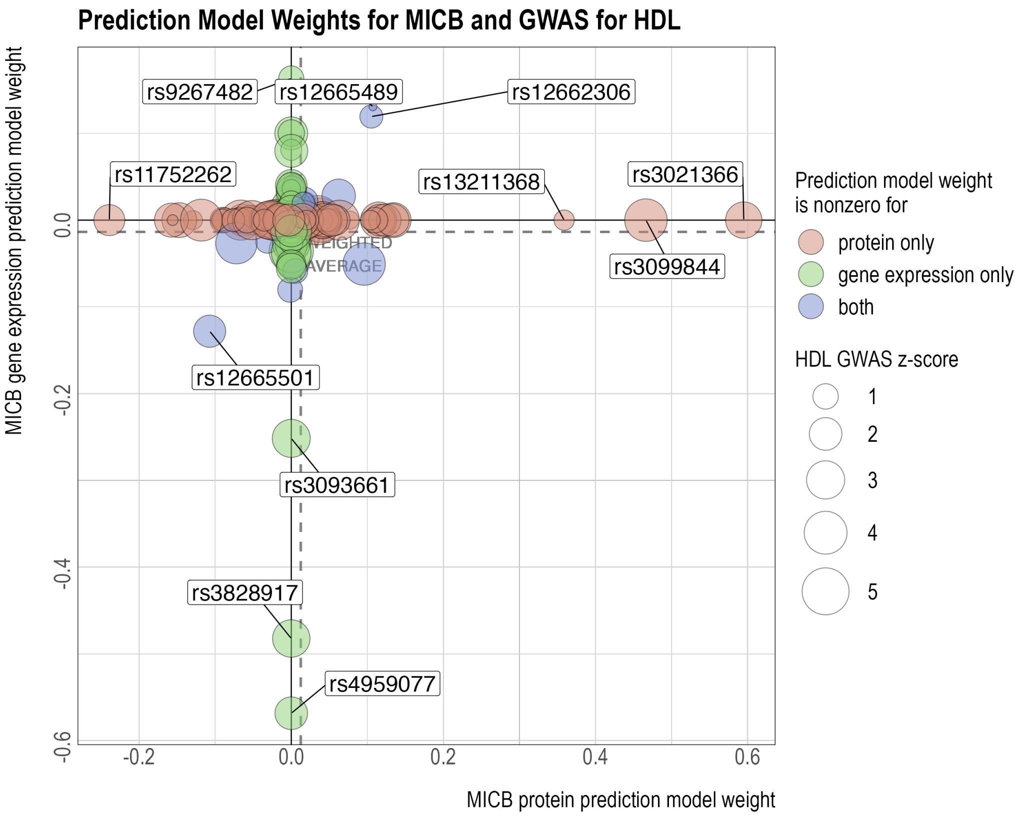

Figure S35: Comparison of MESA whole blood PWAS, MESA PBMC TWAS, and GTEx tissue-specific TWAS results for HDL. Panel (a): signed log p-value and significance of association. Missing values are shown in white. Significance of association is deteremined by the false discovery rate (FDR) threshold of 0.05. Panel (b): correlation between signed log p-values of MESA whole blood PWAS and signed log p-values of each GTEx tissue-specific TWAS (i.e. the correlation between the bottom row and every other row of the grid in Panel (a)).

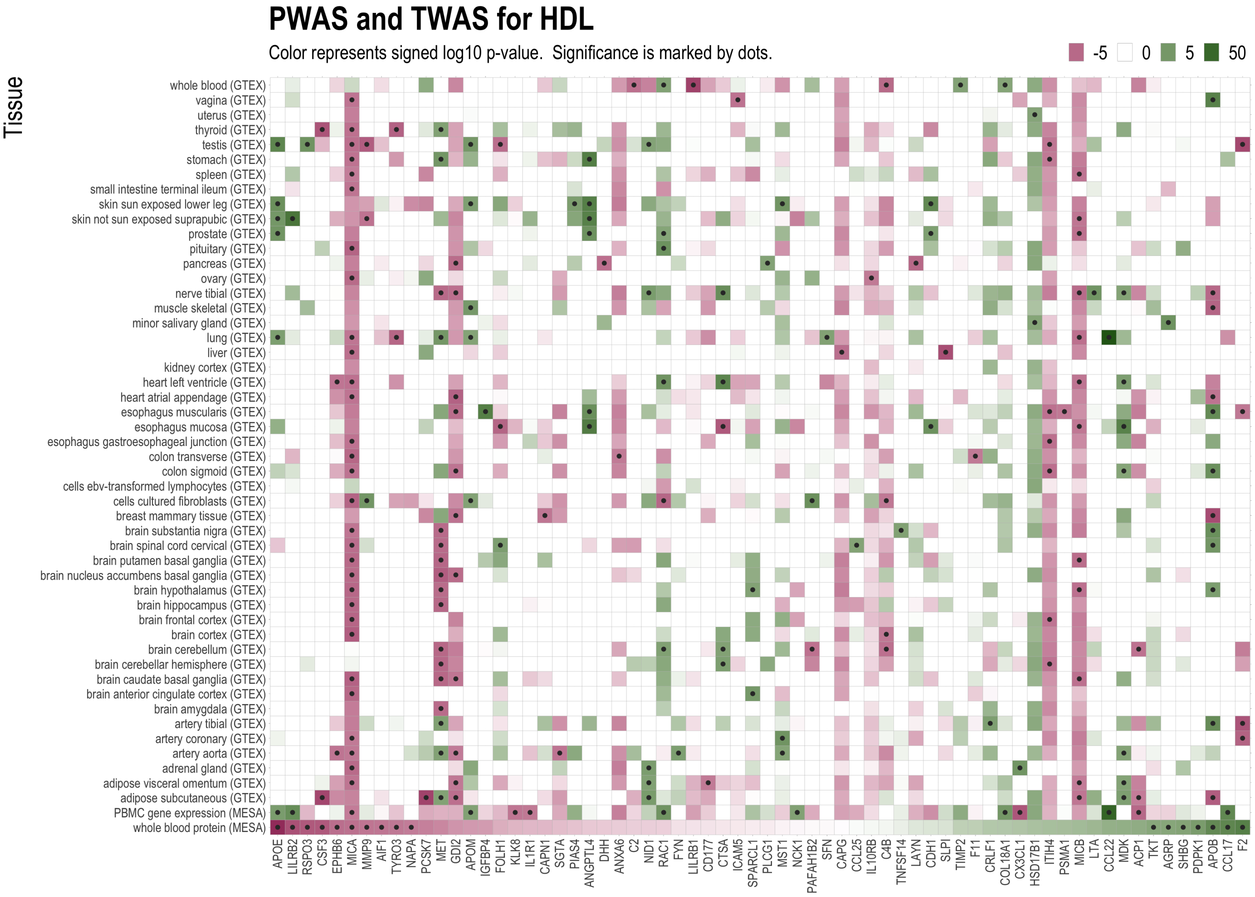

(a)

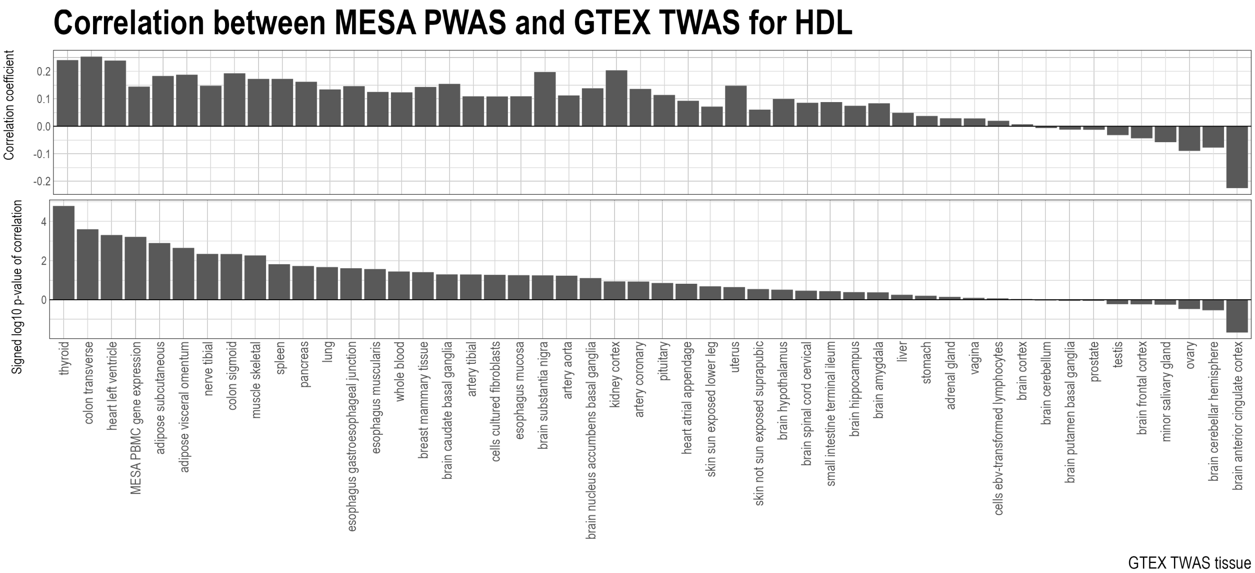

(b)

Figure S36: Correlation between the abundance levels of proteins and gene expressions in the MESA data. The histogram shows the distribution of this correlation for all the genes.

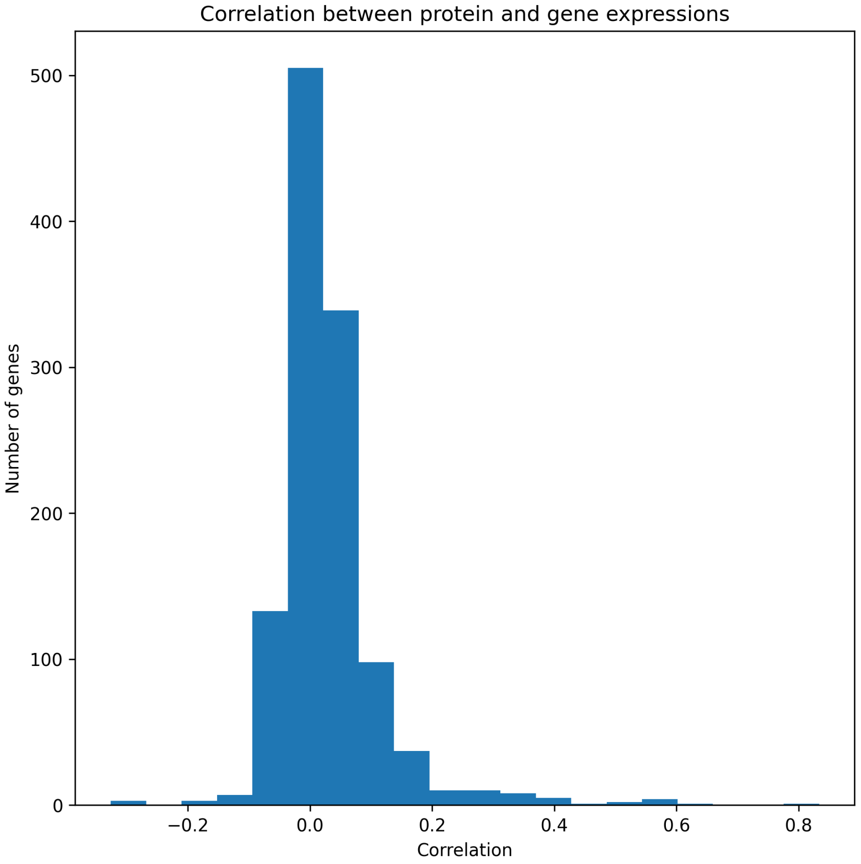

Figure S37: Comparison of the protein-gene expression correlation for the abundance levels directly measured in MESA and those predicted by the models trained using the MESA data. Each dot represents a gene.

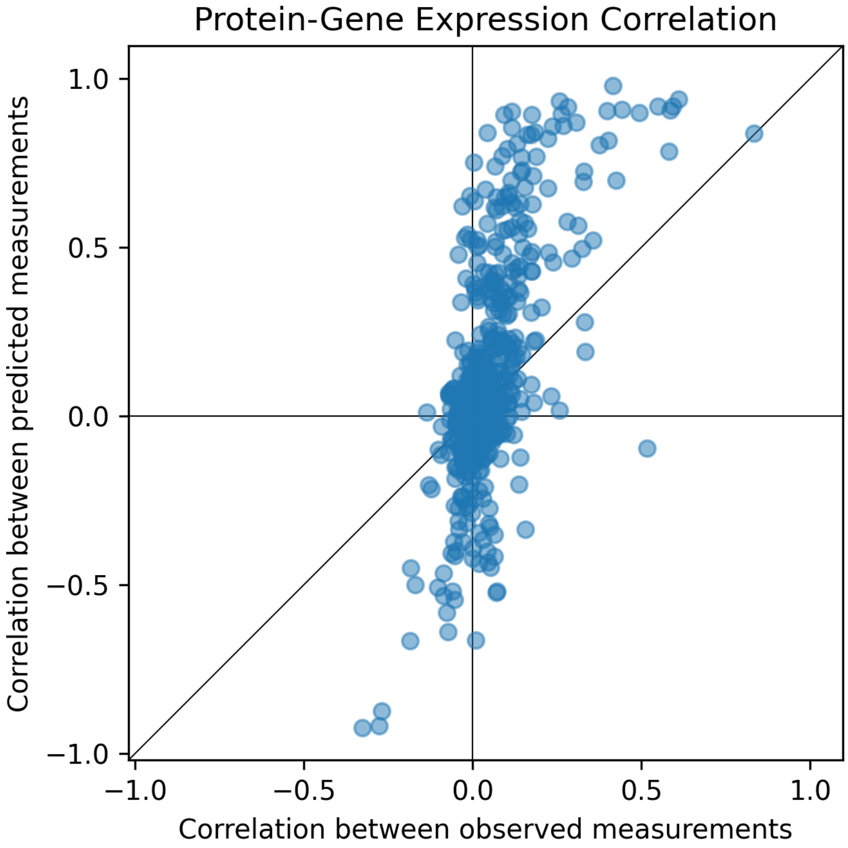

Figure S38: Linkage Disequilibrium of APOE cis SNPs. Only SNPs with large weights in the protein or gene expression predictive model (marked by a P or G before the SNP name, respectively) are displayed.

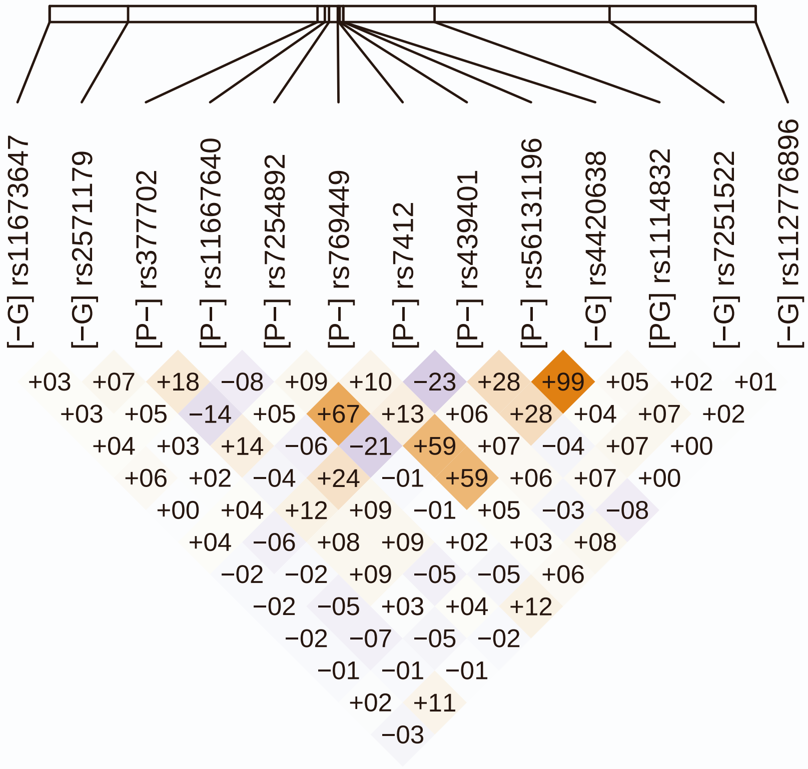

Figure S39: Linkage Disequilibrium of FCGR2B cis SNPs. Only SNPs with large weights in the protein or gene expression predictive model (marked by a P or G before the SNP name, respectively) are displayed.

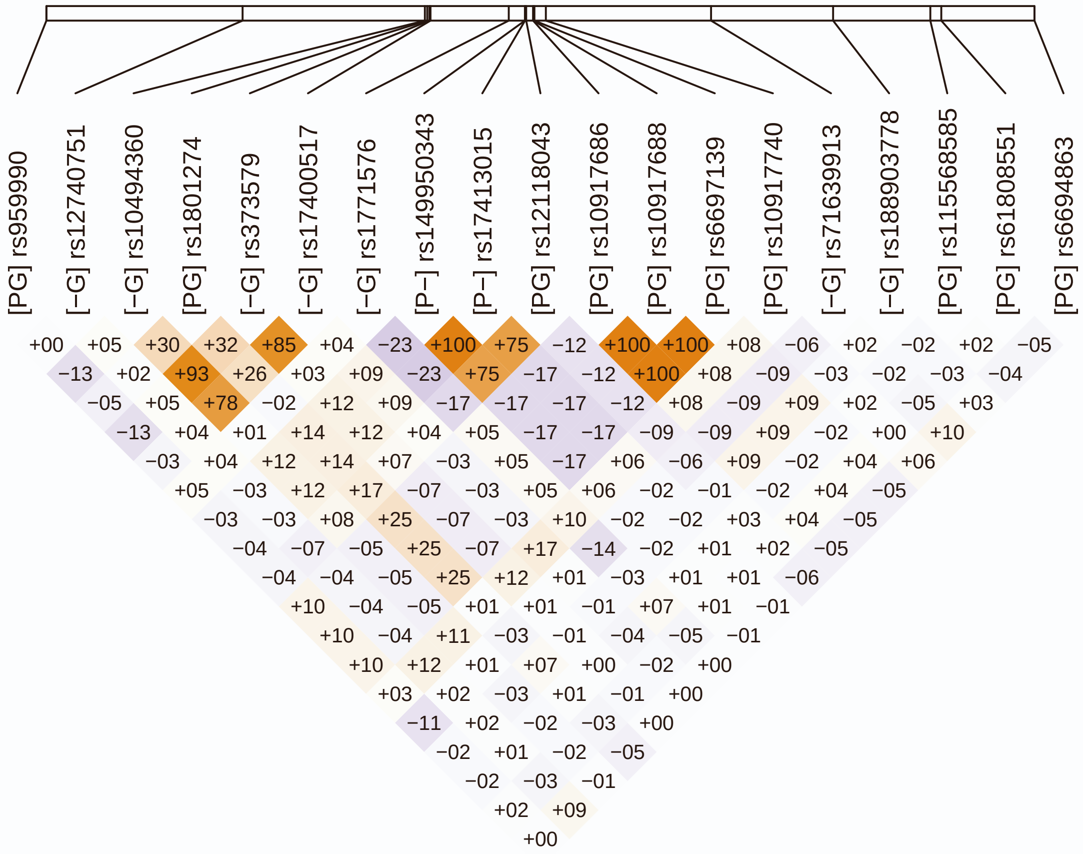

Figure S40: Linkage Disequilibrium of LILRB2 cis SNPs. Only SNPs with large weights in the protein or gene expression predictive model (marked by a P or G before the SNP name, respectively) are displayed.

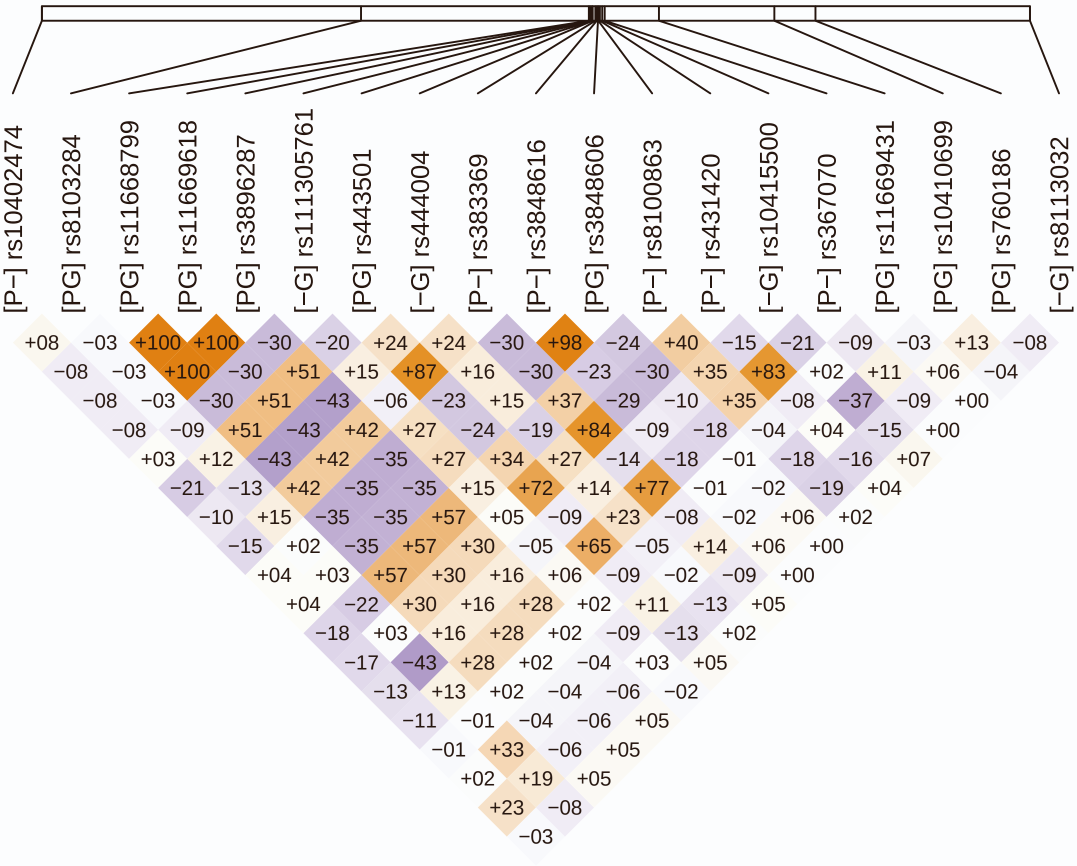

Figure S41: Linkage Disequilibrium of MICB cis SNPs. Only SNPs with large weights in the protein or gene expression predictive model (marked by a P or G before the SNP name, respectively) are displayed.

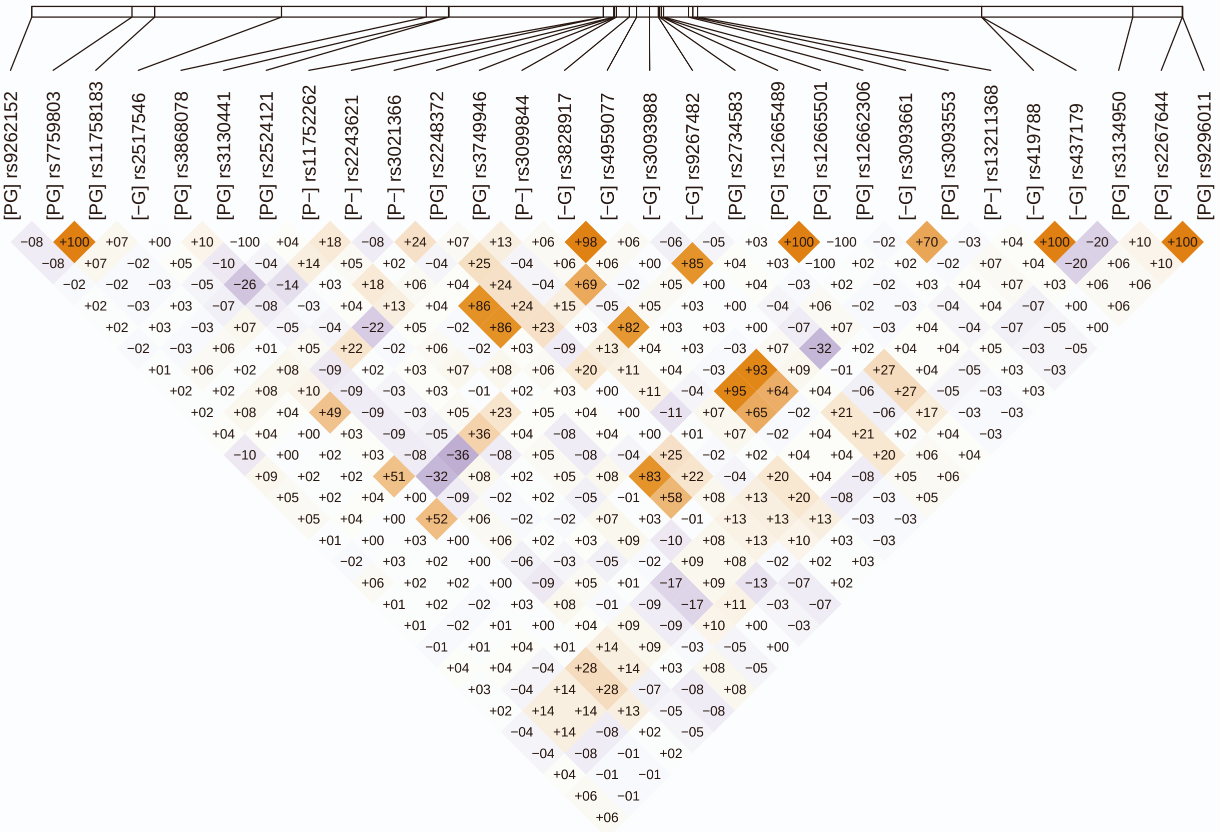

Figure S42: Correlation between signed log p-values of MESA PBMC TWAS for TC and signed log p-values of GTEx tissue-specific TWAS for TC.

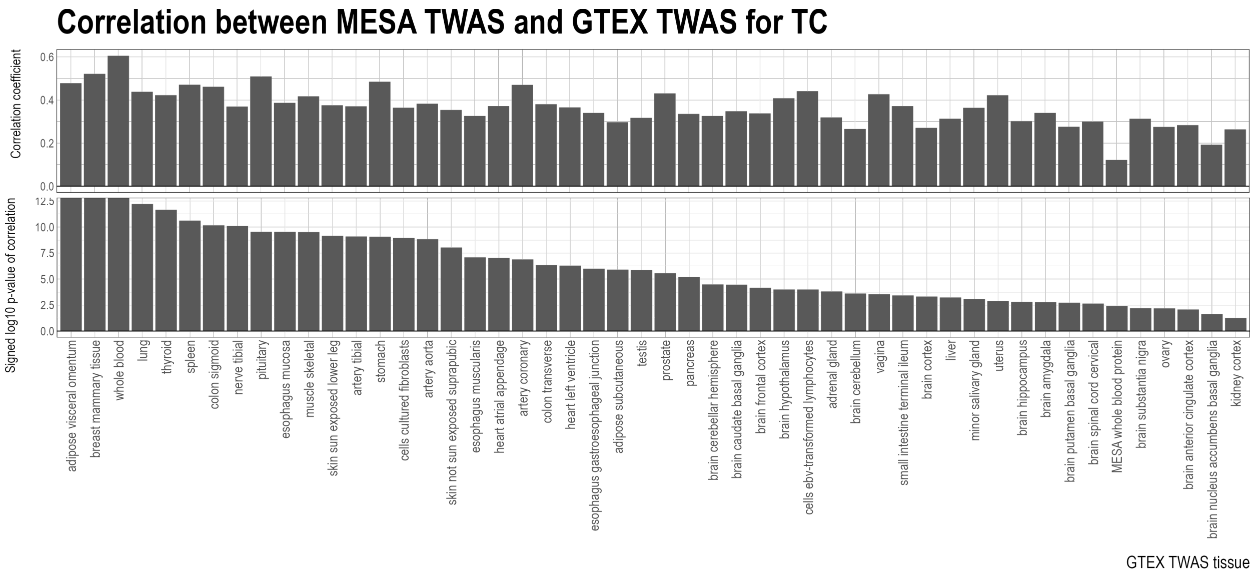

Figure S43: Correlation between signed log p-values of MESA PBMC TWAS for TG and signed log p-values of GTEx tissue-specific TWAS for TG.

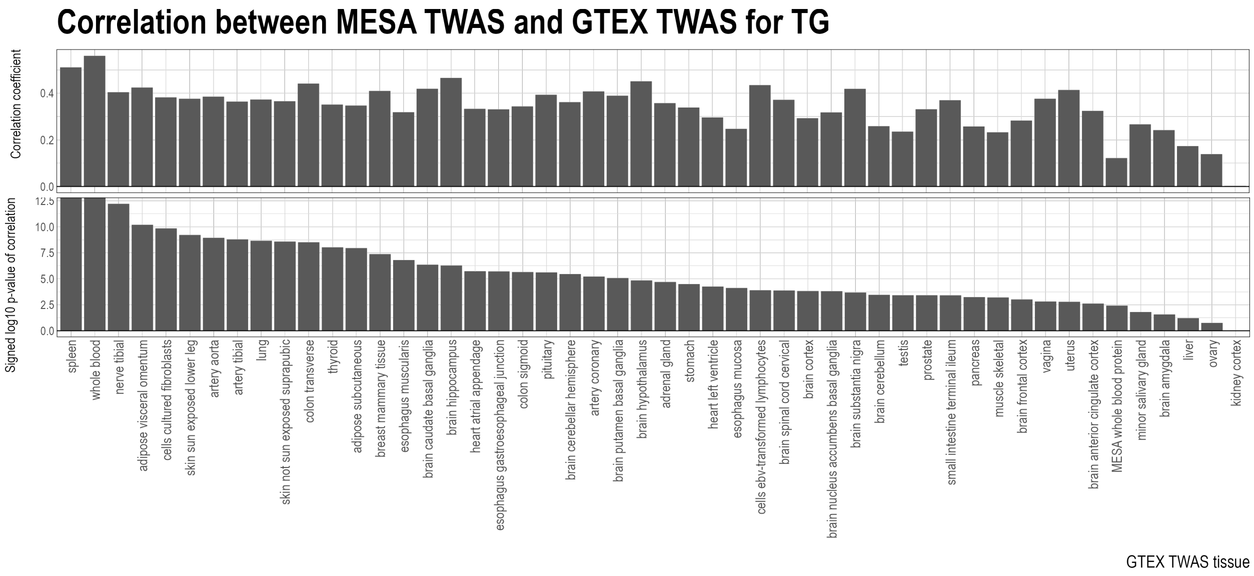

Figure S44: Correlation between signed log p-values of MESA PBMC TWAS for HDL and signed log p-values of GTEx tissue-specific TWAS for HDL.

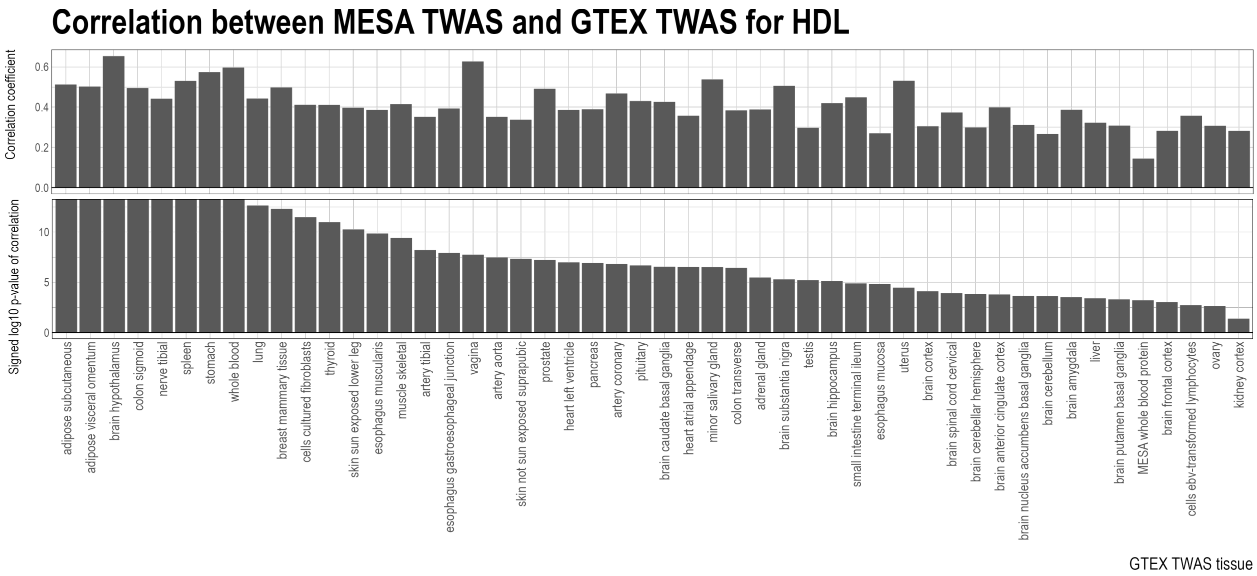

Figure S45: Correlation between signed log p-values of MESA PBMC TWAS for LDL and signed log p-values of GTEx tissue-specific TWAS for LDL.

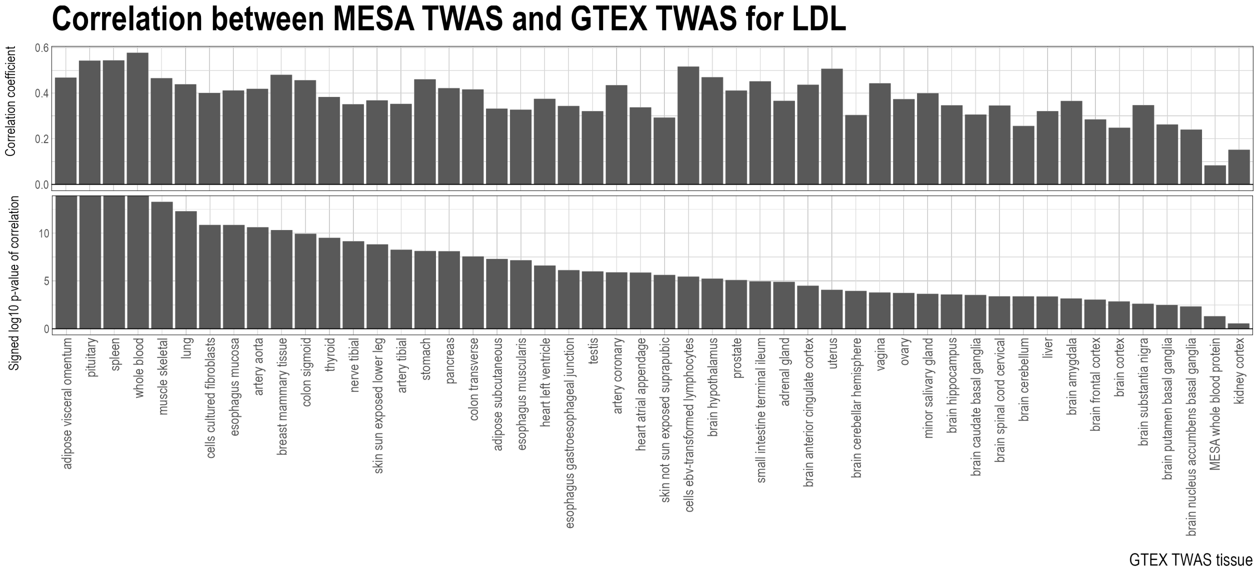

Figure S46: LDL GWAS and APOE protein QTL results used in the colocalization analysis.

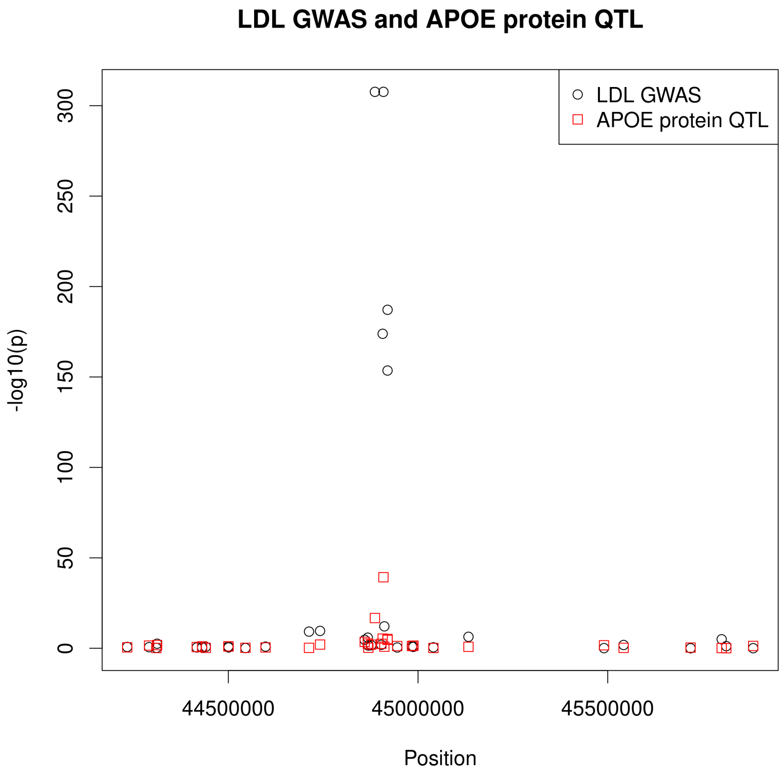

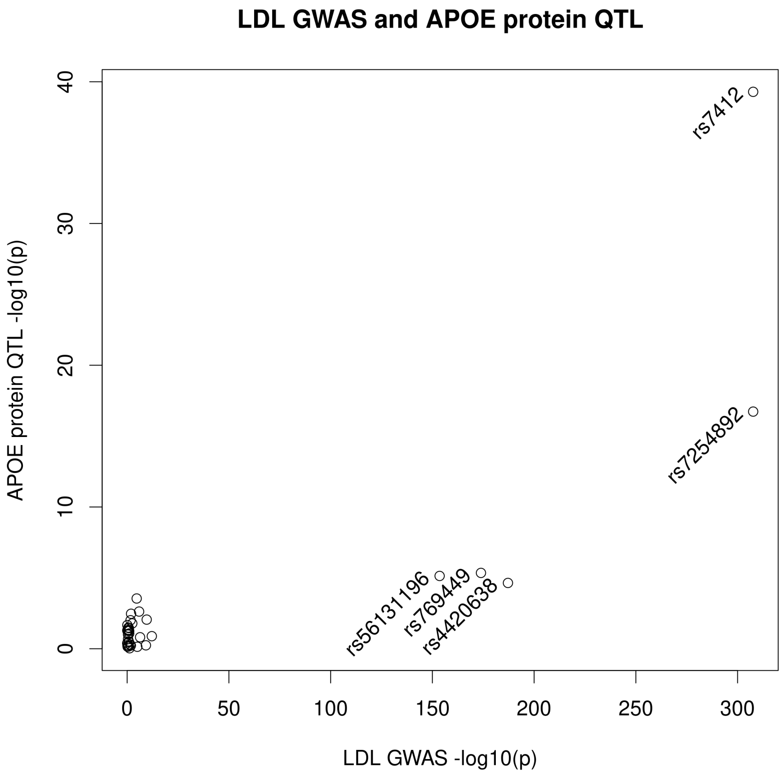

Table S1: Data characteristics of the MESA dataset. For continuous variables, the mean (and standard deviation) are displayed.

Self-reported race	Asian (7%)	Black (20%)	Hispanic (31%)	White (43%)
TC (mg/dl)	196 (29)	189 (39)	197 (35)	197 (33)
TG (mg/dl)	150 (74)	93 (42)	145 (66)	126 (63)
HDL (mg/dl)	49 (11)	52 (14)	48 (12)	53 (15)
LDL (mg/dl)	117 (26)	118 (34)	119 (33)	119 (30)
Age (mg/dl)	62 (10)	61 (10)	59 (09)	61 (10)
Sex (female)	45%	59 %	53 %	52 %
Using lipid medication	22%	13 %	12 %	19 %

Table S2: Colocalization analysis between GLGC GWAS, protein QTLs, and gene expression QTLs. Displayed are the probabilities computed by COLOC for insufficient power (P0+P1+P2), independent signals (P3), and colocalization (P4).

APOE	P0+P1+P2	P3	P4
TC-protein	0.00	0.00	1.00
TG-protein	0.00	1.00	0.00
HDL-protein	0.00	0.00	0.99
LDL-protein	0.00	0.00	1.00
TC-gene expression	0.94	0.01	0.03
TG-gene expression	0.91	0.01	0.07
HDL-gene expression	0.86	0.01	0.12
LDL-gene expression	0.94	0.01	0.03
protein-gene expression	0.94	0.01	0.03

FCGR2B	P0+P1+P2	P3	P4
TC-protein	0.03	0.00	0.95
TG-protein	0.99	0.00	0.00
HDL-protein	0.99	0.00	0.00
LDL-protein	0.03	0.00	0.96
TC-gene expression	0.02	0.00	0.96
TG-gene expression	0.99	0.00	0.00
HDL-gene expression	0.98	0.00	0.00
LDL-gene expression	0.02	0.00	0.97
protein-gene expression	0.00	1.00	0.00

LILRB2	P0+P1+P2	P3	P4
TC-protein	0.00	0.99	0.00
TG-protein	0.96	0.00	0.03
HDL-protein	0.00	1.00	0.00
LDL-protein	0.99	0.00	0.00
TC-gene expression	0.00	0.98	0.00
TG-gene expression	0.97	0.00	0.01
HDL-gene expression	0.00	1.00	0.00
LDL-gene expression	0.99	0.00	0.00
protein-gene expression	0.00	0.99	0.00

MICB	P0+P1+P2	P3	P4
TC-protein	0.00	0.62	0.37
TG-protein	0.00	0.99	0.00
HDL-protein	0.04	0.60	0.35
LDL-protein	0.01	0.95	0.03
TC-gene expression	0.00	1.00	0.00
TG-gene expression	0.00	1.00	0.00
HDL-gene expression	0.06	0.91	0.01
LDL-gene expression	0.01	0.96	0.02
protein-gene expression	0.00	1.00	0.00
